# Anti-PD-1 plus anti-CTLA-4 blockade overcomes immune exclusion in NSCLC brain metastases by enhancing CD8^+^ T cell responses and promoting tertiary lymphoid structure formation

**DOI:** 10.1038/s41467-026-74782-7

**Published:** 2026-07-07

**Authors:** Kazutaka Hosoya, Hiroaki Ozasa, Takahiro Tsuji, Masahiro Oi, Yusuke Shima, Keiichiro Suminaga, Kentaro Hashimoto, Hiroshi Yoshida, Tomoko Funazo, Hitomi Ajimizu, Takashi Nomizo, Hironori Yoshida, Hiroyuki Katsuragawa, Kentaro Tsuji, Noritaka Sano, Shigeki Takada, Yohei Mineharu, Shigeto Nishikawa, Toshi Menju, Akihiko Yoshizawa, Yoshiki Arakawa, Hiroaki Wake, Toyohiro Hirai

**Affiliations:** 1 Department of Respiratory Medicine, Kyoto University Graduate School of Medicine, Kyoto, Japan; 2 Department of Anatomy and Molecular Cell Biology, Nagoya University Graduate School of Medicine, Nagoya, Japan; 3 Department of Diagnostic Pathology, Kyoto University Hospital, Kyoto, Japan; 4 Department of Neurosurgery, Kyoto University Graduate School of Medicine, Kyoto, Japan; 5 Department of Digital Transformation in Healthcare and Medicine, Kyoto University Graduate School of Medicine, Kyoto, Japan; 6 Department of Thoracic Surgery, Kyoto University Graduate School of Medicine, Kyoto, Japan; 7 Department of Diagnostic Pathology, Nara Medical University, Kashihara, Japan; 8 Division of Multicellular Circuit Dynamics, National Institute for Physiological Sciences, Okazaki, Japan; 9 Department of Physiological Sciences, Graduate University for Advanced Studies, SOKENDAI, Hayama, Japan

**Keywords:** Cancer microenvironment, Non-small-cell lung cancer, Cancer immunotherapy

## Abstract

Brain metastases (BrMs) in non-small cell lung cancer (NSCLC) respond poorly to anti-PD-1 monotherapy, but the underlying immune resistance remains incompletely defined. Here we integrate clinical outcome analyses, paired human tissue profiling and syngeneic mouse models to characterize the BrM immune microenvironment. Clinical analyses suggest improved intracranial disease control with nivolumab plus ipilimumab compared with nivolumab alone. Paired human specimens show that BrMs contain fewer cytotoxic T lymphocytes (CTLs) and tertiary lymphoid structures (TLSs) than primary tumors, defining an immune-excluded phenotype. A syngeneic BrM model recapitulates this phenotype and resists anti-PD-1 monotherapy, whereas combined anti-PD-1 and anti-CTLA-4 blockade suppresses tumor growth and prolongs survival. Single-cell RNA sequencing, flow cytometry and immunofluorescence show increased CTL infiltration and effector function after combination therapy. CD8^+^ T cell depletion abrogates therapeutic benefit, and combination therapy expands T follicular helper-like cells and induces TLS-like structures. These findings manifest increased adaptive immune response of dual checkpoint blockade in NSCLC BrMs.

## Abstract (web summary)

The immune profile of dual PD1/CTLA-4 blockade in brain metastases in non-small cell lung cancer (NSCLC) remain to be investigated. The authors here show that dual PD1/CTLA-4 blockade remodels the brain metastasis microenvironment by enhancing CD8^+^ T cell responses and promoting TLS-like structures.

## Introduction

Lung cancer is the leading cause of cancer-related deaths worldwide^1^. Non-small cell lung cancer (NSCLC) accounts for approximately 80% of all lung cancer cases^2^, with most patients diagnosed at an advanced stage. Brain metastases (BrMs) develop in approximately 40% of NSCLC patients during the disease course and have a profound impact on prognosis and quality of life (QoL)^3^. BrMs directly contribute to mortality in a substantial proportion of patients, with central nervous system (CNS) metastases, including BrMs, reported as the direct cause of death in up to 33% of cases^4^. Furthermore, as recently developed therapies, such as tyrosine kinase inhibitors (TKIs) and immune checkpoint inhibitors (ICIs), extend overall survival, preserving quality of life and preventing new BrM development have become increasingly critical clinical priorities. Therefore, CNS-effective treatments are urgently needed to address both the life-threatening nature of BrM and the long-term QoL of patients.

Clinical trials have shown that ICIs improve survival in metastatic NSCLC. However, anti-PD-1 monotherapy has shown limited efficacy against BrMs, particularly in patients with active lesions^5^. In melanoma, combination ICI therapy, especially anti-PD-1 plus anti-CTLA-4, has achieved superior outcomes compared with anti-PD-1 alone^6,7^. In NSCLC, the CheckMate 227 trial demonstrated that the combination of anti-PD-1 and anti-CTLA-4 achieved superior intracranial responses compared with cytotoxic chemotherapy^8^. Moreover, a prospective trial demonstrated the promising efficacy of anti-PD-1 and anti-CTLA-4 combined with cytotoxic chemotherapy in NSCLC patients with BrM^9^. Despite these encouraging findings, direct comparisons of anti-PD-1 monotherapy versus combination therapy in NSCLC patients with BrMs are lacking. Furthermore, the mechanistic contribution of CTLA-4 blockade in BrMs has not been thoroughly investigated in clinical or preclinical settings.

The effectiveness of immunotherapy is strongly influenced by the tumor microenvironment (TME)^10^, including features such as the formation of tertiary lymphoid structures (TLSs)^11^. In NSCLC BrMs, surgical resection is performed in relatively few patients, limiting tissue availability and subsequent investigations. Studies conducted to date have reported reduced infiltration of tumor-infiltrating lymphocytes (TILs), particularly CD8⁺ cytotoxic T lymphocytes (CTLs) and regulatory T cells (Tregs), in BrMs compared with extracranial metastases^12–16^. Since CTLs are a primary target of anti-PD-1 therapy, their scarcity in BrMs may partly explain the limited efficacy of PD-1 blockade alone.

Preclinical studies in melanoma BrM models have shown that PD-1 blockade alone has limited efficacy, whereas the addition of CTLA-4 blockade enhances tumor control^17–19^. This benefit has been linked to improved trafficking of CTLs into BrMs. However, the effects of combination therapy in NSCLC BrM models remain poorly understood, and the specific role of CTLA-4 blockade in shaping the BrM immune microenvironment has not been fully clarified.

To address these gaps, we define the immune microenvironment of NSCLC BrMs using paired clinical specimens, clinical outcome analyses, and syngeneic mouse models, with a focus on CTL exclusion and TLS formation. We show that NSCLC BrMs display an immune-excluded phenotype with reduced CTL infiltration and TLS formation, and that combined anti-PD-1 and anti-CTLA-4 blockade remodels this microenvironment toward enhanced intracranial antitumor immunity. These findings provide a mechanistic framework for understanding the limited activity of anti-PD-1 monotherapy in NSCLC BrMs. They also identify immune exclusion and TLS insufficiency as potential targets for improving immunotherapeutic control of NSCLC BrMs.

## Results

### Differential CNS outcomes with nivolumab versus nivolumab plus ipilimumab in patients with NSCLC

**Fig. 1:**
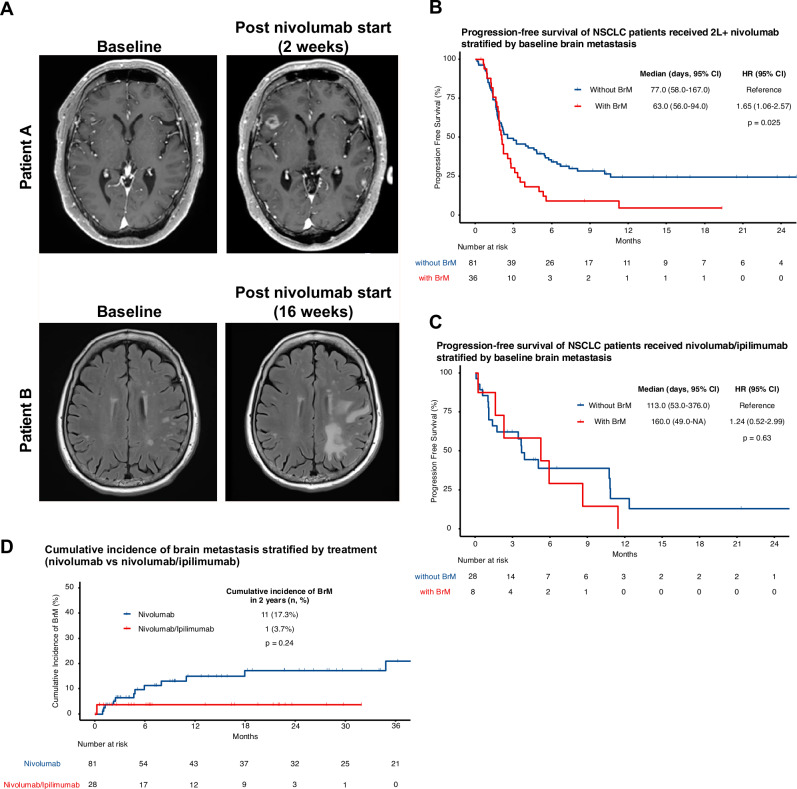
Nivolumab plus ipilimumab overcomes the negative prognostic impact of BrMs in NSCLC patients. **A** Brain MRI images showing the clinical course of nivolumab treatment in NSCLC patient A (top: axial contrast-enhanced T1-weighted images) and NSCLC patient B (bottom: axial FLAIR images). For each patient, images at baseline and after the start of treatment are shown. **B** Kaplan–Meier analysis showing that patients with baseline BrM had a significantly shorter PFS when treated with nivolumab monotherapy. Statistical significance was assessed using a two-sided log-rank test. Source data are provided as a Source data file. **C** Kaplan–Meier analysis showing that PFS was not significantly different between patients with or without baseline BrM when treated with nivolumab plus ipilimumab. Statistical significance was assessed using a two-sided log-rank test. Source data are provided as a Source data file. **D** Cumulative incidence of new BrM development in NSCLC patients receiving nivolumab monotherapy or nivolumab plus ipilimumab combination and without baseline BrM, showing a lower trend in patients receiving combination therapy compared with nivolumab monotherapy. Statistical significance was assessed using a two-sided log-rank test. Source data are provided as a Source data file. BrM brain metastasis, PFS progression-free survival. *P < 0.05.

To assess the efficacy of immunotherapy against BrMs within the context of current standard treatments, we conducted a single-center retrospective analysis of CNS outcomes. The baseline characteristics of the nivolumab monotherapy and nivolumab plus ipilimumab cohorts are shown in Supplementary Data 3 and 4, respectively. Patients were stratified according to the presence or absence of brain metastases at the time of ICI initiation (defined as “baseline BrM”), based on review of radiographic assessments obtained between the diagnosis of NSCLC and the start of ICI therapy. Brain-directed radiotherapy for brain metastases was delivered exclusively as focal irradiation (no whole-brain radiotherapy) between the diagnosis of brain metastases and ICI initiation in 64% of patients in the nivolumab cohort and 56% in the nivolumab plus ipilimumab cohort. For response analyses, we excluded patients who received brain radiotherapy within 30 days prior to ICI initiation to minimize potential confounding effects on intracranial response assessment; these patients were retained in the PFS and OS analyses. Representative brain MRI images illustrate the rapid progression of BrMs shortly after nivolumab initiation in one patient (Fig. 1A). Kaplan–Meier analysis of PFS revealed that patients receiving nivolumab monotherapy had significantly shorter PFS when BrMs were present at baseline compared with those without BrMs (Fig. 1B, median, 63 vs. 77 days; HR, 1.65 [95% CI 1.06–2.57], *p* = 0.025), underscoring the limited efficacy of anti-PD-1 monotherapy in this setting. In contrast, no significant difference in PFS was observed in patients treated with nivolumab plus ipilimumab, despite the smaller cohort size due to later approval (Fig. 1C, median, 160 vs. 113 days; HR 1.24 [95% CI: 0.52–2.99], *p* = 0.63). OS showed similar trends between patients with and without BrMs in both the monotherapy and combination therapy groups (Supplementary Fig. 1A, B). In analyses including all patients regardless of baseline BrM status, PFS and OS were comparable between the nivolumab and nivolumab plus ipilimumab groups (Supplementary Fig. 1C, D). Among patients with baseline BrMs, PFS was also comparable between treatment groups (Supplementary Fig. 1E). However, OS was significantly shorter in the nivolumab plus ipilimumab group (Supplementary Fig. 1F, median, 15.9 vs. 6.9 months; HR 2.53 [95% CI: 1.10–5.82], *p* = 0.023). This finding likely reflects differences in patient characteristics between the two cohorts, including a high proportion of patients with driver oncogene alterations in the nivolumab cohort and patients with PD-L1-negative tumors in the nivolumab plus ipilimumab group, rather than a detrimental effect of combination therapy per se (see “Discussion”). Notably, when restricting the analysis to patients without driver oncogene alterations (predominantly EGFR mutations), PFS and OS were comparable between treatment groups (Supplementary Fig. 1G, H, OS median, 9.8 vs. 5.9 months; HR 1.55 [95% CI: 0.64–3.71], *p* = 0.32).

The systemic efficacy based on RECIST evaluation for both cohorts (nivolumab monotherapy and nivolumab plus ipilimumab combination therapy) is summarized in Supplementary Data 5. Combination therapy demonstrated promising intracranial disease control. While intracranial objective response rates were similar between monotherapy and combination groups (14% vs. 17%, *p* = 1.00), the intracranial disease control rate showed a trend toward a higher rate with combination therapy (32% vs. 67%, *p* = 0.174), although statistical inference was limited by the small sample size and the difference did not reach statistical significance (Supplementary Data 6). These findings suggest potentially enhanced intracranial efficacy of anti-PD-1 plus anti-CTLA-4 therapy. Furthermore, the cumulative incidence of new BrM development showed a trend toward a lower rate in patients receiving combination therapy than in those on nivolumab monotherapy (cumulative incidence of BrM in 2 years, 17.3% vs. 3.7%, *p* = 0.24), although the difference did not reach statistical significance (Fig. 1D and Supplementary Fig. 1I), suggesting possible reduced CNS progression risk. Regarding safety, grade ≥3 treatment-related adverse events occurred significantly more frequently in the combination group than in the monotherapy group (36% vs. 13%, *p* = 0.003, Supplementary Data 7), consistent with prior reports for this drug class^20,21^. Overall, these results suggest that nivolumab plus ipilimumab may provide superior intracranial disease control in NSCLC patients with or without BrMs, while highlighting the increased risk of high-grade adverse events.

### Transcriptomic analysis indicates a distinct immune microenvironment in NSCLC BrMs compared with primary tumors

**Fig. 2:**
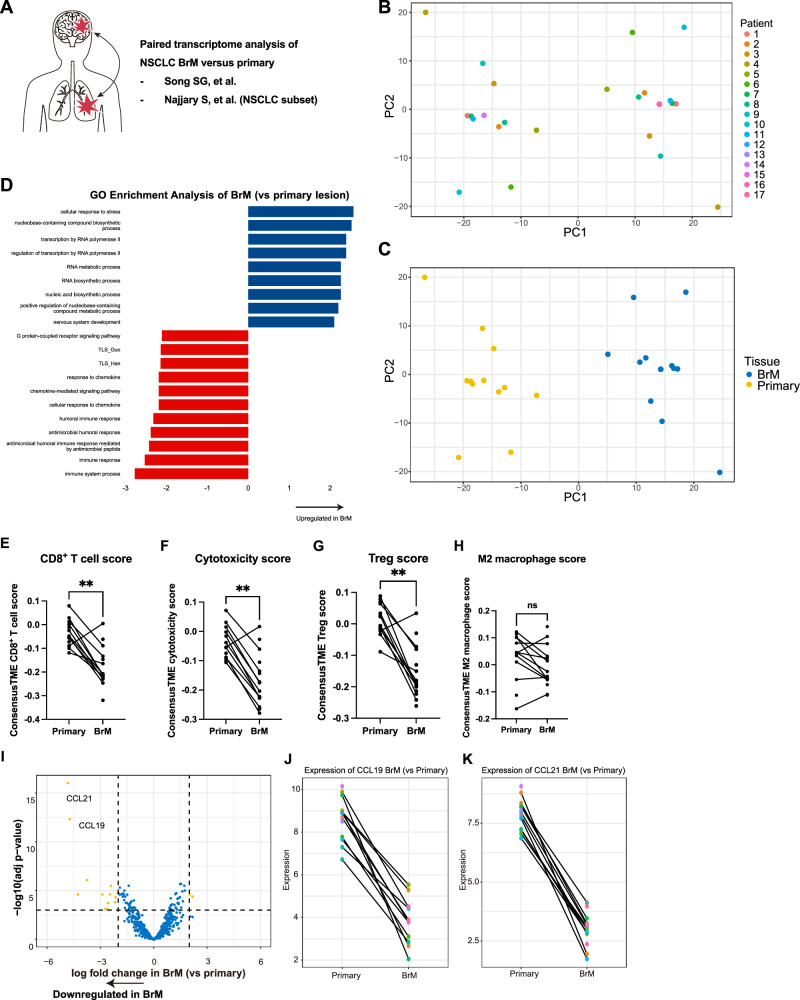
Transcriptomic analysis reveals an immunosuppressive microenvironment in NSCLC BrM. **A** Overview of the study design using publicly available transcriptome datasets of paired primary tumor and BrM samples. PCA reveals that gene expression profiles are clearly separated by tissue type (**C** primary vs. BrM) rather than by individual patient origin (**B**). Source data are provided as a Source data file. **D** Gene set enrichment analysis (GSEA) reveals that GO pathways related to immune response and tertiary lymphoid structures are downregulated in BrM compared with paired primary tumors. Normalized enrichment score (NES) is shown. Source data are provided as a Source data file. **E**–**H** Gene set variation analysis (GSVA) scores show that scores of CD8^+^ T cells (**E**, *P* = 0.029), cytotoxicity (**F**, *P* = 0.0020), and Tregs (**G**, *P* = 0.0020) are significantly reduced in BrM compared with paired primary tumors. The M2 macrophage score is shown in (**H**, *P* = 0.0537). Data are derived from public transcriptome data from *n* = 17 patients, including *n* = 11 paired primary tumor and BrM samples used for paired comparison. Statistical significance was assessed using a two-sided Wilcoxon matched-pairs signed-rank test. Source data are provided as a Source data file. **I** Volcano plot highlighting that chemokines CCL19 and CCL21 are among the most significantly downregulated genes in BrM. Data are derived from public transcriptome data from *n* = 17 patients, including *n* = 11 paired primary tumor and BrM samples used for paired comparison. Source data are provided as a Source data file. Paired analysis confirms the decreased expression of CCL19 (**J**) and CCL21 (**K**) in BrM compared with primary tumors. Source data are provided as a Source data file. BrM brain metastasis, PCA principal component analysis, GSEA gene set enrichment analysis, GO gene ontology, NES normalized enrichment score, GSVA gene set variation analysis, Treg regulatory T cell. **P < 0.01.

To characterize the BrM immune microenvironment, we analyzed transcriptomic profiles of paired primary NSCLC and BrM samples using publicly available datasets (Fig. 2A)^22,23^. In Song’s dataset^22^, PCA of batch-corrected data showed clear separation by tissue origin (BrM vs. primary tumor) rather than by patient, suggesting systematic biological differences between sites (Fig. 2B, C). GO enrichment analysis revealed significant downregulation of pathways related to immune responses and chemokine signaling in BrMs compared with paired primary tumors (Fig. 2D). Consensus-TME-based deconvolution^24^ demonstrated significantly reduced CD8^+^ T cell, cytotoxicity, and Treg scores in BrMs, while M2 macrophage scores remained comparable (Fig. 2E–H). Differential gene expression analysis highlighted marked downregulation of the chemokines *CCL19* and *CCL21* in BrMs (Fig. 2I), with consistent findings across all patients (Fig. 2J, K). CCL19 and CCL21 are critical for lymphoid structure formation, including TLSs, in cancer^25,26^. TLSs are ectopic lymphoid aggregates composed mainly of B cells, T cells, and myeloid cells. Their presence has been linked to improved immunotherapy responses and prognosis in multiple solid tumors, including NSCLC^11,27^. To validate this, we assessed established TLS gene signatures from previous studies^28,29^ and found that TLS signature scores were significantly lower in BrMs than in paired primary tumors (Fig. 2D), supporting impaired TLS formation in BrMs.

These findings were consistent across an independent dataset, including results of PCA, GO enrichment analysis, immune cell subset scores, and DEG analysis (Supplementary Fig. 2A–J).

In summary, our transcriptomic analyses suggest that NSCLC BrMs have reduced immune cell infiltration, downregulation of the chemokines *CCL19* and *CCL21*, and impaired TLS development compared with primary tumors. These findings may explain the limited efficacy of ICI monotherapy in the CNS.

### Histological confirmation of reduced CTL infiltration and TLS formation in NSCLC BrMs

**Fig. 3:**
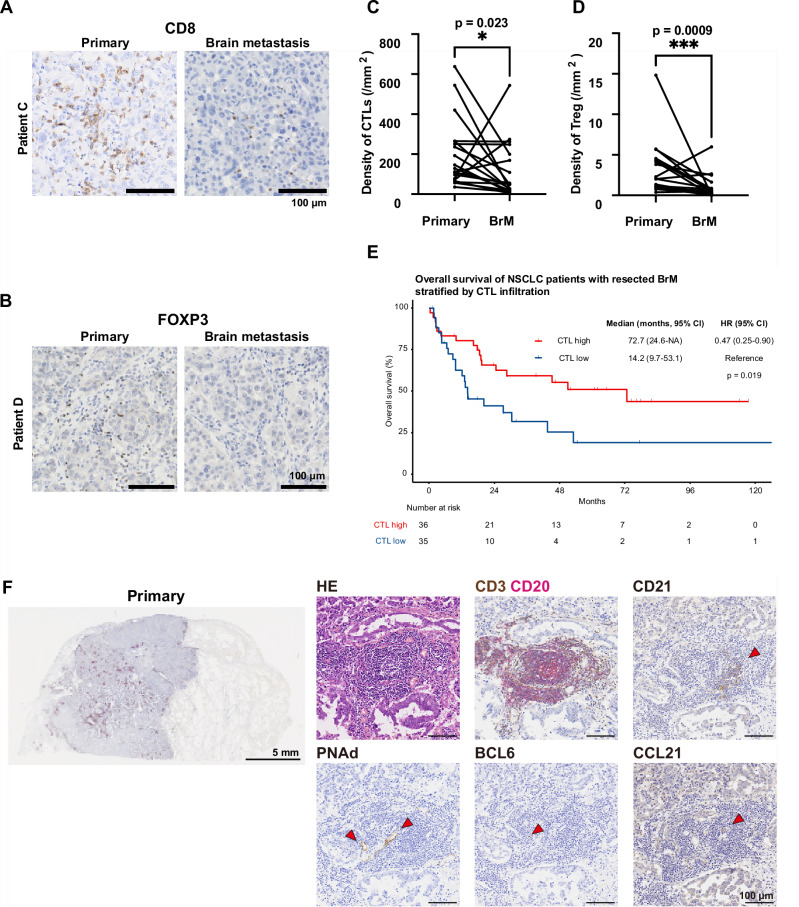

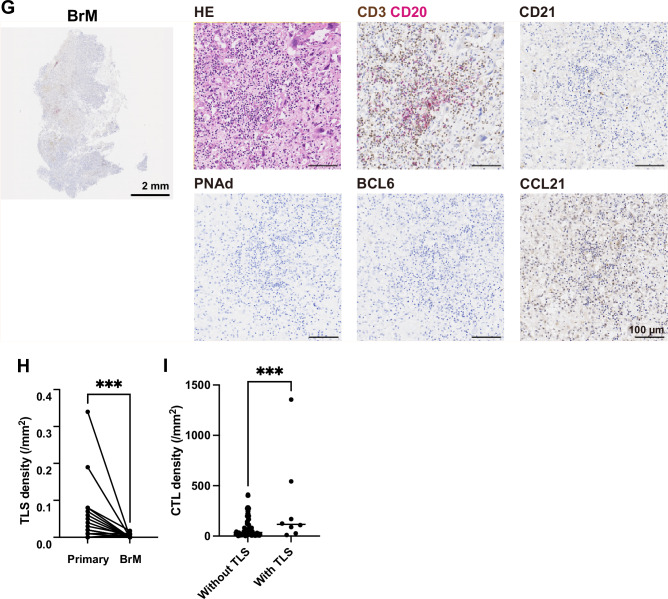
NSCLC BrM exhibit reduced infiltration of cytotoxic T lymphocytes and tertiary lymphoid structures. Representative images of immunostaining for CD8 (**A**) and FOXP3 (**B**) in paired primary and BrM samples. **C** Quantification showing that cytotoxic T lymphocyte (CTL) density was significantly reduced in BrM compared with paired primary tumors. Data are derived from *n* = 19 biologically independent paired patient samples. Statistical significance was assessed using a two-sided paired Wilcoxon matched-pairs signed-rank test. Source data are provided as a Source data file. **D** Quantification showing that regulatory T cell (Treg) density was also significantly lower in BrM compared with paired primary tumors. Data are derived from *n* = 19 biologically independent paired patient samples. Statistical significance was assessed using a two-sided paired Wilcoxon matched-pairs signed-rank test. Source data are provided as a Source data file. **E** Kaplan–Meier analysis demonstrating that high CTL infiltration in resected BrM was associated with significantly improved post-resection overall survival (OS). Statistical significance was assessed using a two-sided log-rank test. Source data are provided as a Source data file. Representative H&E and immunohistochemical images of a tertiary lymphoid structure (TLS) in a primary tumor (**F**) and its absence in the corresponding BrM (**G**). In **F**, red arrowheads indicate marker-positive cells or structures within the TLS. **H** Quantification showing that TLS density was significantly lower in BrM compared with paired primary tumors (*P* = 0.0003). Data are derived from *n* = 18 biologically independent paired patient samples. Statistical significance was assessed using a two-sided paired Wilcoxon matched-pairs signed-rank test. Source data are provided as a Source data file. **I** Comparison showing that BrM containing TLS had significantly higher CTL infiltration than BrM without TLS (*P* = 0.0005). Data are derived from *n* = 8 TLS-positive and *n* = 77 TLS-negative biologically independent BrM samples. Statistical significance was assessed using a two-sided paired Wilcoxon matched-pairs signed-rank test. BrM brain metastasis, CTL cytotoxic T lymphocyte, Treg regulatory T cell, OS overall survival, TLS tertiary lymphoid structure. *P < 0.05, ***P < 0.001.

To histologically validate our transcriptomic findings, we examined surgically resected specimens from NSCLC patients with paired primary tumors and BrMs. Patient characteristics for cases with paired samples are summarized in Supplementary Data 8. Immunohistochemical analysis revealed significantly reduced density of CTLs (defined as CD8a-positive immune cells) in BrMs compared with paired primary tumors (Fig. 3A, C). Similarly, Treg infiltration, defined by FOXP3-positive immune cells, was also significantly lower in BrMs (Fig. 3B, D), consistent with our transcriptomic analysis. To further investigate the clinical relevance of immune cell infiltration, we expanded our analysis to include all NSCLC patients with surgically resected BrM with and without paired primary tumors. Patient characteristics stratified by CTL and Treg density are provided in Supplementary Data 9 and 10, respectively. Kaplan–Meier analysis demonstrated that high CTL infiltration in BrMs was significantly associated with improved post-resection overall survival (Fig. 3E). This association remained significant in both univariable and multivariable Cox regression analyses (Supplementary Data 11) and was consistent when CTL infiltration was analyzed as a log-transformed continuous variable (Supplementary Data 12). In contrast, Treg density showed no significant correlation with patient outcomes (Supplementary Fig. 3A). One notable outlier case further illustrated the potential prognostic value of CTLs in BrM. This patient who displayed markedly higher CTL density in the BrM than in the primary tumor (543.7 vs. 78.0 cells/mm^2^, Supplementary Fig. 3B), experienced a remarkable long-term survival of over seven years without disease progression following BrM resection. While this represents a single case observation rather than definitive evidence, it supports the broader observation that CTL infiltration may be functionally relevant in the BrM microenvironment.

We next examined TLS formation using H&E staining, with confirmation by immunohistochemical staining for CD3 and CD20 in representative sections (Fig. 3F, G and Supplementary Fig. 3C–F). To further characterize TLS features beyond CD3 and CD20, we performed additional immunohistochemical staining for CD21, PNAd, BCL6, and CCL21. In primary tumors, we observed focal aggregates of B and T lymphocytes accompanied by a CD21^+^ follicular dendritic cell network and PNAd^+^ endothelial structures consistent with HEV-like features; a small subset of cells showed BCL6 positivity, and CCL21^+^ cells were also detected. (Fig. 3F, G, and Supplementary Fig. 3E). Quantitative analysis revealed that TLS density was significantly lower in BrMs than in paired primary tumors (Fig. 3H). Importantly, BrMs containing TLSs had significantly higher CTL infiltration than TLS-negative BrMs (Fig. 3I), consistent with the functional link between TLSs and T cell recruitment. However, TLS presence in BrMs was not significantly associated with post-resection survival and did not show a clear trend toward improved outcome (Supplementary Fig. 3G). This analysis was based on section-level assessment of resected BrM specimens and could have underestimated TLS positivity due to limited sampling of the tumor periphery, where TLSs are often enriched, particularly given the constraints of brain metastasis surgery aimed at minimizing injury to adjacent normal brain tissue.

These findings indicate that although CTL infiltration and TLS formation are reduced in BrMs, TLSs retain the ability to recruit CTLs when present, and these CTLs remain functionally relevant, as reflected in their association with improved prognosis. Collectively, these results suggest that strategies aimed at enhancing CTL infiltration and/or promoting TLS formation may represent promising therapeutic approaches for BrMs in NSCLC.

### Mouse model of NSCLC BrM by internal carotid artery injection reveals therapeutic effect of combined anti-PD-1 and anti-CTLA-4 therapy

**Fig. 4:**
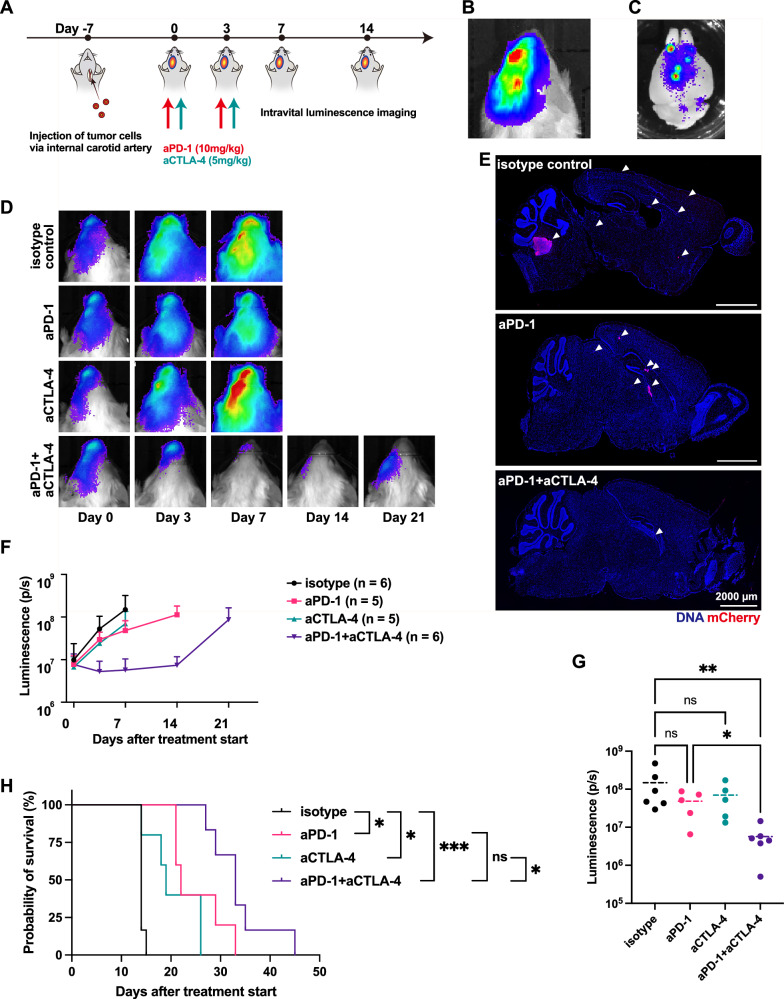
Anti-PD-1 plus anti-CTLA-4 combination therapy shows efficacy in a murine NSCLC BrM model. **A** Schematic representation of the murine BrM model established via ICA injection. Representative in vivo (**B**) and ex vivo (**C**) luminescence images showing intracranial tumor establishment. **D** Representative longitudinal in vivo luminescence images of BrM-bearing mice treated with the isotype control, anti-PD-1, anti-CTLA-4, or combination therapy. **E** Representative immunofluorescence images of BrM showing reduced tumor area in the combination therapy group. White arrowheads indicate mCherry-positive tumor foci. **F** Longitudinal analysis of tumor burden, measured by luminescence signals, showing potent tumor inhibition with combination therapy (mean ± SD; isotype, *n* = 6; aPD-1, *n* = 5; aCTLA-4, *n* = 5; aPD-1 + aCTLA-4, *n* = 6). Data are combined from 3 independent experiments. Source data are provided as a Source data file. **G** Comparison of tumor burden on day 7. Combination therapy significantly reduced tumor burden compared with the isotype control and both monotherapies. Each dot represents data from an individual mouse (mean ± SD; isotype, *n* = 6; aPD-1, *n* = 5; aCTLA-4, *n *= 5; aPD-1 + aCTLA-4, *n* = 6). Data are combined from 3 independent experiments. Statistical significance was assessed using Kruskal–Wallis followed by Dunn’s test. Pairwise *P* values were as follows: isotype vs. aPD-1, *P* = 0.3644; isotype vs. aCTLA-4, *P* = 0.5816; isotype vs. aPD-1 + aCTLA-4, *P* = 0.0010; aPD-1 vs. aPD-1 + aCTLA-4, *P* = 0.0258. Source data are provided as a Source data file. **H** Kaplan–Meier survival curves. Combination therapy significantly prolonged survival compared with the isotype control. The survival difference between combination therapy and anti-PD-1 monotherapy did not reach statistical significance in this model. (isotype, *n* = 6; aPD-1, *n* = 5; aCTLA-4, *n* = 5; aPD-1 + aCTLA-4, *n* = 6). Data are combined from 3 independent experiments. Statistical significance was assessed using a two-sided log-rank test. Pairwise *P* values were as follows: isotype vs. aPD-1, *P* = 0.0009; isotype vs. aCTLA-4, *P* = 0.0079; isotype vs. aPD-1 + aCTLA-4, *P* = 0.0005; aPD-1 vs. aPD-1 + aCTLA-4, *P* = 0.0512; aCTLA-4 vs. aPD-1 + aCTLA-4, *P* = 0.0011. Source data are provided as a Source data file. BrM brain metastasis, ICA internal carotid artery. *P < 0.05, **P < 0.01, and ***P < 0.001.

To model BrM in a clinically relevant context, we used immunocompetent C57BL/6 mice and syngeneic lung adenocarcinoma cell lines (CMT167 and LLC). We first established stable CMT167 and LLC cell lines expressing mCherry and Akaluc luciferase, enabling both fluorescence and BLI (Supplementary Fig. 4A). Using these cell lines, we generated a BrM model via internal carotid artery injection (Fig. 4A). Successful intracranial tumor formation was confirmed in vivo (Fig. 4B) and ex vivo (Fig. 4C) by BLI (data shown for CMT167). Notably, while anti-PD-1 or anti-CTLA-4 antibody monotherapies showed limited efficacy compared with isotype control, combination therapy significantly inhibited tumor growth (Fig. 4D, F, G, and Supplementary Fig. 4B) and prolonged survival compared with isotype control (Fig. 4H), without significant body weight loss (Supplementary Fig. 4C). Immunofluorescence of brain tissues collected seven days after treatment initiation confirmed reduced tumor burden in combination-treated mice (Fig. 4E and Supplementary Fig. 4D, E). These results were reproduced in an alternative BrM model using LLC cells (Supplementary Fig. 4F–J). In the CMT167 ICA model, survival did not differ significantly between the combination therapy and anti-PD-1 monotherapy groups (Fig. 4H). By contrast, in the LLC ICA model, combination therapy prolonged survival compared with anti-PD-1 monotherapy (Supplementary Fig. 4I). Histological analysis showed that internal carotid artery injection consistently led to carcinomatous meningitis, with tumor cells infiltrating the ventricles (Supplementary Fig. 4K). This meningeal involvement is consistent with a known feature of ICA-based delivery models, in which tumor cells may access CSF compartments in addition to forming parenchymal lesions. Combination therapy tended to suppress both parenchymal metastasis and carcinomatous meningitis (Supplementary Fig. 4L, M).

In contrast, in a primary lung tumor model established by tail vein injection (Supplementary Fig. 5A), both anti-PD-1 and anti-CTLA-4 monotherapies exhibited significant antitumor activity, with further enhancement by combination therapy (Supplementary Fig. 5B–F). No treatment-related toxicity was observed, as measured by body weight (Supplementary Fig. 5G). Importantly, baseline CD8^+^ T cell density was significantly lower in BrMs than in primary lung tumors (Supplementary Fig. 5H, I), suggesting that the differential therapeutic responses may be due to differences in the immune microenvironment. Monotherapy efficacy was confirmed in additional experiments using LLC cells (Supplementary Fig. 5J–M).

These findings parallel our clinical observations, where anti-PD-1 monotherapy was less effective in BrMs than in primary tumors, whereas anti-PD-1 plus anti-CTLA-4 showed superior intracranial control. Our murine models recapitulate the clinical observation of lower CTL infiltration in BrMs, supporting a mechanistic explanation for site-specific treatment responses. Collectively, these data support the use of our syngeneic mouse models as appropriate for studying the BrM immune microenvironment and provide further rationale for combination immunotherapy in NSCLC BrMs.

### scRNA-seq indicates combination therapy reshapes the BrM immune microenvironment and enhances CTL effector phenotype

**Fig. 5:**
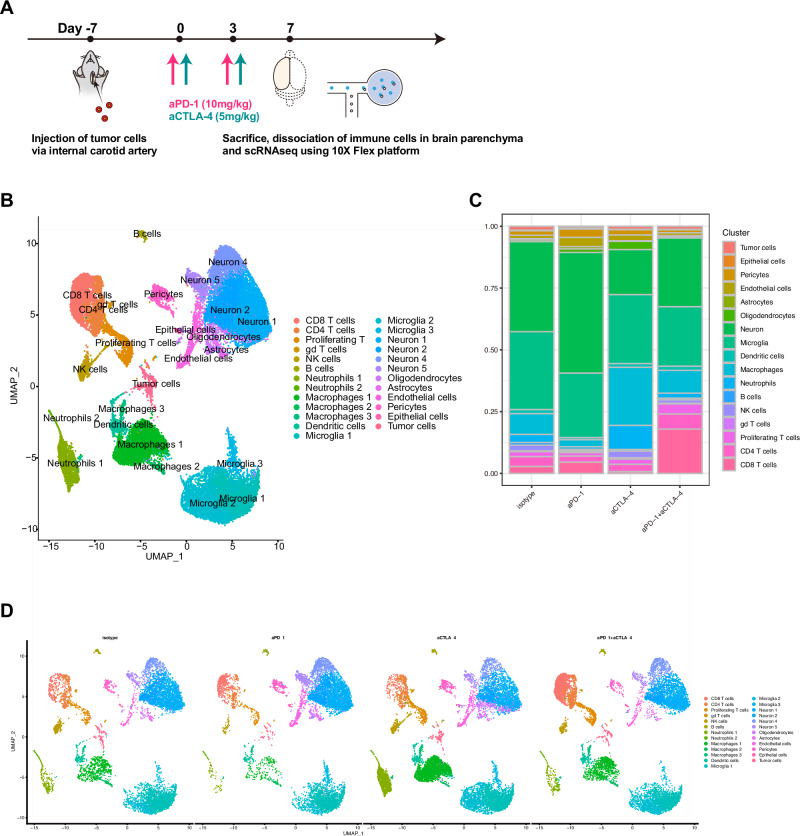

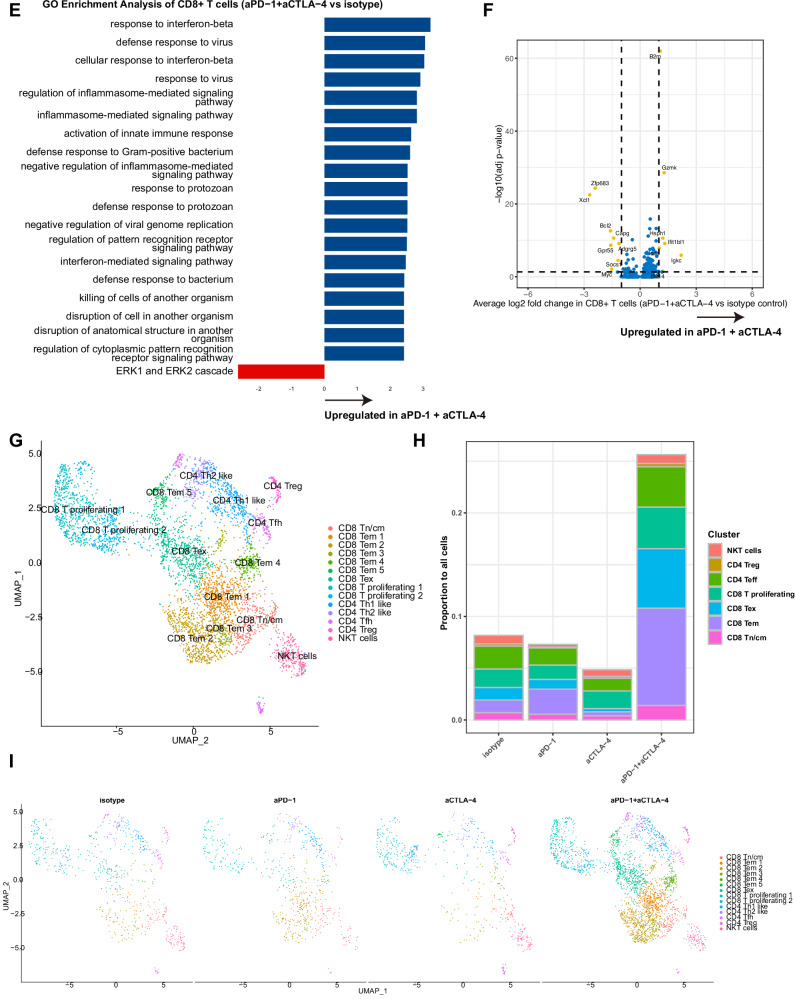
Single-cell RNA sequencing reveals combination therapy enhances CTL infiltration and effector function in BrM. **A** Schematic representation of the scRNA-seq workflow for BrM tissues harvested from treated mice. **B** UMAP visualization of all single cells from BrM, identifying major immune and CNS cell populations (one pooled scRNA-seq library per treatment group); each library was generated from pooled CMT167 BrM-bearing brains (isotype, *n* = 4; aPD-1, *n* = 4; aCTLA-4, *n* = 3; aPD-1+aCTLA-4, *n* = 4). **C** Stacked bar plot of cellular composition across treatment groups, showing that combination therapy markedly increased the proportion of CD8^+^ T cells. Source data are provided as a Source data file. **D** UMAP plots split by treatment group, showing expansion of the T cell compartment following combination therapy. **E** GO enrichment analysis of CTLs revealed upregulation of pathways related to interferon response and cytotoxicity in the combination therapy group compared with the isotype control. NES is shown. Source data are provided as a Source data file. **F** Volcano plot of DEGs in CTLs from the combination therapy group, showing upregulation of genes associated with T cell activation and effector function. Source data are provided as a Source data file. **G** UMAP visualization of re-clustered T cells, identifying distinct functional subsets. Stacked bar plot (**H**) and corresponding UMAP visualizations (**I**) showing expansion of effector T-cell populations in the combination therapy group. Source data are provided as a Source data file. BrM brain metastasis, scRNAseq single-cell RNA sequencing, UMAP uniform manifold approximation and projection, CNS central nervous system, GO gene ontology, CTL cytotoxic T lymphocyte, NES normalized enrichment score, DEG differentially expressed gene.

To investigate the cellular mechanisms underlying the enhanced efficacy of anti-PD-1 and anti-CTLA-4 combination therapy in BrM, we performed scRNA-seq on immune cells isolated from the brain parenchyma of CMT167 BrM-bearing mice treated with isotype control, anti-PD-1, anti-CTLA-4, or combination therapy (Fig. 5A); because the yield of immune cells from BrM-bearing brains was limited (~1−3 × 10^5^ cells per mouse), samples were pooled to generate sufficient input for scRNA-seq. Accordingly, these scRNA-seq analyses are interpreted as descriptive and hypothesis-generating, and treatment-associated differences should be considered as trends. After batch-effect correction, unbiased clustering and marker gene-based annotation identified distinct immune cell populations, including T cells, myeloid cells (microglia, macrophages, dendritic cells, and neutrophils), B cells, and NK cells, as well as tumor cells and CNS-resident cells, such as neurons and astrocytes (Fig. 5B and Supplementary Fig. 6A). Analysis of cell proportions across treatment groups revealed an increased representation of T cells, particularly CD8^+^ T cells, in the combination therapy group (Fig. 5C, D, and Supplementary Fig. 6B).

GO enrichment analysis of DEGs in CD8^+^ T cells, comparing combination therapy with isotype control or anti-PD-1 monotherapy, revealed upregulation of pathways associated with interferon responses, T cell activation, and cytotoxicity (Fig. 5E and Supplementary Fig. 6C), corroborating a phenotypic shift toward an enhanced effector state. DEGs in CD8^+^ T cells between combination therapy and isotype control confirmed the upregulation of genes, such as *B2m* (suggesting activation of interferon-γ signaling) and *Gzmk* (indicating cytotoxic and effector-memory-like features) (Fig. 5F).

We next subset T cells and performed reclustering (Fig. 5G). Clear clustering was observed based on known T cell markers (Supplementary Fig. 6D, E), with a relative enrichment of effector CD8^+^ T-cell states, particularly CD8^+^ effector memory (Tem) cells, in anti-PD-1 containing conditions (Fig. 5H, I, and Supplementary Fig. 6F). Analysis of known marker genes for progenitor-exhausted (Tpe) and terminally exhausted (Tte) cells revealed a gradual transition from Tpe to Tte signatures (Supplementary Fig. 6G–J). Combination therapy showed a trend of higher proportion of CD8^+^ T cells exhibiting Tpe-like features.

For CD4^+^ T cells, GO pathway analysis revealed significant upregulation of pathways related to T cell activation, antigen processing, and presentation in the combination therapy group compared with controls (Supplementary Fig. 6K). CD4^+^ T cells were classified into Th1-like, Th2-like, and Tfh clusters using marker genes, such as *Tbx21*, *Igfbp7*, and *Bcl6* (Supplementary Fig. 6L, M). Tfh cells exhibited high Tfh scores consistent with previous studies^30,31^ (Supplementary Fig. 6M, N). Analysis of Tfh proportions showed that anti-PD-1 monotherapy was associated with a relative reduction of Tfh cells, whereas the addition of anti-CTLA-4 was associated with a trend of higher Tfh-like population (Supplementary Fig. 6O–Q).

We further applied ProjecTILs^32^, a reference-based projection tool, to map T cells to established functional states (Supplementary Fig. 6R). This analysis further supported a treatment-driven shift in T cell phenotypes. Combination therapy markedly increased the proportion of CD8^+^ T cells resembling effector memory (Tem) phenotypes compared with isotype control (Supplementary Fig. 6R, S) and also increased the proportion of CD4^+^ T cells resembling Tfh phenotypes (Supplementary Fig. 6U, V).

In summary, scRNA-seq of pooled BrM-bearing brain immune cells suggests that dual PD-1/CTLA-4 blockade reshapes the BrM immune microenvironment by increasing the proportion of T cells, including CD8^+^ T cells, and by shifting T-cell states in a complementary manner: PD-1 blockade is associated with enrichment of CD8^+^ effector-memory-like programs consistent with enhanced CTL functionality, whereas CTLA-4 blockade is associated with increased Tfh-like representation within the CD4^+^ compartment, indicative of broader immune modulation.

### Combination therapy increases CTL infiltration and functionality in BrMs

**Fig. 6:**
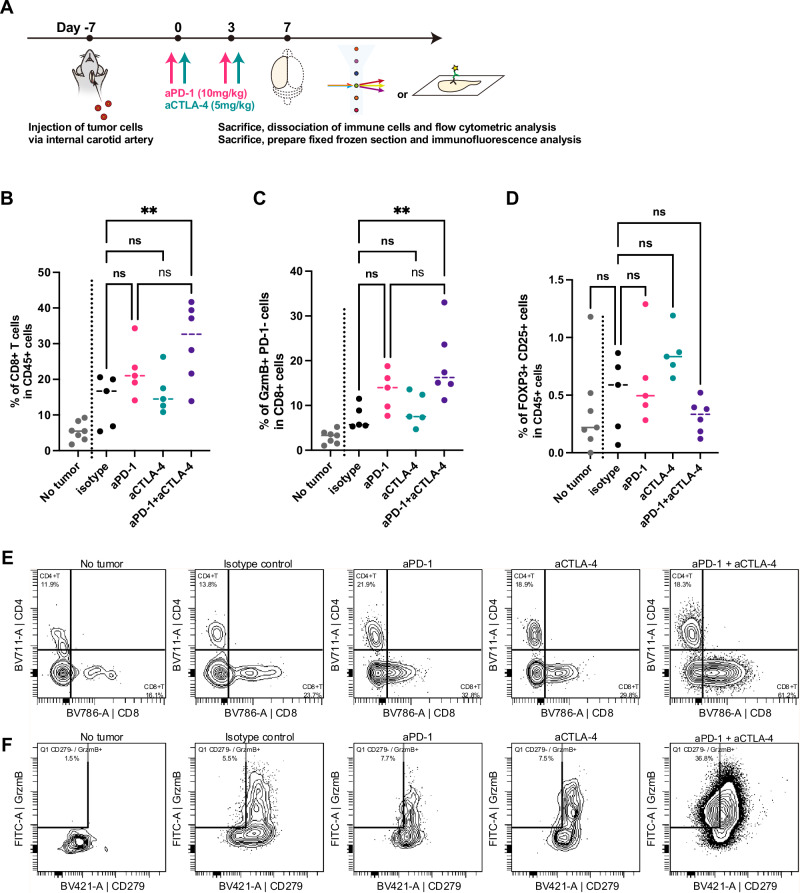

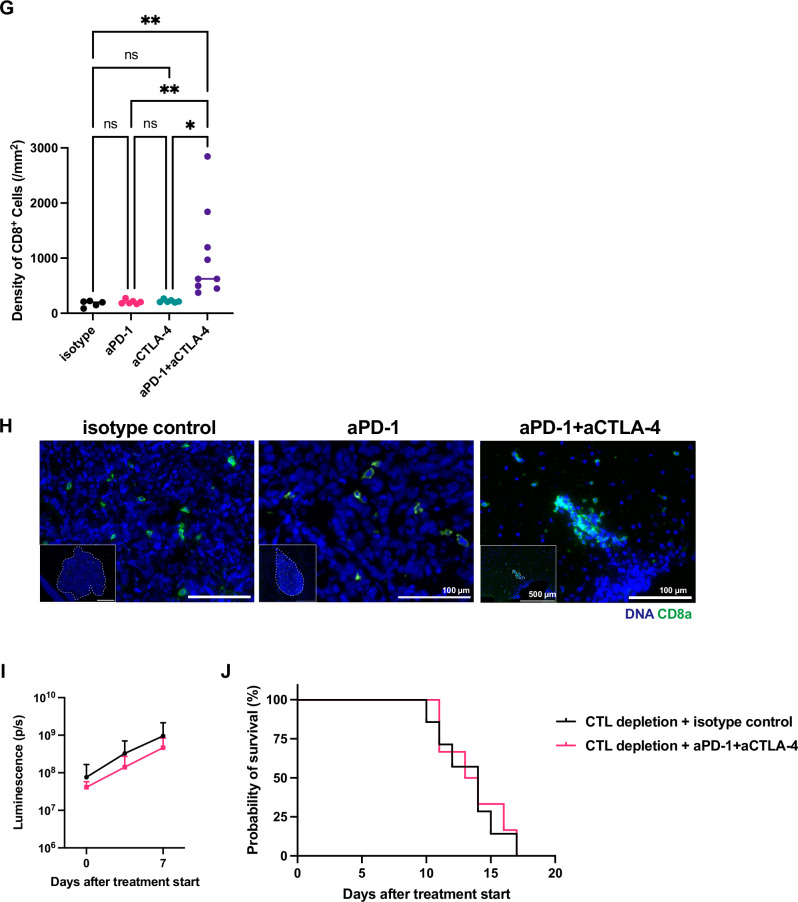
Anti-PD-1 plus anti-CTLA-4 combination therapy enhances CTL infiltration and function and is essential for its therapeutic effect in BrM. **A** Experimental design for flow cytometry and immunofluorescence analysis of BrM tissues. **B** Flow cytometry analysis showing an increased percentage of CD8^+^ T cells among CD45^+^ cells in BrM following combination therapy. Each dot represents data from individual mice (mean ± SD; no tumor, *n* = 7; isotype, *n* = 5; aPD-1, *n* = 5; aCTLA-4, *n* = 5; aPD-1 + aCTLA-4, *n* = 6). Each dot represents one mouse. Data are combined from 3 independent experiments. Statistical significance was assessed using one-way ANOVA followed by Tukey’s multiple-comparison. Pairwise *P* values were as follows: isotype vs. aPD-1, *P* = 0.2878; isotype vs. aCTLA-4, *P* = 0.9757; isotype vs. aPD-1 + aCTLA-4, *P* = 0.0047; aPD-1 vs. aPD-1 + aCTLA-4, *P* = 0.3024. Source data are provided as a Source data file. **C** Flow cytometry analysis showing an increased percentage of functional GzmB^+^PD-1^–^ cells within the CD8^+^ T cell population after combination therapy. Each dot represents data from individual mice (mean ± SD; no tumor, *n* = 7; isotype, *n* = 5; aPD-1, *n* = 5; aCTLA-4, *n* = 5; aPD-1 + aCTLA-4, *n* = 6). Each dot represents one mouse. Data are combined from 3 independent experiments. Statistical significance was assessed using one-way ANOVA followed by Tukey’s multiple-comparison. Pairwise *P* values were as follows: isotype vs. aPD-1, *P* = 0.1329; isotype vs. aCTLA-4, *P* = 0.5628; isotype vs. aPD-1 + aCTLA-4, *P* = 0.0014; aPD-1 vs. aPD-1 + aCTLA-4, *P* = 0.1329. Source data are provided as a Source data file. **D** Flow cytometry analysis of Treg cell percentage in CD4^+^ T cells from CMT167-derived BrM, showing no significant change across treatment groups (mean ± SD; no tumor, *n* = 7; isotype, *n* = 5; aPD-1, *n* = 5; aCTLA-4, *n* = 5; aPD-1 + aCTLA-4, *n* = 6). Each dot represents one mouse. Data are combined from 3 independent experiments. Statistical significance was assessed using one-way ANOVA followed by Tukey’s multiple-comparison. Source data are provided as a Source data file. **E** Representative scatter plot showing the gating of CD4^+^ and CD8^+^ T cells from the parent CD45^+^CD3^+^ population. **F** Representative scatter plot showing the gating of functional (Granzyme B^+^, PD-1^−^) cytotoxic T cells from the parent CD8^+^ T cell population. **G** Quantification from immunofluorescence showing a significantly higher density of infiltrating CD8^+^ T cells in BrM from the combination therapy group (mean ± SD; isotype, *n* = 5; aPD-1, *n* = 6; aCTLA-4, *n* = 6; aPD-1 + aCTLA-4, *n* = 9). Each dot represents one mouse (mean per mouse). Quantification was performed using 3 sections per mouse and tumor fields per section. Data are combined from 4 independent experiments. Statistical significance was assessed using Kruskal–Wallis followed by Dunn’s test. Pairwise *P* values were as follows: isotype vs. aPD-1, *P* > 0.9999; isotype vs. aCTLA-4, *P* > 0.9999; isotype vs. aPD-1 + aCTLA-4, *P* = 0.0026; aPD-1 vs. aCTLA-4, *P* > 0.9999; aPD-1 vs. aPD-1 + aCTLA-4, *P* = 0.0042; aCTLA-4 vs. aPD-1 + aCTLA-4, *P* = 0.0488. Source data are provided as a Source data file. **H** Representative immunofluorescence images of BrM showing increased CTL infiltration in the combination therapy group. **I** Luminescence analysis from CTL depletion experiments showing that the antitumor effect of combination therapy was abolished with CD8^+^ T cell depletion (mean ± SD; CTL depletion + isotype, *n* = 7; CTL depletion + aPD-1  + aCTLA-4, *n* = 6). Data are combined from 3 independent experiments. Source data are provided as a Source data file. **J** Kaplan–Meier survival curves from CTL depletion experiments demonstrating that the survival benefit of combination therapy was also abolished with CD8^+^ T cell depletion (mean ± SD; CTL depletion + isotype, *n* = 7; CTL depletion + aPD-1 + aCTLA-4, *n* = 6). Data are combined from 3 independent experiments. Source data are provided as a Source data file. BrM brain metastasis, GzmB Granzyme B, CTL cytotoxic T lymphocyte. ns not significant; **P < 0.01; ***P < 0.001.

To validate scRNA-seq findings at the protein level and assess functional changes, we performed flow cytometry and immunofluorescence analyses of BrM brain tissues (Fig. 6A). Flow cytometry confirmed a statistically significant increase in the percentage of CD8^+^ T cells among CD45^+^ immune cells in the BrM microenvironment following combination therapy compared with isotype control (Fig. 6B, E, and Supplementary Fig. 7A). In contrast, while CD8^+^ T-cell frequencies tended to be higher with combination therapy than with anti-PD-1 monotherapy, this comparison did not consistently reach statistical significance, reflecting inter-animal variability in the flow cytometry datasets. Immunofluorescence further confirmed these findings, showing a significantly higher density of CD8a^+^ cells infiltrating tumor tissue in the combination therapy group (Fig. 6G, H).

Functionally, flow cytometry demonstrated that combination therapy significantly increased the proportion of PD-1-negative and Granzyme B (GzmB)-positive cells within the CD8^+^ T cell compartment, indicating enhanced cytotoxic potential, compared with isotype control (Fig. 6C, F). Treg (Foxp3^+^CD25^+^) proportions remained low overall and did not significantly change with treatment (Fig. 6D and Supplementary Fig. 7B). Together, these results highlight a pronounced shift toward a more potent antitumor immune response mediated by CTLs following combination therapy. The findings were confirmed in experiments using LLC cell lines (Supplementary Fig. 7C–G).

Collectively, these data confirmed the infiltration and functional change toward anti-tumor phenotype of CTLs within BrM treated with anti-PD-1 plus anti-CTLA-4 combination therapy, with the most consistent differences observed relative to isotype control.

### CTLs are essential for the therapeutic efficacy of anti-PD-1 plus anti-CTLA-4 combination therapy in BrM

Given the observed increase in CTL infiltration and activation with combination therapy, we investigated whether CTLs are essential for treatment efficacy. We performed CD8^+^ T cell depletion using an anti-CD8a antibody concurrently with immunotherapy in the BrM model (Supplementary Fig. 8A). Successful depletion of CD8^+^ T cells in both peripheral blood and brain was confirmed (Supplementary Fig. 8B). The antitumor effect of combination therapy was completely abolished upon CD8^+^ T cell depletion, as shown by tumor growth assessed via bioluminescence (Fig. 6I) and survival analysis (Fig. 6J). Outcomes were comparable to those in the CTL-depleted isotype control group. Body weight changes were similar across groups (Supplementary Fig. 8D). These findings were reproduced in experiments using LLC cells (Supplementary Fig. 8E–H).

These results demonstrate that CD8^+^ T cells are indispensable mediators of the therapeutic benefits of anti-PD-1 plus anti-CTLA-4 immunotherapy in the NSCLC BrM model.

### Combination therapy promotes Tfh cell expansion and formation of TLS-like structures in the BrM microenvironment

Recalling our findings in human samples, where BrMs exhibited reduced TLSs and diminished *CCL19*/*CCL21* expression, we investigated whether combination therapy could induce TLS formation in our mouse model. First, scRNA-seq analysis of the mouse BrM model established by internal carotid artery injection did not reveal an increase in B cells, major components of TLSs, following anti-PD-1 plus anti-CTLA-4 treatment. However, there was a clear increase in Tfh-like CD4^+^ T cells, particularly in groups receiving anti-CTLA-4 (combination therapy and anti-CTLA-4 monotherapy) (Supplementary Fig. 6K–O). Tfh cells are well-established orchestrators of germinal center reactions and TLS formation^33^.

**Fig. 7:**
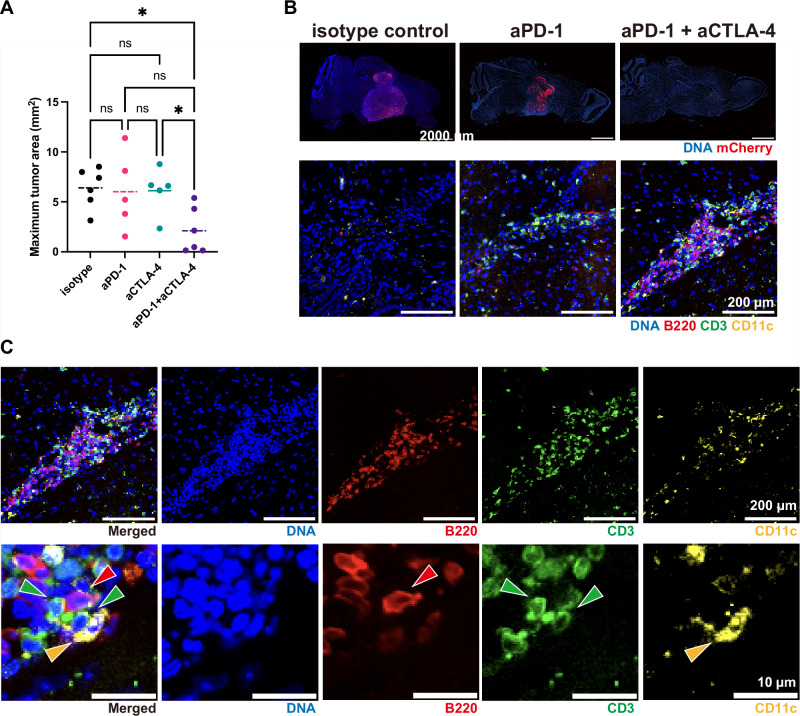
Combination therapy promotes the formation of tertiary lymphoid structure-like aggregates in responding BrM. **A** Quantification of maximum tumor area in the BrM of each treatment group, showing a significant reduction in tumor size with combination therapy compared with the isotype control (mean ± SD; isotype, *n* = 6; aPD-1, *n* = 5; aCTLA-4, *n* = 5; aPD-1 + aCTLA-4, *n* = 6). Each dot represents one mouse. Data are combined from 3 independent experiments. Statistical significance was assessed using Kruskal–Wallis followed by Dunn’s test. Pairwise *P* values were as follows: isotype vs. aPD-1, *P* = 0.6289; isotype vs. aCTLA-4, *P* = 0.9392; isotype vs. aPD-1 + aCTLA-4, *P* = 0.0145; aPD-1 vs. aCTLA-4, *P* = 0.6968; aPD-1 vs. aPD-1 + aCTLA-4, *P* = 0.0646; aCTLA-4 vs. aPD-1 + aCTLA-4, *P* = 0.0241. Source data are provided as a Source data file. **B** Representative IF images of BrM from each treatment group. Combination therapy resulted in smaller tumors (mCherry, red) and was associated with the formation of dense immune cell aggregates (bottom row) containing B cells (B220), T cells (CD3), and dendritic cells (CD11c). Images are representative of *n* = 6 mice per group. **C** High-resolution multiplex IF images from a mouse treated with combination therapy, showing a TLS-like aggregate composed of co-localized B cells (B220, red), T cells (CD3, green), and dendritic cells (CD11c, yellow). The bottom row provides a magnified view. Red, green and yellow arrowheads indicate representative B220^+^ B cells, CD3^+^ T cells and CD11c^+^ dendritic cells, respectively, within the TLS-like structure. Scale bars: 200 µm (top row), 10 µm (bottom row). Images are representative of *n* = 6 mice per group. BrM brain metastasis, IF immunofluorescence, TLS tertiary lymphoid structure. ns not significant; *P < 0.05.

To explore this further, we used a BrM model established by direct intracranial injection of tumor cells, combined with simultaneous subcutaneous tumor implantation, which allowed evaluation of bulk BrM tissue. Using this model, we found that no mice responded to isotype control antibody or anti-PD-1 monotherapy; however, a subset of mice responded to anti-PD-1 plus anti-CTLA-4 combination therapy (Fig. 7A). Consistent with this, combination therapy prolonged survival compared with isotype control (Supplementary Fig. 9A). No significant body weight loss was observed during the treatment course (Supplementary Fig. 9B). These findings were reproduced in an independent intracranial implantation model using LLC cells, showing similar effects on intracranial tumor burden, survival, and body weight (Supplementary Fig. 9C–E).

Immunofluorescence staining of key TLS components (CD3 for T cells, B220 for B cells, and CD11c for dendritic cells) revealed distinct immune cell clusters resembling TLSs in BrMs of combination therapy responders. These structures were characterized by aggregates of B and T cells located near the meninges or perivascular spaces adjacent to areas of reduced tumor burden (Fig. 7A, B). Such TLS-like structures were absent in both the isotype control and anti-PD-1 monotherapy groups. These findings suggest that anti-PD-1 plus anti-CTLA-4 combination therapy not only enhances CTL infiltration and function but may also promote the development of TLS-like structures within the BrM microenvironment, potentially contributing to a more robust and sustained antitumor immune response.

## Discussion

BrMs represent a major clinical challenge in NSCLC, often associated with poor prognosis and limited responses to conventional therapies, including anti-PD-1 monotherapy^5^. This study provides multifaceted evidence, combining clinical outcome analyses with preclinical murine models, that elucidates the immunosuppressive nature of the NSCLC BrM microenvironment and highlights the potential of anti-PD-1 plus anti-CTLA-4 combination immunotherapy to overcome these limitations.

Our clinical analysis revealed that NSCLC patients with baseline BrM experienced significantly poorer PFS with nivolumab monotherapy than those without BrM. In contrast, no significant difference in PFS was observed between patients with or without BrM treated with nivolumab plus ipilimumab, suggesting improved efficacy of the combination. Moreover, combination therapy was associated with a reduced incidence of new-onset BrMs. Although direct comparative trials are lacking in NSCLC, these findings align with clinical trial results in melanoma, where PD-1/CTLA-4 blockade demonstrated superior outcomes in BrM compared with PD-1 monotherapy^6,7^. Our observations are also consistent with recent NSCLC studies suggesting that combination regimens incorporating ipilimumab provide enhanced intracranial control^8,9^. In exploratory direct regimen comparisons within the baseline BrM population, OS was shorter in the nivolumab plus ipilimumab cohort than in the nivolumab cohort; however, this difference was attenuated after excluding patients with driver oncogene alterations (e.g., *EGFR* mutations), suggesting that oncogenic drivers may influence immunotherapy outcomes. Importantly, these analyses were retrospective and observational. Moreover, the nivolumab and nivolumab plus ipilimumab cohorts differed substantially in clinical context. In particular, nivolumab plus ipilimumab was predominantly administered for patients with PD-L1-negative tumors, a population generally associated with poorer immunotherapy outcomes, whereas nivolumab monotherapy was used regardless of PD-L1 status. Therefore, regimen-to-regimen comparisons should be interpreted cautiously, as confounding by indication and residual confounding may have influenced these outcomes.

To understand the underlying biology, we investigated the BrM immune microenvironment. Transcriptomic analyses of public datasets, together with immunohistochemical staining of our clinical specimens, consistently demonstrated that NSCLC BrMs harbor an immunosuppressive microenvironment compared with paired primary tumors. This was characterized by significantly reduced infiltration of CD8^+^ CTLs and Tregs, consistent with prior limited reports^12,14,15^. Importantly, high CTL density in resected BrMs was associated with improved post-resection survival, underscoring the prognostic relevance of CTLs even in this immune “cold” environment. In addition, we observed marked reductions in TLS density and downregulation of TLS-associated gene sets and chemokines, including *CCL19* and *CCL21*, in BrMs. Although TLSs were scarce, their presence correlated with higher CTL infiltration, suggesting that when present, TLSs can function as immune recruitment sites in BrMs, similar to their roles in primary tumors^11,27^. However, TLS presence in BrMs was not significantly associated with post-resection survival in our cohort, which may reflect limited statistical power due to the small number of TLS-positive cases and the inherent limitations of section-based TLS assessment in surgically resected CNS specimens. Together, the scarcity of CTLs and TLSs may help explain the limited efficacy of PD-1 monotherapy in BrM.

Our preclinical BrM model, established via internal carotid artery injection to mimic hematogenous spread, reproduced these clinical findings. Baseline BrMs exhibited lower CTL infiltration than primary lung tumors. Consistent with clinical data, anti-PD-1 monotherapy showed minimal efficacy against established BrMs, whereas anti-PD-1 plus anti-CTLA-4 combination therapy significantly inhibited tumor growth and prolonged survival. By contrast, in primary lung tumor models, both monotherapies demonstrated activity. These results suggest that CTLA-4 blockade is critical for overcoming the immune-excluded BrM microenvironment. Mechanistically, combination therapy increased CTL infiltration into BrMs, as confirmed by scRNA-seq, flow cytometry, and immunofluorescence. Furthermore, scRNA-seq and flow cytometry revealed that infiltrating CTLs acquired an enhanced effector phenotype, marked by interferon-response pathway activation and GzmB expression. Critically, CD8^+^ T cell depletion abrogated therapeutic benefit, confirming that CTLs are indispensable for the efficacy of combination therapy. These results align with melanoma BrM studies showing that CTLA-4 blockade enhances T cell trafficking and activity^17,19^, and extend these findings directly to NSCLC BrM.

Intriguingly, scRNA-seq revealed expansion of T_fh_-like cells following combination therapy, particularly with anti-CTLA-4 treatment. T_fh_ cells play a pivotal role in B cell activation and TLS development and are known to expand in response to CTLA-4 blockade^34^. Subsequent immunofluorescence analysis of responsive mice demonstrated TLS-like aggregates containing B and T cells, typically located near the meninges. Although the functional maturity of these TLS-like structures remains to be established, their induction by combination therapy suggests a potential mechanism for amplifying the local antitumor immune response within BrMs. This observation is consistent with emerging evidence of TLS formation in brain tumors, including glioblastoma, in both preclinical models and human samples^35,36^. Such induction of TLSs may provide sustained immune surveillance and long-term local immune activity.

This study has several strengths, including the integration of retrospective clinical outcome data, paired human tissue analysis, and mechanistic investigations using a clinically relevant BrM model. Advanced techniques, such as scRNA-seq and functional depletion studies, further strengthened the findings. However, several limitations should be noted. The clinical analyses were retrospective, with inherent biases and relatively small sample sizes, particularly for the anti-PD-1 plus anti-CTLA-4 combination therapy group, owing to the later approval of the regimen and the limited number of eligible patients. The preclinical experiments relied on specific murine cell lines (CMT167 and LLC), which may not fully reflect the heterogeneity of human NSCLC BrMs. Another limitation is that the ICA model consistently induced meningeal involvement, which may resemble aspects of leptomeningeal disease in addition to parenchymal brain metastasis. However, histological quantification indicated that combination therapy reduced both parenchymal lesions and meningeal tumor burden in this model. Moreover, we complemented these findings using an additional intracranial implantation model, which supported the therapeutic activity of dual PD-1/CTLA-4 blockade, thereby increasing the generalizability of our conclusions across model systems. In the CMT167 ICA model, the survival difference between dual therapy and anti-PD-1 monotherapy did not reach statistical significance, whereas this comparison was significant in the LLC model. This variability may reflect model-specific tumor kinetics and/or limited power for detecting differences between active treatment arms. Although TLS-like structures were observed in the intracranial injection model, their detailed functional characterization requires further investigation. In addition, the precise mechanisms by which CTLA-4 blockade enhances CTL trafficking and potentially induces TLS formation in the CNS (e.g., through modulation of peripheral T-cell priming versus direct effects on CNS vasculature or resident cells) require further investigation.

In conclusion, our study demonstrates that NSCLC BrMs possess a distinct immunosuppressive microenvironment characterized by low CTL and TLS densities, which contributes to the limited efficacy of anti-PD-1 monotherapy. The combination of anti-PD-1 and anti-CTLA-4 immunotherapy effectively overcame this resistance in preclinical models, with efficacy critically dependent on enhancing CD8^+^ T cell infiltration and function. This regimen also showed potential to induce TLS-like structures. Collectively, these findings provide a rationale for further evaluation of combination immunotherapy strategies, particularly anti-PD-1 plus anti-CTLA-4 blockade, in NSCLC patients with BrM. Prospective clinical trials are warranted. Future studies should focus on dissecting the mechanisms of immune cell trafficking into BrM and exploring strategies to enhance local immune responses, potentially through TLS induction or modulation of the unique BrM microenvironment. In addition, it will be important to evaluate other immunotherapy agents and rational combinations beyond PD-1/CTLA-4 blockade, including approaches specifically designed to promote TLS formation/maturation, as potential means to convert immune-excluded BrMs into more immunologically permissive lesions.

## Methods

### Collection of clinical information and specimens

The study protocol was prepared in accordance with the Declaration of Helsinki and approved by the Kyoto University Graduate School and Faculty of Medicine Ethics Committee (Kyoto, Japan; certification numbers: R2163 and R2860). Clinical information was retrospectively obtained from electronic medical records, and archived clinical specimens collected with comprehensive consent at the time of collection were used. Patients were given the opportunity to opt out in accordance with Japanese guidelines.

### Survival analysis of patients

For the analysis of treatment outcomes in patients who received ICIs, we retrospectively enrolled patients with NSCLC who had received nivolumab (as second- or later-line palliative treatment between January 2016 and December 2017, as previously reported^37^) or nivolumab plus ipilimumab (with or without cytotoxic chemotherapy, regardless of treatment line, between January 2021 and March 2024). Patients with a history of multiple cancers or without radiographically measurable lesions were excluded. The index date was defined as the date of ICI initiation. Tumor response was evaluated according to Response Evaluation Criteria in Solid Tumors version 1.1 (RECIST v1.1) for overall response, and intracranial response was assessed on contrast-enhanced or non-contrast brain MRI according to RECIST v1.1, using up to two intracranial target lesions. To reduce potential confounding of intracranial response assessment by recent local therapy, patients who received brain-directed radiotherapy within 30 days prior to the index date were excluded from the response analyses. These patients were included in time-to-event analyses, including PFS and OS. Treatment-related adverse events were graded according to the Common Terminology Criteria for Adverse Events version 5.0 (CTCAE v5.0). Patients were stratified by the presence or absence of baseline BrMs, as assessed through medical records and radiological findings. Baseline BrM was defined as the presence of radiographically confirmed brain metastases on imaging between the diagnosis of NSCLC and the start of ICI therapy. PFS was defined as the time from ICI initiation to disease progression or death from any cause. Patients without progression or death were censored at their last follow-up visit. Overall survival (OS) was defined as the time from ICI initiation to death from any cause. Patients who survived were censored at the last follow-up visit. The data cutoff was July 31, 2024.

For the analysis of the cumulative incidence of BrMs, we included only patients without baseline BrMs from the above cohort. Cumulative incidence was estimated using the Kaplan–Meier method. The occurrence of BrMs was considered an event regardless of progression at other sites, and patients who died without BrMs were censored at the date of death, as described previously^38^. The data cut-off was July 31, 2024.

In the analysis of survival outcomes according to immune cell density in BrM, we retrospectively enrolled NSCLC patients with resected BrM between February 1, 2008, and December 31, 2021^39^. As described in the immunohistochemical analysis of the BrM tissue microarray (TMA) section, we quantified the density of immune cells (CD8a^+^ immune cells as CTLs and FOXP3^+^ immune cells as Tregs). The densities were dichotomized into high- and low-infiltration groups using the median value as the cutoff point. Associations of the variables with OS were evaluated using Cox proportional hazards regression in both univariable and multivariable models. As the primary approach, CTL density was analyzed as a dichotomized variable (high vs. low, median cutoff). Univariable Cox models included age (≥75 vs. <75), sex, histology (squamous vs. non-squamous), driver alteration status, preoperative steroid use, completeness of BrM resection, and CTL infiltration group, Treg infiltration group. The multivariable Cox model included driver alteration status, preoperative steroid use, completeness of resection, and CTL infiltration group, based on clinical relevance and to limit model complexity given the sample size. As a sensitivity analysis, CTL density was additionally modeled as a continuous variable after log2 transformation (log2[CD8 density + 1]), and the same univariable and multivariable analyses were repeated. Survival analysis was performed using the Kaplan–Meier method. OS was defined as the time from BrM resection to death from any cause. Patients who survived were censored at the last follow-up visit. The data cutoff was July 31, 2024.

### Public transcriptome analysis of paired specimens

Publicly available transcriptome datasets of paired primary NSCLC and BrM samples were analyzed. Data from GSE161116^22^ and the NSCLC subset of GSE248830^23^ were obtained from the Gene Expression Omnibus database. Raw data were normalized using quantile normalization and log2 transformation. Differential gene expression analysis between primary tumors and BrMs was performed using the *limma* package^40^, accounting for the paired sample design.

For principal component analysis (PCA) visualization, batch effects from patients were removed with the *removeBatchEffect* function, specifying tissue (primary vs. BrM) as the variable of interest to retain. Volcano plots were generated to visualize differentially expressed genes. Gene set variation analysis (GSVA) was performed using the *GSVA* package^41^, utilizing immune-related and MSigDB gene sets. Additionally, previously reported gene sets associated with TLS formation in NSCLC tissue were included in the GSVA analysis^28,29^ (Supplementary Data 2). For GSVA analyses, a fixed random seed was used to ensure reproducibility of the results and figures. Cell type deconvolution was performed using the immunedeconv package^42^ and the consensusTME algorithm^24^.

### Immunohistochemical analysis of clinical specimens

We used archived formalin-fixed, paraffin-embedded (FFPE) tissue blocks of NSCLC BrM surgically removed between February 1, 2008, and December 31, 2021, along with corresponding paired lung primary specimens collected between February 1, 2003, and December 31, 2021. For most BrM samples, TMAs consisting of two 2 mm cores per sample were created. TMA preparation was performed after expert pathological review confirmed the NSCLC BrM diagnosis. Immunohistochemical staining was conducted on 4 μm sections from either whole FFPE blocks or TMAs.

Immunostaining was performed using the antibodies listed in Supplementary Data 1. Sections were incubated with horseradish peroxidase-conjugated secondary antibodies and visualized with 3,3′-diaminobenzidine substrates. Hematoxylin was used as a counterstain. The stained sections were digitally scanned and analyzed using QuPath version 0.5.0. Immune cells were classified using a pixel classifier (random trees) in QuPath. CD8^+^ or FOXP3^+^ immune cells were identified as CTLs and Tregs, respectively. The tumor tissue area was defined using H&E staining and used to calculate immune cell density (cells/mm^2^).

### Evaluation of TLS formation in clinical specimens

TLS presence and density were assessed by consensus between two pathologists using hematoxylin and eosin (H&E)-stained sections. TLSs were defined as well-circumscribed, round-to-oval cellular aggregates composed mainly of lymphocytes and/or plasma cells. TLSs were counted within the tumor and in the surrounding region up to 1 mm from the tumor border. Lymphoid aggregates within normal lung tissue and separated from the tumor by intact parenchyma were excluded. TLS density was calculated as the number of TLSs per square millimeter of tumor area. Quantification of TLS formation based on H&E morphology was performed according to previously established criteria in the literature^43,44^. To further confirm TLS identity, representative TLS-positive primary tumors and BrMs were additionally evaluated by immunohistochemistry for CD3, CD20, CD21, PNAd, BCL6, and CCL21.

### Mice

Six-week-old male B6J (C57BL/6J; Jackson Laboratory Japan, Inc., Yokohama, Japan; JAX stock no. 000664) and B6 Albino (B6N-*Tyr*^*c-Brd*^/BrdCrCrl; Jackson Laboratory Japan, Inc., formerly Charles River Laboratories Japan, Yokohama, Japan) were purchased and used in this study. The B6 Albino strain was identified by the supplier strain name B6N-Tyrc-Brd/BrdCrCrl. No noncommercial animal strains were used in this study. After acclimatization, the mice were used for experiments.

Mice were maintained under specific pathogen-free (SPF) conditions in a barrier facility at Kyoto University. Mice were housed under a 12-h light/dark cycle at controlled ambient temperature and humidity. Experimental and control animals were housed under the same conditions and were not bred separately. Where feasible, mice from different treatment groups were allocated from the same cages. Animals were randomly assigned to treatment groups. Investigators were blinded to group allocation during data collection and analysis, where applicable.

For tumor implantation, mice were anesthetized with a combination of midazolam, butorphanol, and medetomidine, and then recovered with atipamezole. For in vivo luminescence imaging, daily weight checks, or drug infusion, anesthesia was maintained with isoflurane.

For intracranial and lung tumor models, tumor size could not be measured externally. Tumor burden was therefore monitored by bioluminescence imaging at the indicated time points and by daily assessment of body weight, neurological symptoms, and general condition. Mice were euthanized if they lost more than 20% of their baseline body weight, developed neurological symptoms, showed severe distress, impaired mobility, or a moribund appearance. For subcutaneous tumors, tumor size was measured with calipers every 2–3 days, and the maximum tumor size permitted by the institutional animal protocol was 800 mm^3^. This limit was not exceeded in any experiment. Euthanasia was performed by cervical dislocation under deep anesthesia with medetomidine, midazolam, and butorphanol, or isoflurane, according to the approved animal protocol. All experiments were conducted in accordance with the humane endpoint criteria and maximum tumor burden limits approved by the Animal Research Committee of Kyoto University Graduate School of Medicine, and these limits were not exceeded. All animal experiments were approved by the Animal Research Committee of Kyoto University (ID: MedKyo19594, Medkyo20258, Medkyo21274, Medkyo22254, Medkyo23211, and Medkyo24244). All animal experiments were conducted in accordance with the approved protocols and ARRIVE guidelines.

### Cell lines and fluorescence/luminescence tagging

CMT167 cells were purchased from ECACC (European Collection of Authenticated Cell Cultures; catalog no. EC10032302-F0), and LLC1 cells were purchased from ATCC (American Type Culture Collection; catalog no. CRL-1642). Antibodies and key reagents used in this study, including suppliers, catalog numbers, clones, and dilutions where applicable, are listed in Supplementary Data 1. To generate fluorescence/luminescence-tagged cell lines, the Precise Integration into Target Chromosome (PITCh) protocol^45^ was used to insert the CAG-mCherry-T2A-Akaluc cassette into the mouse safe harbor locus ROSA26. Briefly, the CAG-mCherry-T2A-Akaluc construct flanked by microhomology and a PITCh-universal target sequence was generated. A CRISPR-Cas9 plasmid containing dual sgRNAs targeting both the PITCh-universal sequence and the ROSA26-specific target sequence was also constructed. These plasmids were transfected into CMT167 and LLC1 cells using Lipofectamine® 3000 (Thermo Fisher Scientific) following the manufacturer’s instructions. After one week to allow stable knock-in and expression of the tagged construct, tagged cells were selected by flow cytometry and cloned using the limiting dilution method.

The pcDNA3 Venus-Akaluc plasmid was provided by RIKEN BRC through the National BioResource Project of MEXT (cat. RDB15781)^46^. The pX330S-2 was a gift from Takashi Yamamoto (Addgene plasmid # 58778)^47^.

### Establishment of mouse BrMs (internal carotid artery injection)

Six-week-old male B6 Albino mice were used for BLI-based monitoring of the internal carotid artery injection BrM model. Six-week-old male B6J mice were used for tissue-based immunological analyses when indicated.

To mimic the natural metastatic pathway of human BrM while enabling accurate quantification of tumor burden and avoiding damage caused by direct intracranial injection, we employed an internal carotid artery (ICA) injection model by minor revision of previously reported methods^48,49^. In short, mice were anesthetized, the hair on the anterior neck was removed using a depilatory cream. Under clean conditions, an approximately 1-cm incision was made in the anterior neck, the left common carotid artery was exposed, and the left external carotid artery was ligated. A microcatheter was inserted into the left common carotid artery, and 1.0 × 10^6^ tumor cells suspended in 100–150 µL PBS were injected slowly into the left internal carotid artery over approximately 1 min. After injection, the left common carotid artery was ligated, and the wound was closed with VetBond Tissue Adhesive (3 M, St. Paul, MN, USA). Anesthesia was reversed with atipamezole. With an experienced operator, the procedure takes approximately 20 min per mouse. The success rate was approximately 90%, defined as recovery from anesthesia after injection, survival for 24 h, and suitable for subsequent experiments.

Treatment was initiated 7 days after injection (designated as day 0). To monitor tumor growth and lung metastasis, in vivo bioluminescence imaging (BLI) and body weight measurements were performed on days 3, 7, 14, and 21, with daily monitoring of general condition and neurological symptoms. A subset of mice was euthanized on day 7 for sample collection. Remaining mice were euthanized if they showed signs of distress, including >20% weight loss or neurological symptoms, as specified in the Mice section.

A known limitation of ICA delivery is that tumor cells can disseminate into the cerebrospinal fluid (CSF) space, resulting in meningeal involvement in addition to parenchymal lesions; therefore, histological assessment was performed to document the anatomical distribution of tumor growth.

### Establishment of mouse BrM (local injection plus subcutaneous injection)

To evaluate the histology of bulk BrM, we simultaneously established a subcutaneous tumor in the same mouse to induce an adequate immune reaction against BrM, following several previous studies^17–19^. In brief, 6-week-old male B6J mice were anesthetized with medetomidine, midazolam, and butorphanol as described above. For BrM induction, 1.0 × 10^5^ mCherry-Akaluc-tagged CMT167 or LLC cells suspended in a 2:1 mixture of serum-free RPMI1640 medium and Matrigel (Corning, Corning, NY, USA) were stereotactically injected into the brain parenchyma, as previously described^50^. The cell suspension (1.5 µL) was slowly injected over at least 30 s using a Hamilton syringe (Hamilton Company, Reno, NV, USA). Concurrently, 5.0 ×  10^5^ tumor cells prepared in the same serum-free medium/Matrigel suspension were injected subcutaneously into the right flank of the same mouse. Treatment was initiated 7 days after tumor cell injection (designated as day 0). Body weight, neurological symptoms, general condition, and subcutaneous tumor growth were monitored daily. Subcutaneous tumor size was measured with calipers every 2–3 days, and mice were euthanized before reaching the maximum tumor size permitted by the approved animal protocol. The mice were euthanized on day 7 of treatment for subsequent histological evaluation of BrMs.

### Establishment of mouse primary lung cancer (tail vein injection)

To establish a primary lung cancer model, tumor cells were injected into the tail vein. Six-week-old male B6J or B6 Albino mice were anesthetized with medetomidine, midazolam, and butorphanol as described above. Luminescence-tagged CMT167 or LLC cells (1.0 × 10^6^) suspended in 200 µL PBS were injected into the lateral tail vein using a 27 G needle. Treatment was initiated seven days after injection (day 0). To monitor tumor growth and metastatic burden in the lungs, in vivo BLI and body weight measurements were performed on days 3, 7, 14, and 21, with daily monitoring of general condition and respiratory or neurological symptoms. A subset of mice was euthanized on day 7 for sample collection. The remaining mice were euthanized as described previously.

### In vivo luminescence imaging

In vivo BLI was performed using an IVIS Lumina II system (Caliper Life Sciences, Hopkinton, MA, USA) on specific days for tumor monitoring. Mice were anesthetized with isoflurane as described above. To achieve stable and strong luminescent signals, TokeOni (FujiFilm Wako, Osaka, Japan) was suspended in PBS and administered intraperitoneally at 1 mg/kg. Ten minutes after injection, images were captured with an exposure time of 10 s. Regions of interest (ROIs) were manually drawn based on anatomical landmarks in the bright-field image, without visualizing the bioluminescent signal. ROIs were defined as circles with diameters of 1 cm for the head and 3 cm for the chest. Bioluminescence signals within the ROIs were quantified as photons/s using Living Image software (Caliper Life Sciences).

### Flow cytometry analysis

Mice were deeply anesthetized with medetomidine, midazolam, and butorphanol as described above and transcardially perfused with ice-cold PBS to remove circulating blood cells. The left cerebrum was harvested after perfusion, minced with a razor blade, and incubated in a dissociation buffer consisting of RPMI 1640 medium (Gibco, Thermo Fisher Scientific, Waltham, MA, USA), collagenase IV (1 mg/mL; Sigma-Aldrich, St. Louis, MO, USA), and DNase I (0.05 mg/mL; Sigma-Aldrich). Single-cell suspensions were obtained by enzymatic and mechanical dissociation using the GentleMACS dissociator (Miltenyi Biotec, Bergisch Gladbach, Germany). The enzymatic reaction was halted by adding EDTA solution. The cell suspension was filtered through a 70 µm cell strainer (Greiner Bio-One, Kremsmünster, Austria) and centrifuged. Debris was removed by density gradient centrifugation with 36% Percoll (GE Healthcare, Chicago, IL, USA). Red blood cells were lysed with RBC lysis buffer (Roche, Basel, Switzerland). Cells were stained for viability with FVS780 (BD Biosciences, Franklin Lakes, NJ, USA), followed by surface marker staining. After fixation and permeabilization with Foxp3/transcription factor fixation/permeabilization concentrate and diluent (eBioscience, San Diego, CA, USA), intracellular staining was performed. Antibodies are listed in Supplementary Data 1. Compensation was performed using CompBeads software (BioLegend). Flow cytometry was conducted on an LSRFortessa (BD Biosciences), and data were analyzed using OMIQ software (OMIQ, Inc., Santa Clara, CA, USA).

### Immunofluorescence analysis

Mice were deeply anesthetized with medetomidine, midazolam, and butorphanol as described above, and then transcardially perfused with PBS followed by fixation with 4% paraformaldehyde (PFA) in PBS. The left cerebrum was dissected and post-fixed in 1% PFA/PBS overnight at 4 °C. Tissues were cryoprotected overnight in 30% sucrose in PBS at 4 °C, embedded in optimal cutting temperature compound (Sakura Finetek, Torrance, CA, USA), and rapidly frozen in liquid nitrogen-cooled isopentane. Frozen sections (7 µm) were cut using a Leica CM3050S cryostat (Leica Biosystems, Wetzlar, Germany). Sections were washed with PBS and blocked with PBS containing 0.3% Triton X-100 (Sigma-Aldrich), 1% bovine serum albumin (BSA; Sigma-Aldrich), and 1% mouse Fc Block (BioLegend, San Diego, CA, USA). Immunostaining was performed using fluorophore-conjugated antibodies (Supplementary Data 1) diluted in blocking buffer. Nuclei were counterstained with Hoechst 33342 (Dojindo, Kumamoto, Japan). Sections were mounted with ProLong™ Gold Antifade Mountant (Invitrogen, Thermo Fisher Scientific, Waltham, MA, USA) and imaged with a BZ-X710 all-in-one fluorescence microscope (Keyence, Osaka, Japan).

To measure CTL density, CD8a-positive cells were manually counted in captured images. The area of mCherry-labeled tumor tissue was measured using Fiji software (ImageJ, NIH, Bethesda, MD, USA). CTL density was calculated as the number of CD8a-positive cells per square millimeter of the mCherry-positive tumor area.

The t-CyCIF method was used for multiplexing^51,52^. Briefly, sections underwent iterative cycles of staining, imaging, and signal bleaching. In each cycle, sections were incubated with primary antibodies directly conjugated to Alexa Fluor 488 or Alexa Fluor 647 (Supplementary Data 1). Nuclei were counterstained with Hoechst 33342. Sections were mounted with Fluoromount-G (Southern Biotech) for imaging with a BZ-X710 microscope (Keyence). After imaging, coverslips were gently removed by immersing slides in PBS. Fluorophores were inactivated by incubating sections in hydrogen peroxide (H_2_O_2_)^–^ and sodium hydroxide (NaOH)-based bleaching buffers under light for 30 min. This cycle of staining, imaging, and bleaching was repeated for all antibodies in the panel. Finally, images from all cycles were registered and aligned using the publicly available PystackReg library (version 0.2.8) in Python, using the Hoechst 33342 nuclear signal as a fiducial marker for accurate overlay of multiplexed images.

### Single-cell RNA sequencing (scRNA-seq) library preparation and sequencing

Single-cell suspensions were prepared from the left cerebrum of mice as described in the flow cytometry section. To obtain sufficient cells for single-cell RNA sequencing (scRNA-seq), suspensions from 3 to 4 mice were pooled for each treatment condition. Cells were fixed with 4% formaldehyde according to the manufacturer’s protocol (10× Genomics, Pleasanton, CA, USA). Libraries were generated using the 10× Genomics Chromium Single Cell Gene Expression Flex Kit (10× Genomics) following the manufacturer’s instructions. Library preparation and sequencing were performed at the Research Institute for Microbial Diseases, Osaka University (Suita, Osaka, Japan). Sequencing was conducted on an Illumina NovaSeq using 150 bp paired-end reads.

### Single-cell RNA sequencing (scRNA-seq) data analysis

Raw sequencing data were processed with the Cell Ranger pipeline (10× Genomics) to align reads to the mouse reference genome (mm10) and generate a gene-barcode matrix. Data analysis was performed using the Seurat R package (version 5.1.0).

Low-quality cells and doublets were removed before normalization and scaling, and highly variable genes were identified. Dimensionality reduction was performed using principal component analysis (PCA), followed by clustering and visualization with uniform manifold approximation and projection. Major cell types, including T cells, myeloid cells, and tumor cells, were identified and annotated based on canonical marker gene expression. T cells were subsequently re-clustered for higher-resolution analysis. DEGs were identified between clusters and treatment groups within the CTL cluster. To further characterize T cell states, we calculated signature scores for terminally exhausted T cells (Tte), progenitor exhausted T cells (Tpe), and T follicular helper (Tfh) cells using previously published gene sets^53^ and known marker genes of Tfh cell development^30,31^. Gene Ontology (GO) enrichment analysis was performed on DEGs to identify enriched pathways. For GO enrichment analyses, a fixed random seed was used to ensure reproducibility of the results and figures. Additionally, T cell states were classified using ProjecTILs^32^, a reference-based classification method.

### Statistical analysis

No formal statistical method was used to predetermine sample size. Sample sizes were chosen based on previous studies using similar mouse tumor models, preliminary experiments, and feasibility considerations, while ensuring sufficient biological replicates to assess reproducibility and treatment effects. For animal experiments, group sizes were selected to be comparable to those commonly used in preclinical immunotherapy studies and were sufficient to detect consistent treatment-associated differences across independent experiments. For clinical and public transcriptomic analyses, all eligible samples meeting the predefined inclusion criteria were included.

Statistical analyses were conducted using R software (version 4.4.2) and GraphPad Prism (version 10.4.2; GraphPad Software, Boston, MA, USA). Survival curves were estimated by the Kaplan–Meier method, with differences assessed using the log-rank test. Continuous variables were compared between two groups using Student’s *t* test or the Mann–Whitney *U* test, depending on data distribution. For multiple group comparisons, ANOVA or the Kruskal–Wallis test followed by appropriate post hoc tests was applied. Categorical data were analyzed using the chi-squared test or Fisher’s exact test. Statistical significance was defined as *P* < 0.05. The specific statistical tests used are detailed in the figure legends or Results section.

### Reporting summary

Further information on research design is available in the Nature Portfolio Reporting Summary linked to this article.

## Supplementary information

Supplementary Information

Description of Additional Supplementary Files

Supplementary Data 1–12

Reporting Summary

Transparent Peer Review File

## Source data

Source Data

## Data Availability

Source data are provided with this paper. Raw single-cell RNA sequencing data have been deposited in the DDBJ database [https://ddbj.nig.ac.jp/search/entry/gea/E-GEAD-1219], accession no: E-GEAD-1219. Raw FASTQ files are available from the DDBJ Sequence Read Archive (DRA) under accession numbers DRR909906–DRR909909, linked to GEA accession E-GEAD-1219. The raw microarray datasets used in this study are available from the Gene Expression Omnibus under accession codes GSE161116 and GSE248830. All other data generated in this study are provided in the Supplementary information, Supplementary data and Source data files. Source data are provided with this paper.

## Code availability

No custom code was developed in this study. All analyses were performed using publicly available software and packages described in the Methods section.
