# Supplementary material for: Anti-PD-1 plus anti-CTLA-4 blockade overcomes immune exclusion in NSCLC brain metastases by enhancing CD8+ T cell responses and promoting tertiary lymphoid structure formation
Source: Nat Commun. 2026 Jul 7;17:5681. doi: 10.1038/s41467-026-74782-7 (PMC13342341; doi:10.1038/s41467-026-74782-7)
Supplement: Supplementary file 1 — Supplementary Information [file 41467_2026_74782_MOESM1_ESM.pdf]

Supplementary Information

Anti-PD-1 Plus Anti-CTLA-4 Blockade Overcomes Immune Exclusion in NSCLC Brain Metastases by Enhancing CD8+ T Cell Responses and Promoting Tertiary Lymphoid Structure Formation

Kazutaka Hosoya et al.
First author: Kazutaka Hosoya
Corresponding author: Hiroaki Ozasa

This Supplementary Information PDF contains Supplementary Figures and their corresponding legends. Supplementary Tables are provided separately as Supplementary Data files, distinct from the Source Data file.

Supplementary Data 1. Antibodies and reagents used in the study
Supplementary Data 2. Gene sets used in the study
Supplementary Data 3. Patient Characteristics (Nivolumab)
Supplementary Data 4. Patient Characteristics (Nivolumab+Ipilimumab)
Supplementary Data 5. Efficacy of nivolumab monotherapy or nivolumab + ipilimumab combination therapy
Supplementary Data 6. Intracranial efficacy of nivolumab monotherapy or nivolumab + ipilimumab combination therapy
Supplementary Data 7. Adverse events of nivolumab monotherapy or nivolumab + ipilimumab combination therapy
Supplementary Data 8. Patient characteristics of those who have paired specimens of primary lesion and brain metastasis of NSCLC.
Supplementary Data 9. Characteristics of TMA cohort patients stratified by cytotoxic T lymphocytes density
Supplementary Data 10. Characteristics of TMA cohort patients stratified by Treg density
Supplementary Data 11. Univariable and multivariable Cox regression analyses for post-resection overall survival in the BrM TMA cohort (CTL infiltration dichotomized at the median).
Supplementary Data 12. Univariable and multivariable Cox regression analyses for post-resection overall survival in the BrM TMA cohort (CTL infiltration as log2-transformed continuous variable)

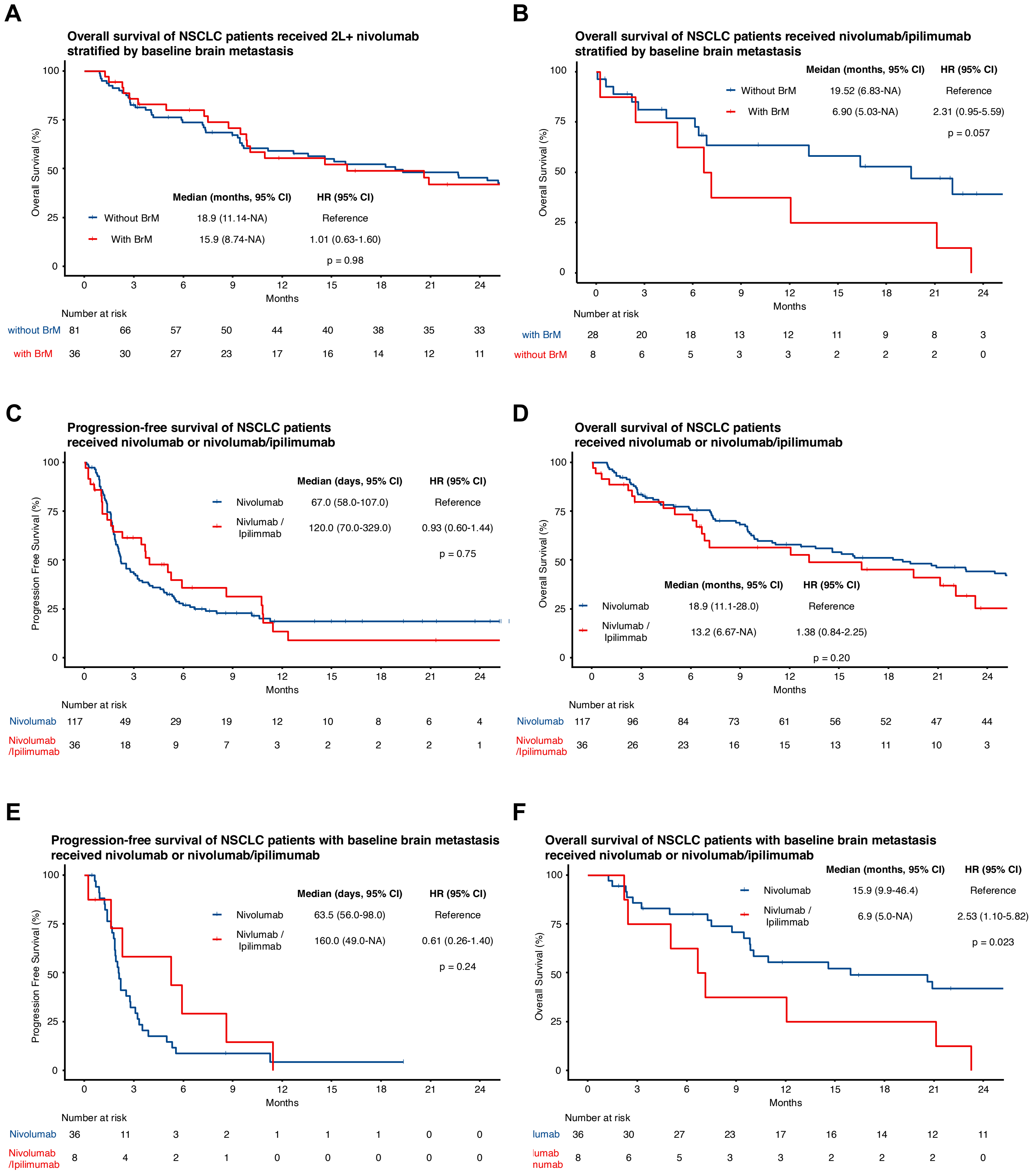
A
Overall survival of NSCLC patients received 2L+ nivolumab stratified by baseline brain metastasis
Overall Survival (%)
Median (months, 95% CI)
HR (95% CI)
Without BrM
18.9 (11.14-NA)
Reference
With BrM
15.9 (8.74-NA)
1.01 (0.63-1.60)
p = 0.98
Months
Number at risk
without BrM 81 66 57 50 44 40 38 35 33
with BrM 36 30 27 23 17 16 14 12 11
B
Overall survival of NSCLC patients received nivolumab/ipilimumab stratified by baseline brain metastasis
Overall Survival (%)
Meidan (months, 95% CI)
HR (95% CI)
Without BrM
19.52 (6.83-NA)
Reference
With BrM
6.90 (5.03-NA)
2.31 (0.95-5.59)
p = 0.057
Months
Number at risk
with BrM 28 20 18 13 12 11 9 8 3
without BrM 8 6 5 3 3 2 2 2 0
C
Progression-free survival of NSCLC patients received nivolumab or nivolumab/ipilimumab
Progression Free Survival (%)
Median (days, 95% CI)
HR (95% CI)
Nivolumab
67.0 (58.0-107.0)
Reference
Nivlumab / Ipilimmab
120.0 (70.0-329.0)
0.93 (0.60-1.44)
p = 0.75
Months
Number at risk
Nivolumab 117 49 29 19 12 10 8 6 4
Nivolumab /Ipilimumab 36 18 9 7 3 2 2 2 1
D
Overall survival of NSCLC patients received nivolumab or nivolumab/ipilimumab
Overall Survival (%)
Median (months, 95% CI)
HR (95% CI)
Nivolumab
18.9 (11.1-28.0)
Reference
Nivlumab / Ipilimmab
13.2 (6.67-NA)
1.38 (0.84-2.25)
p = 0.20
Months
Number at risk
Nivolumab 117 96 84 73 61 56 52 47 44
Nivolumab /Ipilimumab 36 26 23 16 15 13 11 10 3
E
Progression-free survival of NSCLC patients with baseline brain metastasis received nivolumab or nivolumab/ipilimumab
Progression Free Survival (%)
Median (days, 95% CI)
HR (95% CI)
Nivolumab
63.5 (56.0-98.0)
Reference
Nivlumab / Ipilimmab
160.0 (49.0-NA)
0.61 (0.26-1.40)
p = 0.24
Months
Number at risk
Nivolumab 36 11 3 2 1 1 1 0 0
Nivolumab /Ipilimumab 8 4 2 1 0 0 0 0 0
F
Overall survival of NSCLC patients with baseline brain metastasis received nivolumab or nivolumab/ipilimumab
Overall Survival (%)
Median (months, 95% CI)
HR (95% CI)
Nivolumab
15.9 (9.9-46.4)
Reference
Nivlumab / Ipilimmab
6.9 (5.0-NA)
2.53 (1.10-5.82)
p = 0.023
Months
Number at risk
lumab 36 30 27 23 17 16 14 12 11
lumab numab 8 6 5 3 3 2 2 2 0

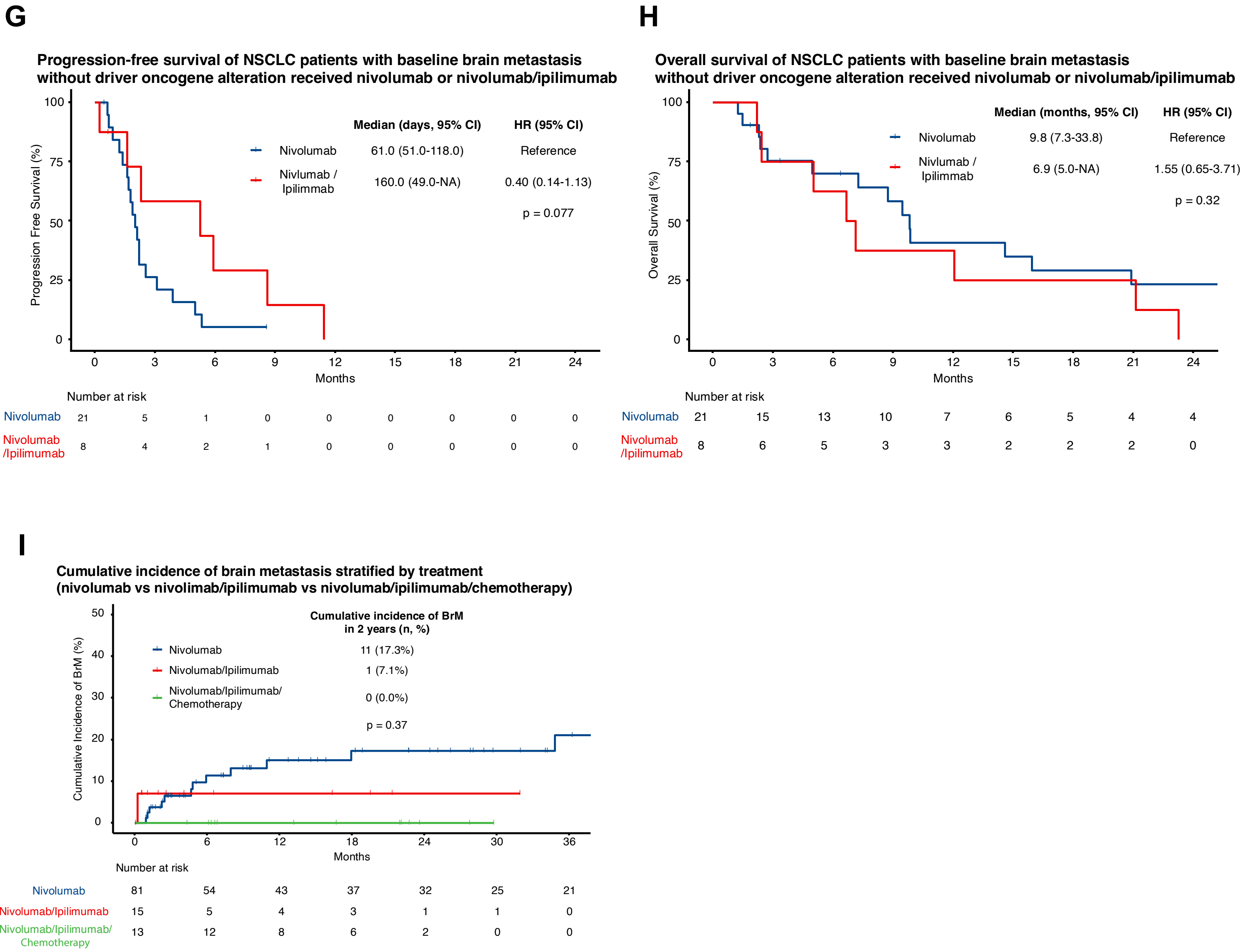
G
Progression-free survival of NSCLC patients with baseline brain metastasis without driver oncogene alteration received nivolumab or nivolumab/ipilimumab
Progression Free Survival (%)
Median (days, 95% CI)
HR (95% CI)
Nivolumab
61.0 (51.0-118.0)
Reference
Nivlumab / Ipilimmab
160.0 (49.0-NA)
0.40 (0.14-1.13)
p = 0.077
Months
Number at risk
Nivolumab 21 5 1 0 0 0 0 0 0
Nivolumab /Ipilimumab 8 4 2 1 0 0 0 0 0
H
Overall survival of NSCLC patients with baseline brain metastasis without driver oncogene alteration received nivolumab or nivolumab/ipilimumab
Overall Survival (%)
Median (months, 95% CI)
HR (95% CI)
Nivolumab
9.8 (7.3-33.8)
Reference
Nivlumab / Ipilimmab
6.9 (5.0-NA)
1.55 (0.65-3.71)
p = 0.32
Months
Number at risk
Nivolumab 21 15 13 10 7 6 5 4 4
Nivolumab /Ipilimumab 8 6 5 3 3 2 2 2 0
I
Cumulative incidence of brain metastasis stratified by treatment (nivolumab vs nivolimab/ipilimumab vs nivolumab/ipilimumab/chemotherapy)
Cumulative Incidence of BrM (%)
Cumulative incidence of BrM in 2 years (n, %)
Nivolumab 11 (17.3%)
Nivolumab/Ipilimumab 1 (7.1%)
Nivolumab/Ipilimumab/ Chemotherapy 0 (0.0%)
p = 0.37
Months
Number at risk
Nivolumab 81 54 43 37 32 25 21
Nivolumab/Ipilimumab 15 5 4 3 1 1 0
Nivolumab/Ipilimumab/ Chemotherapy 13 12 8 6 2 0 0

Supplementary Figure 1

A. Kaplan–Meier analysis of OS in NSCLC patients receiving nivolumab monotherapy. Survival was not significantly different between patients with or without baseline BrM. Statistical significance was assessed using a two-sided log-rank test.

B. Kaplan–Meier analysis of OS in NSCLC patients receiving nivolumab plus ipilimumab. Survival was not significantly different between patients with or without baseline BrM.

C. Kaplan–Meier analysis of progression-free survival (PFS) in all NSCLC patients regardless of baseline BrM status, comparing nivolumab monotherapy with nivolumab plus ipilimumab. PFS was comparable between the two regimens. Statistical significance was assessed using a two-sided log-rank test.

D. Kaplan–Meier analysis of OS in all NSCLC patients regardless of baseline BrM status, comparing nivolumab monotherapy with nivolumab plus ipilimumab. OS was comparable between the two regimens. Statistical significance was assessed using a two-sided log-rank test.

E. Kaplan–Meier analysis of PFS among patients with baseline BrM, comparing nivolumab monotherapy with nivolumab plus ipilimumab. PFS was comparable between the two regimens. Statistical significance was assessed using a two-sided log-rank test.

F. Kaplan–Meier analysis of OS among patients with baseline BrM, comparing nivolumab monotherapy with nivolumab plus ipilimumab. OS was significantly shorter in the nivolumab plus ipilimumab group. Statistical significance was assessed using a two-sided log-rank test.

G. Kaplan–Meier analysis of PFS among patients with baseline BrM after excluding those with driver oncogene alterations, comparing nivolumab monotherapy with nivolumab plus ipilimumab. PFS was comparable between the two regimens. Statistical significance was assessed using a two-sided log-rank test.

H. Kaplan–Meier analysis of OS among patients with baseline BrM after excluding those with driver oncogene alterations, comparing nivolumab monotherapy with nivolumab plus ipilimumab. The OS was comparable between the two regimens. Statistical significance was assessed using a two-sided log-rank test.

I. Cumulative incidence of new BrM in patients without baseline BrM, stratified by treatment regimen. Patients receiving nivolumab plus ipilimumab, with or without chemotherapy, showed a trend toward a lower incidence of new BrM compared with nivolumab monotherapy. Statistical significance was assessed using a two-sided log-rank test.

OS, overall survival; NSCLC, non-small-cell lung cancer; PFS, progression-free survival; BrM, brain metastasis.

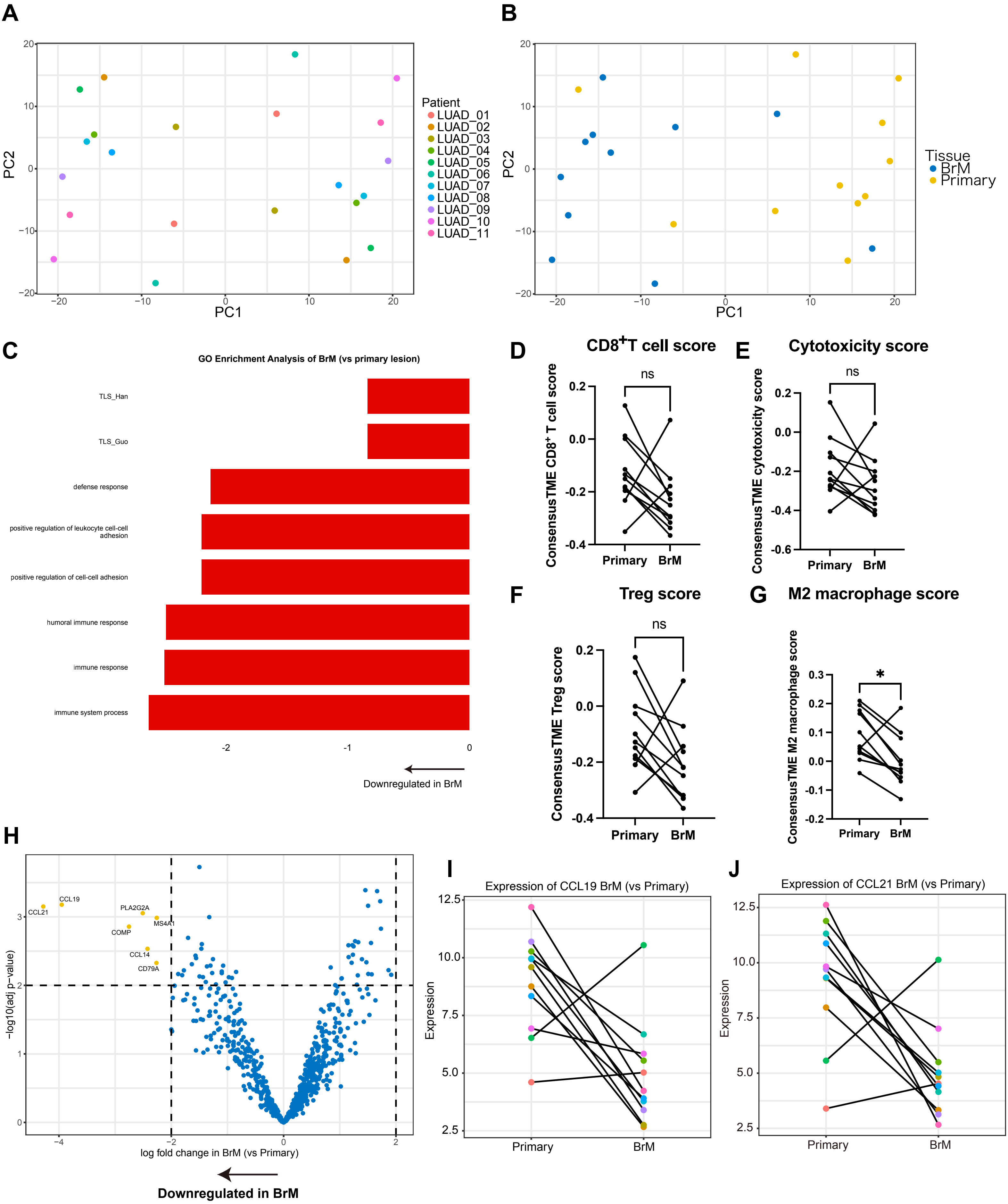
A
B
C
D
E
F
G
H
I
J
PC1
PC2
Patient
LUAD_01
LUAD_02
LUAD_03
LUAD_04
LUAD_05
LUAD_06
LUAD_07
LUAD_08
LUAD_09
LUAD_10
LUAD_11
Tissue
BrM
Primary
GO Enrichment Analysis of BrM (vs primary lesion)
TLS_Han
TLS_Guo
defense response
positive regulation of leukocyte cell-cell adhesion
positive regulation of cell-cell adhesion
humoral immune response
immune response
immune system process
Downregulated in BrM
CD8+T cell score
ConsensusTME CD8+ T cell score
ns
Primary
BrM
Cytotoxicity score
ConsensusTME cytotoxicity score
Treg score
ConsensusTME Treg score
M2 macrophage score
ConsensusTME M2 macrophage score
*
CCL19
CCL21
PLA2G2A
MS4A1
COMP
CCL14
CD79A
−log10(adj p−value)
log fold change in BrM (vs Primary)
Downregulated in BrM
Expression of CCL19 BrM (vs Primary)
Expression
Expression of CCL21 BrM (vs Primary)

Supplementary Figure 2

A, B. Using the Najjary et al. validation dataset, principal component analysis (PCA) reveals that gene expression profiles are clearly separated by tissue type (B; primary vs. BrM) rather than by individual patient origin (A), confirming the findings from the primary dataset.

C. Gene set enrichment analysis (GSEA) confirms the downregulation in BrM of gene ontology (GO) pathways related to immune response and tertiary lymphoid structures. The normalized enrichment score (NES) is shown.

D-G. Consistent with the primary dataset, gene set variation analysis (GSVA) scores show trends toward reduced scores of $CD8^+$ T cells (D), cytotoxicity (E), and Tregs (F) in BrM, although not statistically significant. The M2 macrophage score (G) showed reduced score in BrM compared with primary lesion. Statistical significance was assessed using a two-sided pairedWilcoxon matched-pairs signed-rank test. Exact P values are provided in the Source Data file.

H. Volcano plot of differentially expressed genes, highlighting that chemokines *CCL19* and *CCL21* are significantly downregulated in BrM.

I, J. Paired analysis confirms the significantly lower expression of chemokines *CCL19* (I) and *CCL21* (J) in BrM compared with primary tumors.

BrM, brain metastasis; PCA, principal component analysis; GSEA, gene set enrichment analysis; GO, Gene Ontology; NES, normalized enrichment score; GSVA, gene set variation analysis; Treg, regulatory T cell.

** $P < 0.01$.

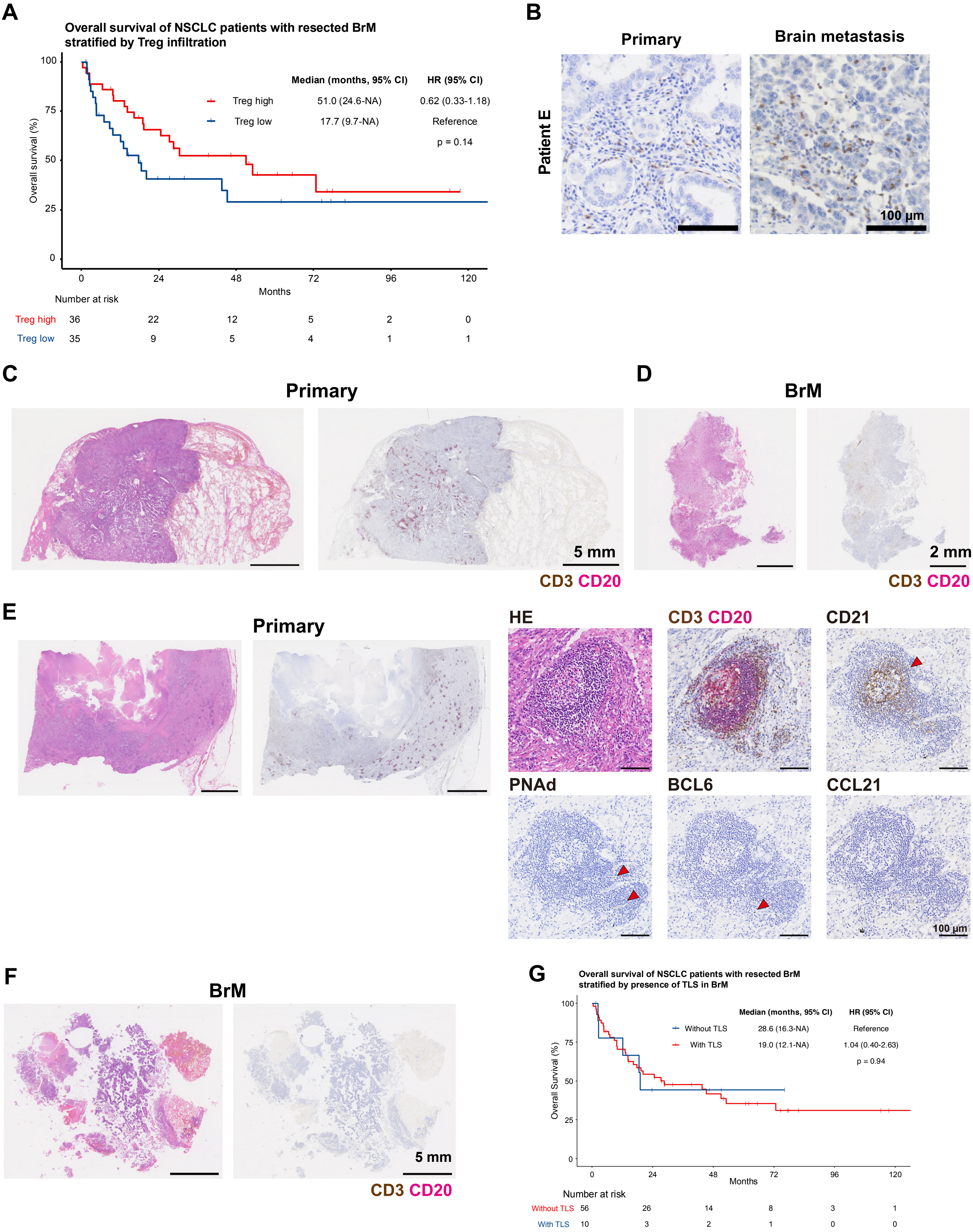
A
Overall survival of NSCLC patients with resected BrM
stratified by Treg infiltration
Median (months, 95% CI)
HR (95% CI)
Treg high
51.0 (24.6-NA)
0.62 (0.33-1.18)
Treg low
17.7 (9.7-NA)
Reference
p = 0.14
Overall survival (%)
100
75
50
25
0
0
24
48
72
96
120
Months
Number at risk
Treg high 36 22 12 5 2 0
Treg low 35 9 5 4 1 1
B
Primary
Brain metastasis
Patient E
100 µm
C
Primary
5 mm
CD3 CD20
D
BrM
2 mm
CD3 CD20
E
Primary
HE
CD3 CD20
CD21
PNAd
BCL6
CCL21
100 µm
F
BrM
5 mm
CD3 CD20
G
Overall survival of NSCLC patients with resected BrM
stratified by presence of TLS in BrM
Median (months, 95% CI)
HR (95% CI)
Without TLS
28.6 (16.3-NA)
Reference
With TLS
19.0 (12.1-NA)
1.04 (0.40-2.63)
p = 0.94
Overall Survival (%)
100
75
50
25
0
0
24
48
72
96
120
Months
Number at risk
Without TLS 56 26 14 8 3 1
With TLS 10 3 2 1 0 0

Supplementary Figure 3

A. Kaplan–Meier analysis of post-resection OS stratified by Treg infiltration in BrM. Treg density was not significantly associated with survival. Statistical significance was assessed using a two-sided log-rank test.

B. Representative image of a BrM with markedly high CTL infiltration, from a patient who experienced long-term survival post-resection.

C, D. Low-magnification overview images of a primary tumor (C) and its corresponding BrM (D), showing H&E and immunohistochemical staining for TLS markers (CD3, brown; CD20, pink).

E, F. Higher-magnification images from another representative patient, showing H&E and CD3/CD20 staining of a primary tumor (E) and BrM (F) to detect TLS.

G. Kaplan–Meier analysis of post-resection OS stratified by the presence of TLS in BrM. The presence of TLS was not significantly associated with survival. Statistical significance was assessed using a two-sided log-rank test.

BrM, brain metastasis; CTL, cytotoxic T lymphocyte; OS, overall survival; Treg, regulatory T cell; TLS, tertiary lymphoid structure.

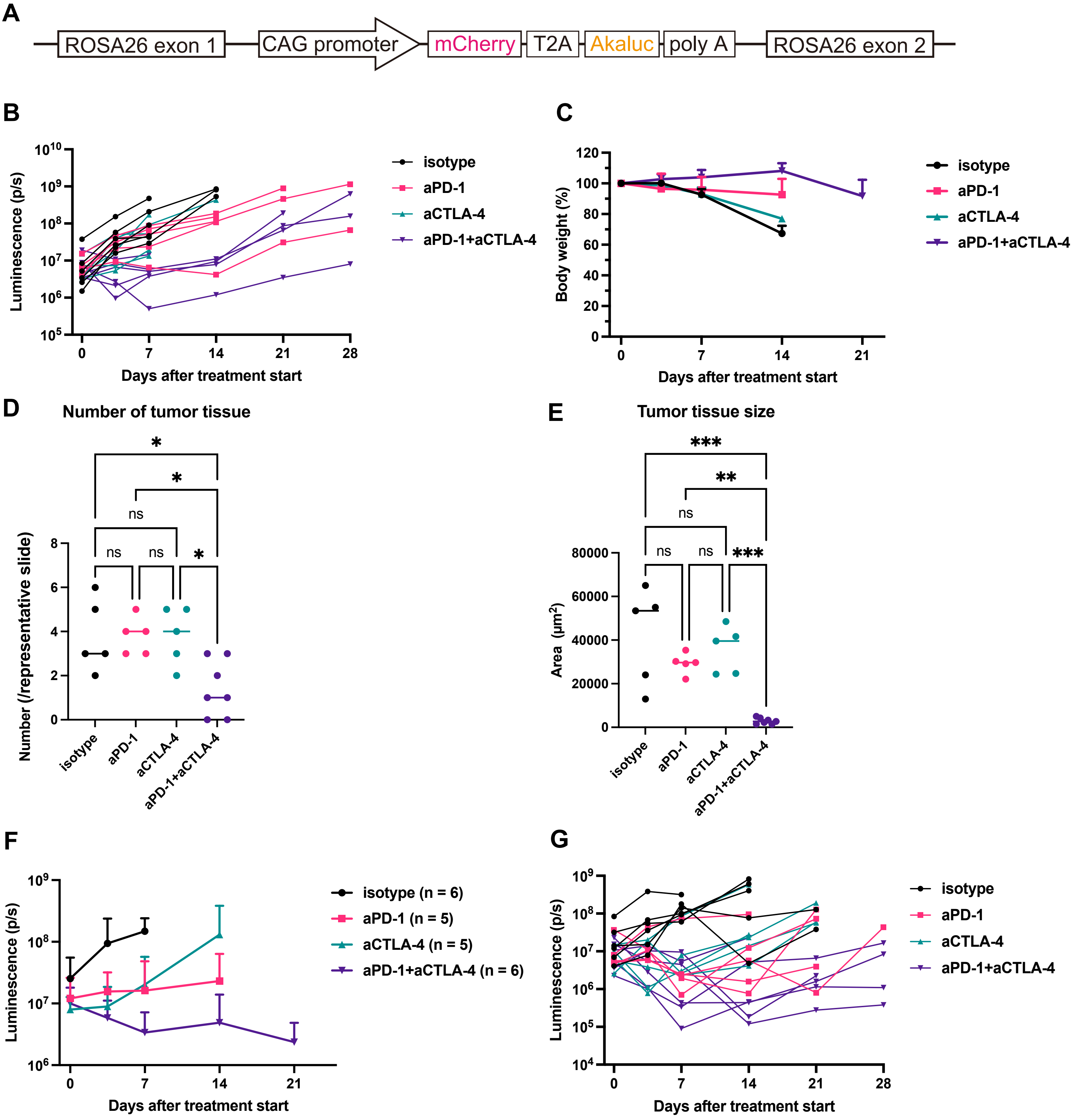
A
ROSA26 exon 1
CAG promoter
mCherry
T2A
Akaluc
poly A
ROSA26 exon 2
B
Luminescence (p/s)
Days after treatment start
isotype
aPD-1
aCTLA-4
aPD-1+aCTLA-4
C
Body weight (%)
Days after treatment start
isotype
aPD-1
aCTLA-4
aPD-1+aCTLA-4
D
Number of tumor tissue
Number (/representative slide)
ns
*
isotype
aPD-1
aCTLA-4
aPD-1+aCTLA-4
E
Tumor tissue size
Area (μm²)
***
**
ns
isotype
aPD-1
aCTLA-4
aPD-1+aCTLA-4
F
Luminescence (p/s)
Days after treatment start
isotype (n = 6)
aPD-1 (n = 5)
aCTLA-4 (n = 5)
aPD-1+aCTLA-4 (n = 6)
G
Luminescence (p/s)
Days after treatment start
isotype
aPD-1
aCTLA-4
aPD-1+aCTLA-4

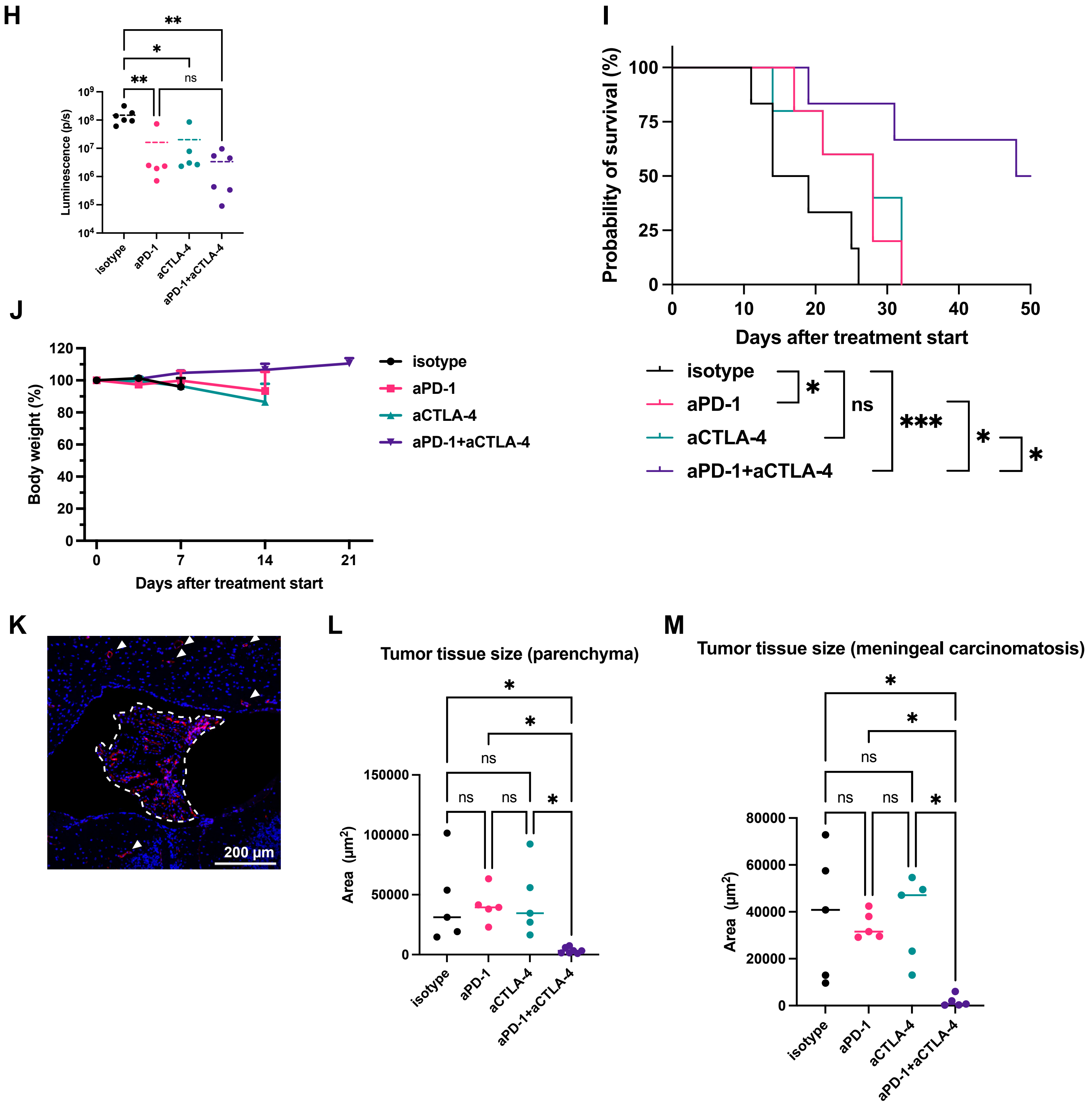
H
Luminescence (p/s)
10^4 10^5 10^6 10^7 10^8 10^9
isotype
aPD-1
aCTLA-4
aPD-1+aCTLA-4
**
*
**
ns
I
Probability of survival (%)
0 25 50 75 100
0 10 20 30 40 50
Days after treatment start
isotype
aPD-1
aCTLA-4
aPD-1+aCTLA-4
*
ns
***
*
*
J
Body weight (%)
0 20 40 60 80 100 120
0 7 14 21
Days after treatment start
isotype
aPD-1
aCTLA-4
aPD-1+aCTLA-4
K
200 μm
L
Tumor tissue size (parenchyma)
Area (μm²)
0 50000 100000 150000
isotype
aPD-1
aCTLA-4
aPD-1+aCTLA-4
*
*
ns
ns
ns
*
M
Tumor tissue size (meningeal carcinomatosis)
Area (μm²)
0 20000 40000 60000 80000
isotype
aPD-1
aCTLA-4
aPD-1+aCTLA-4
*
*
ns
ns
ns
*

Supplementary Figure 4

A. Schematic representation of the gene-editing process used to establish luminescent and fluorescently tagged cancer cell lines.

B. Longitudinal analysis of tumor burden in individual mice in the CMT167-derived BrM model (mean ± SD; isotype, n = 6; aPD-1, n = 5; aCTLA-4, n = 5; aPD-1+aCTLA-4, n = 6). Data are combined from 3 independent experiments.

C. Longitudinal body weight change in the CMT167-derived BrM model (mean ± SD; isotype, n = 6; aPD-1, n = 5; aCTLA-4, n = 5; aPD-1+aCTLA-4, n = 6). Data are combined from 3 independent experiments.

D. Quantification of tumor foci from IF images of CMT167-derived BrMs showing reduced tumor burden with combination therapy. Each dot represents data from an individual mouse (mean ± SD; isotype, n = 6; aPD-1, n = 5; aCTLA-4, n = 5; aPD-1+aCTLA-4, n = 9). Data are combined from 3 independent experiments. Statistical significance was assessed using Kruskal–Wallis followed by Dunn's test.

E. Quantification of tumor area from IF images of CMT167-derived BrMs showing reduced tumor burden with combination therapy. Each dot represents mean area of tumor tissues from an individual mouse (mean ± SD; isotype, n = 5; aPD-1, n = 5; aCTLA-4, n = 5; aPD-1+aCTLA-4, n = 7). Data are combined from 3 independent experiments. Statistical significance was assessed using Kruskal–Wallis followed by Dunn's test.

F–I. Data from the LLC-derived BrM model confirmed the efficacy of combination therapy. Longitudinal analysis showed tumor growth inhibition (F, G). Comparison at day 7 and longitudinal analysis of tumor burden in individual mice (H) showed a significantly reduced tumor burden. Statistical significance was assessed using Kruskal–Wallis followed by Dunn's test. Kaplan–Meier analysis revealed prolonged survival (I). Statistical significance was assessed using a two-sided log-rank test. Longitudinal analysis of body weight changes in the LLC-derived BrM model showed no significant body weight loss by the therapy (mean ± SD; isotype, n = 6; aPD-1, n = 5; aCTLA-4, n = 5; aPD-1 + aCTLA-4, n = 6). Each dot and line represents one mouse. Data are combined from 3 independent experiments.

K. Representative immunofluorescence images of the BrM model with ICA injection illustrating the presence of parenchymal metastases (indicated by arrowheads) and meningeal carcinomatosis (outlined by a dashed line). Images were obtained from the CMT167 ICA injection model at day 3 post-injection to facilitate discrimination of early parenchymal seeding from meningeal/CSF-space involvement, as parenchymal lesions are not yet fully established at this time point.

L. Quantification of tumor size within the brain parenchyma showing that combination therapy significantly reduced tumor burden compared with both the isotype control and anti-

PD-1 monotherapy. Each dot represents mean area of tumor tissues from an individual mouse (mean ± SD; isotype, n = 5; aPD-1, n = 5; aCTLA-4, n = 5; aPD-1+aCTLA-4, n = 7). Data are combined from 4 independent experiments. Statistical significance was assessed using Kruskal–Wallis followed by Dunn’s test.

M. Quantification of tumor size in meningeal carcinomatosis showing that combination therapy significantly reduced tumor burden compared with the isotype control. Each dot represents mean area of tumor tissues from an individual mouse (mean ± SD; isotype, n = 5; aPD-1, n = 5; aCTLA-4, n = 5; aPD-1+aCTLA-4, n = 5). Data are combined from 3 independent experiments. Statistical significance was assessed using Kruskal–Wallis followed by Dunn’s test.

BrM, brain metastasis; CTL, cytotoxic T lymphocyte; ICA, internal carotid artery; SD, standard deviation.

ns, not significant; * $P < 0.05$, ** $P < 0.01$, *** $P < 0.001$.

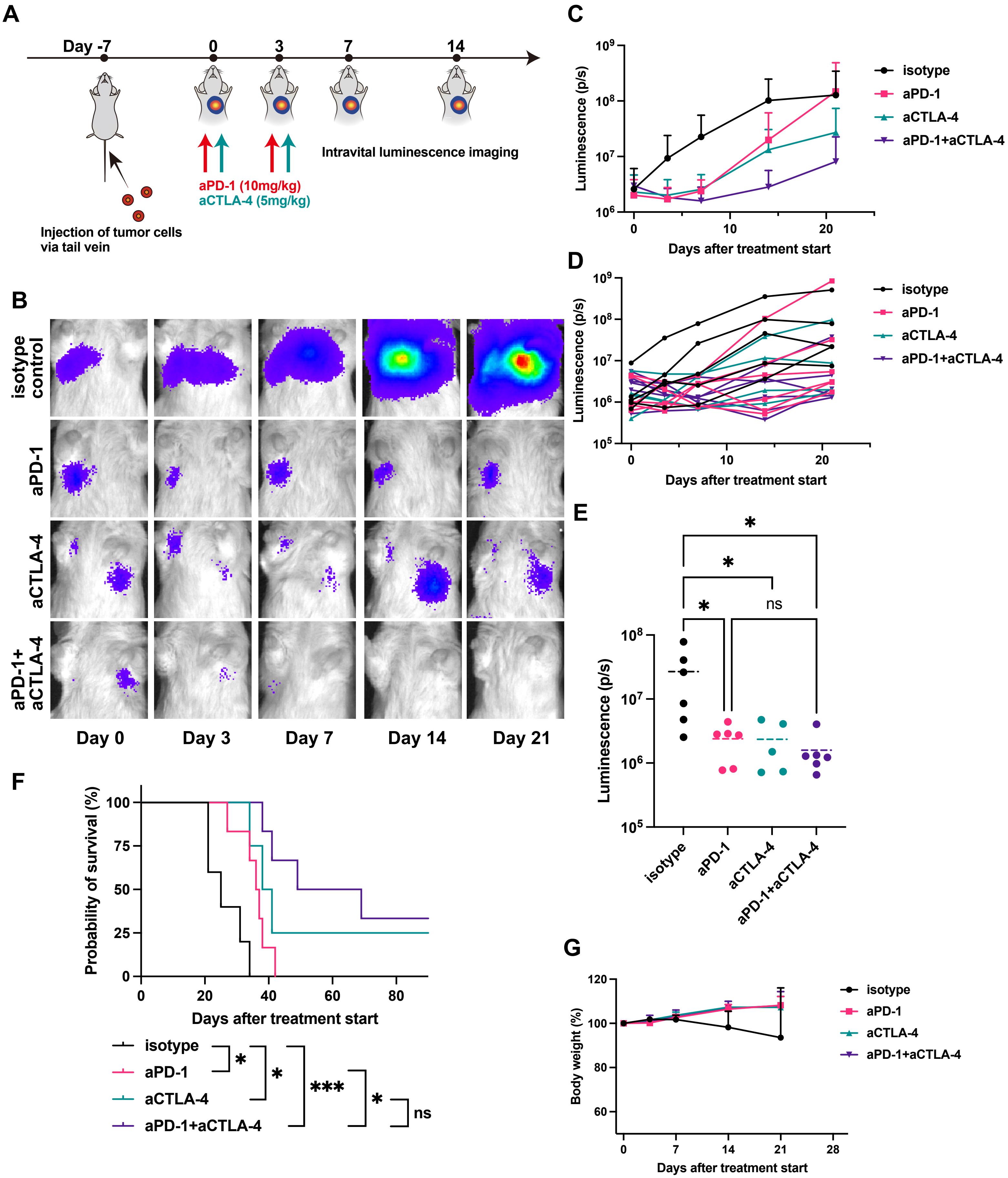
A
Day -7
0
3
7
14
Injection of tumor cells
via tail vein
aPD-1 (10mg/kg)
aCTLA-4 (5mg/kg)
Intravital luminescence imaging
B
isotype control
aPD-1
aCTLA-4
aPD-1+ aCTLA-4
Day 0
Day 3
Day 7
Day 14
Day 21
C
Luminescence (p/s)
Days after treatment start
isotype
aPD-1
aCTLA-4
aPD-1+aCTLA-4
D
Luminescence (p/s)
Days after treatment start
isotype
aPD-1
aCTLA-4
aPD-1+aCTLA-4
E
Luminescence (p/s)
*
*
*
ns
isotype
aPD-1
aCTLA-4
aPD-1+aCTLA-4
F
Probability of survival (%)
Days after treatment start
isotype
aPD-1
aCTLA-4
aPD-1+aCTLA-4
*
*
***
*
ns
G
Body weight (%)
Days after treatment start
isotype
aPD-1
aCTLA-4
aPD-1+aCTLA-4

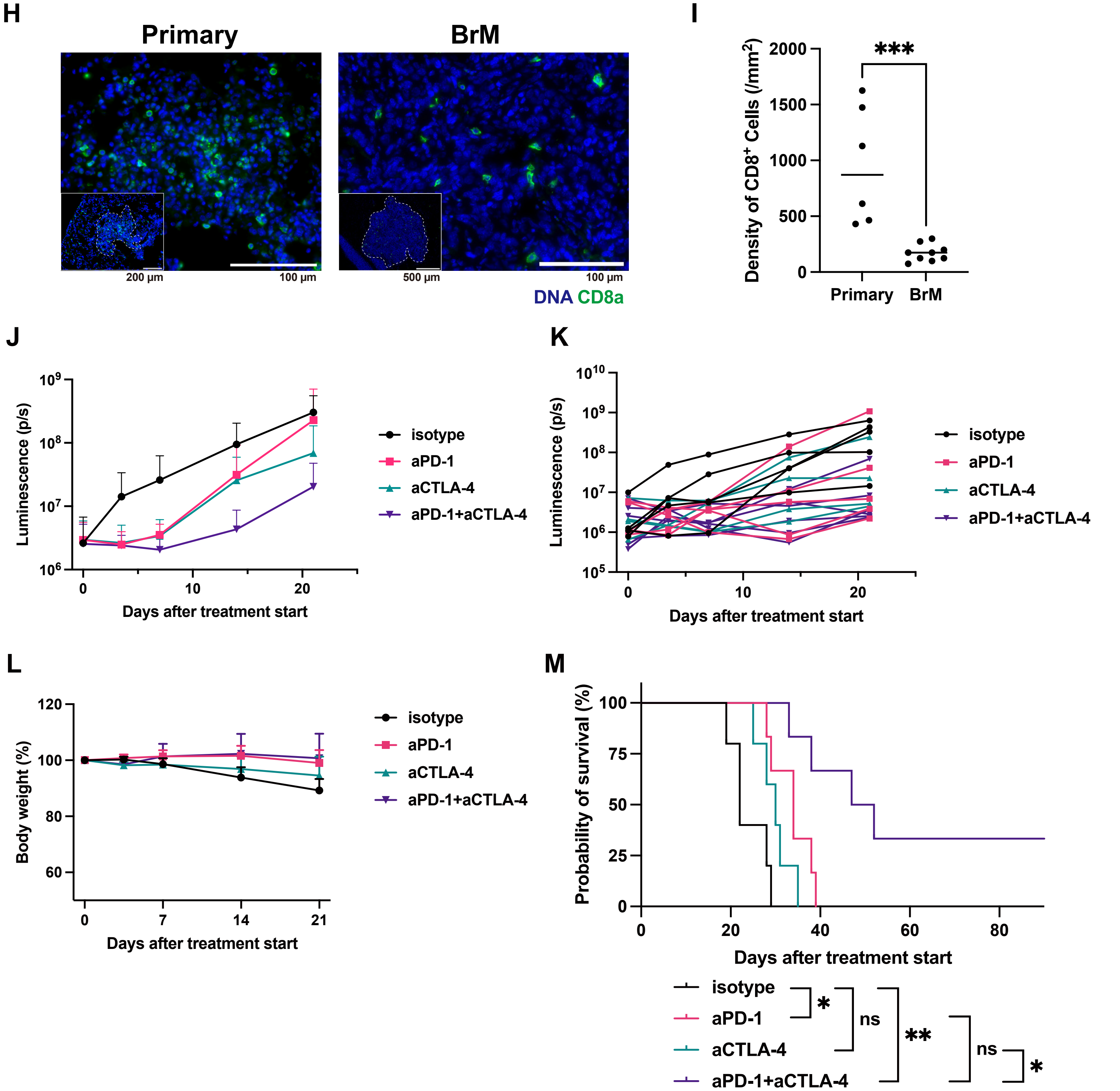
H
Primary
BrM
200 µm
100 µm
500 µm
100 µm
DNA CD8a
I
Density of CD8+ Cells (/mm2)
2000
1500
1000
500
0
***
Primary
BrM
J
Luminescence (p/s)
10^9
10^8
10^7
10^6
0
10
20
Days after treatment start
isotype
aPD-1
aCTLA-4
aPD-1+aCTLA-4
K
Luminescence (p/s)
10^10
10^9
10^8
10^7
10^6
10^5
0
10
20
Days after treatment start
isotype
aPD-1
aCTLA-4
aPD-1+aCTLA-4
L
Body weight (%)
120
100
80
60
0
7
14
21
Days after treatment start
isotype
aPD-1
aCTLA-4
aPD-1+aCTLA-4
M
Probability of survival (%)
100
75
50
25
0
0
20
40
60
80
Days after treatment start
isotype
aPD-1
aCTLA-4
aPD-1+aCTLA-4
*
ns
**
ns
*

Supplementary Figure 5

A. Schematic representation of the primary lung tumor model established via tail vein injection and treatment time course.

B. Representative longitudinal in vivo luminescence images of mice with primary lung tumor treated with isotype control or indicated immunotherapies.

C, D. Longitudinal analysis of tumor burden in the primary lung tumor model, showing group averages (C, mean ± SD) and individual mouse trajectories (D) (isotype, n = 6; aPD-1, n = 6; aCTLA-4, n = 5; aPD-1+aCTLA-4, n = 6). Both monotherapies show antitumor activity, while combination therapy shows the most potent effect.

E. Comparison of tumor burden at day 7 (isotype, n = 6; aPD-1, n = 6; aCTLA-4, n = 5; aPD-1+aCTLA-4, n = 6). Both anti-PD-1 and anti-PD-1 plus anti-CTLA-4 combination therapy significantly reduced tumor burden compared with the isotype control.

F. Kaplan–Meier survival curves demonstrating that combination therapy significantly prolonged survival in the primary lung tumor model (isotype, n = 6; aPD-1, n = 6; aCTLA-4, n = 5; aPD-1+aCTLA-4, n = 6).

G. Longitudinal analysis of body weight, showing no significant treatment-related toxicity across all groups (isotype, n = 6; aPD-1, n = 6; aCTLA-4, n = 5; aPD-1+aCTLA-4, n = 6).

H, I. Representative immunofluorescence images (H) and quantification (I) comparing baseline immune infiltration between the primary lung tumor model and the BrM model. The density of $CD8^+$ T cells is significantly lower in the BrM model. Each dot represents the average infiltration density from individual mice (primary, n = 6; BrM, n = 9).

J. Longitudinal analysis of tumor burden in the LLC-derived primary lung tumor model, showing group averages (mean ± SD) (isotype, n = 5; aPD-1, n = 6; aCTLA-4, n = 5; aPD-1+aCTLA-4, n = 6). Both monotherapies show moderate antitumor activity, while combination therapy shows the most potent effect.

K. Longitudinal tumor growth curves for individual mice (isotype, n = 5; aPD-1, n = 6; aCTLA-4, n = 5; aPD-1+aCTLA-4, n = 6).

L. Longitudinal analysis of body weight, showing no significant treatment-related toxicity across all groups (isotype, n = 5; aPD-1, n = 6; aCTLA-4, n = 5; aPD-1+aCTLA-4, n = 6).

M. Kaplan–Meier survival curves. Combination therapy significantly prolonged survival compared with isotype control and both monotherapies (isotype, n = 5; aPD-1, n = 6; aCTLA-4, n = 5; aPD-1+aCTLA-4, n = 6).

SD, standard deviation.

ns, not significant; * $P < 0.05$, ** $P < 0.01$, *** $P < 0.001$.

A

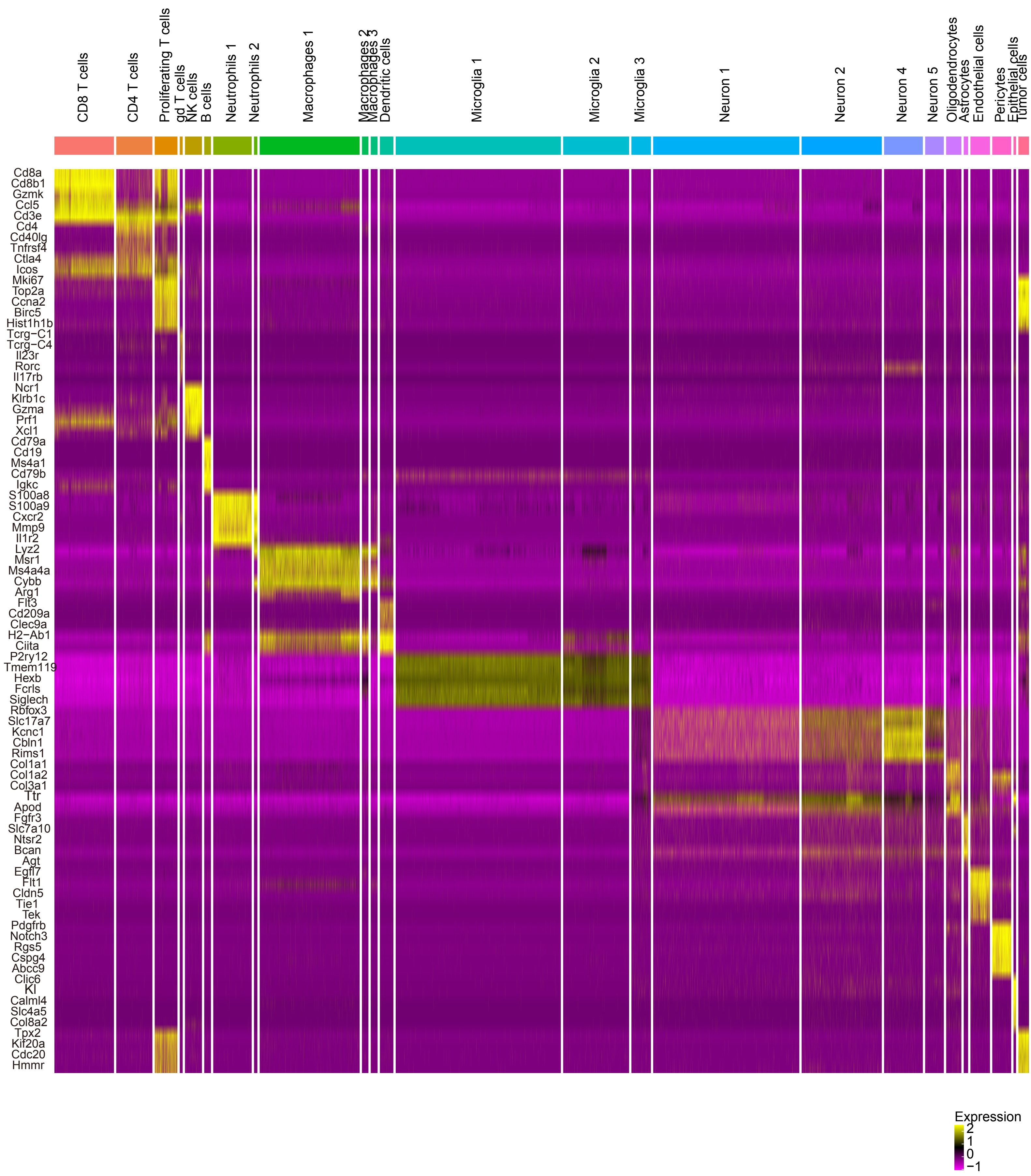

CD8 T cells
CD4 T cells
Proliferating T cells
gd T cells
NK cells
B cells
Neutrophils 1
Neutrophils 2
Macrophages 1
Macrophages 2
Macrophages 3
Dendritic cells
Microglia 1
Microglia 2
Microglia 3
Neuron 1
Neuron 2
Neuron 4
Neuron 5
Oligodendrocytes
Astrocytes
Endothelial cells
Pericytes
Epithelial cells
Tumor cells
Cd8a
Cd8b1
Gzmk
Ccl5
Cd3e
Cd4
Cd40lg
Tnfrsf4
Ctla4
Icos
Mki67
Top2a
Ccna2
Birc5
Hist1h1b
Tcrg−C1
Tcrg−C4
Il23r
Rorc
Il17rb
Ncr1
Klrb1c
Gzma
Prf1
Xcl1
Cd79a
Cd19
Ms4a1
Cd79b
Igkc
S100a8
S100a9
Cxcr2
Mmp9
Il1r2
Lyz2
Msr1
Ms4a4a
Cybb
Arg1
Flt3
Cd209a
Clec9a
H2−Ab1
Ciita
P2ry12
Tmem119
Hexb
Fcrls
Siglech
Rbfox3
Slc17a7
Kcnc1
Cbln1
Rims1
Col1a1
Col1a2
Col3a1
Ttr
Apod
Fgfr3
Slc7a10
Ntsr2
Bcan
Agt
Egfl7
Flt1
Cldn5
Tie1
Tek
Pdgfrb
Notch3
Rgs5
Cspg4
Abcc9
Clic6
Kl
Calml4
Slc4a5
Col8a2
Tpx2
Kif20a
Cdc20
Hmmr
Expression
2
1
0
−1

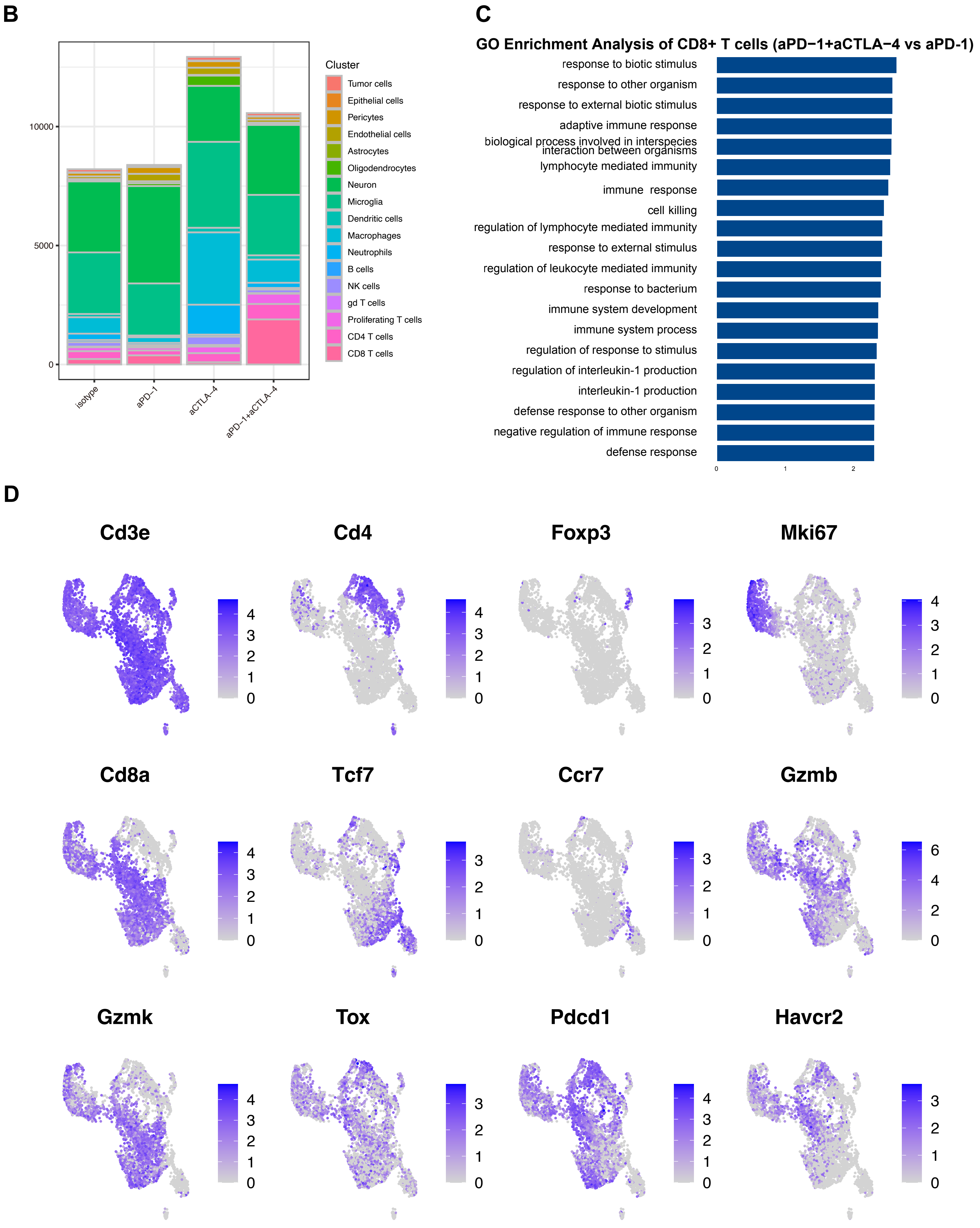
B
Cluster
Tumor cells
Epithelial cells
Pericytes
Endothelial cells
Astrocytes
Oligodendrocytes
Neuron
Microglia
Dendritic cells
Macrophages
Neutrophils
B cells
NK cells
gd T cells
Proliferating T cells
CD4 T cells
CD8 T cells
10000
5000
0
isotype
aPD−1
aCTLA−4
aPD−1+aCTLA−4
C
GO Enrichment Analysis of CD8+ T cells (aPD−1+aCTLA−4 vs aPD−1)
response to biotic stimulus
response to other organism
response to external biotic stimulus
adaptive immune response
biological process involved in interspecies interaction between organisms
lymphocyte mediated immunity
immune response
cell killing
regulation of lymphocyte mediated immunity
response to external stimulus
regulation of leukocyte mediated immunity
response to bacterium
immune system development
immune system process
regulation of response to stimulus
regulation of interleukin-1 production
interleukin-1 production
defense response to other organism
negative regulation of immune response
defense response
0
1
2
D
Cd3e
Cd4
Foxp3
Mki67
Cd8a
Tcf7
Ccr7
Gzmb
Gzmk
Tox
Pdcd1
Havcr2

E

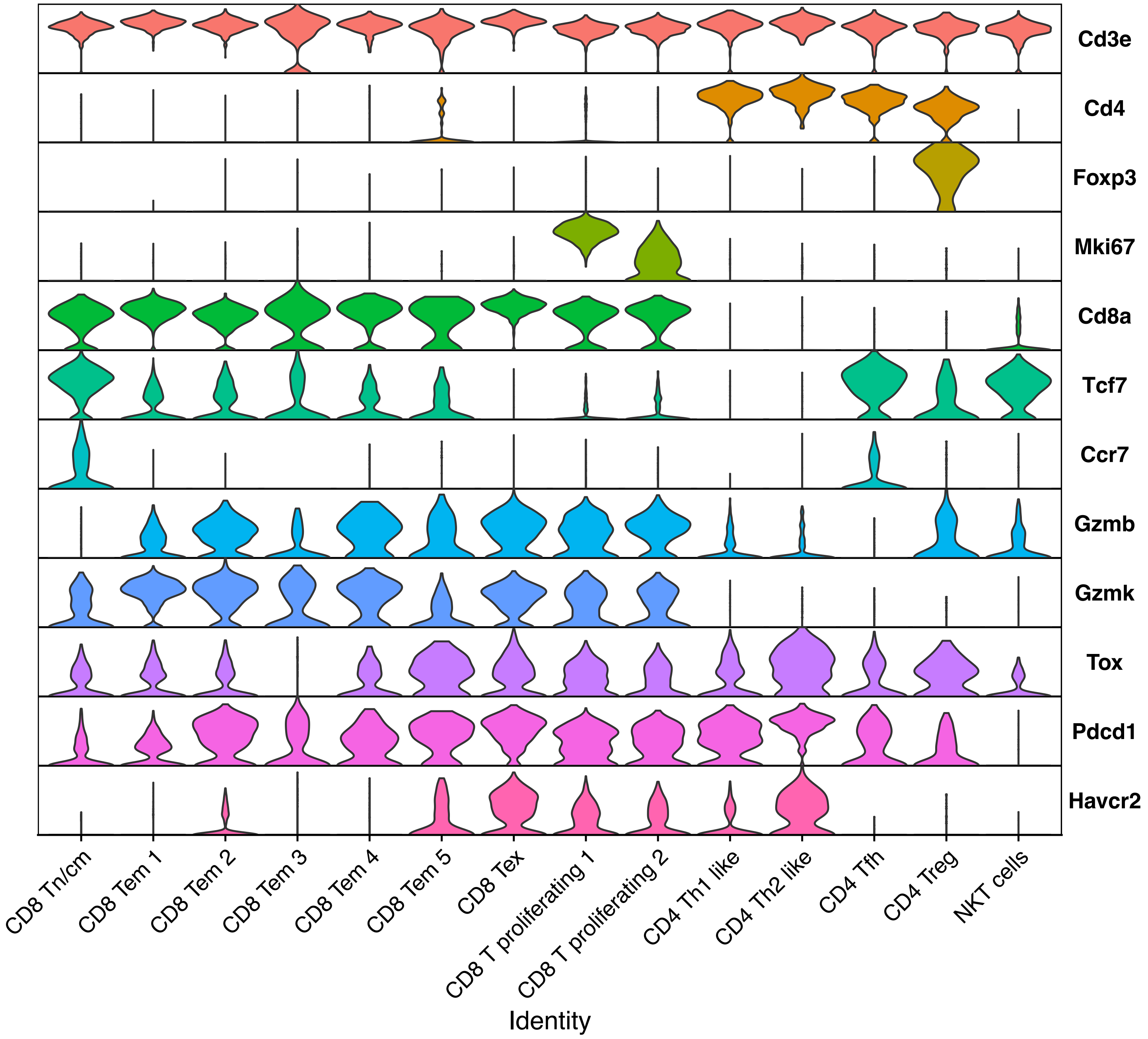
Cd3e
Cd4
Foxp3
Mki67
Cd8a
Tcf7
Ccr7
Gzmb
Gzmk
Tox
Pdcd1
Havcr2
CD8 Tn/cm
CD8 Tem 1
CD8 Tem 2
CD8 Tem 3
CD8 Tem 4
CD8 Tem 5
CD8 Tex
CD8 T proliferating 1
CD8 T proliferating 2
CD4 Th1 like
CD4 Th2 like
CD4 Tfh
CD4 Treg
NKT cells
Identity

F

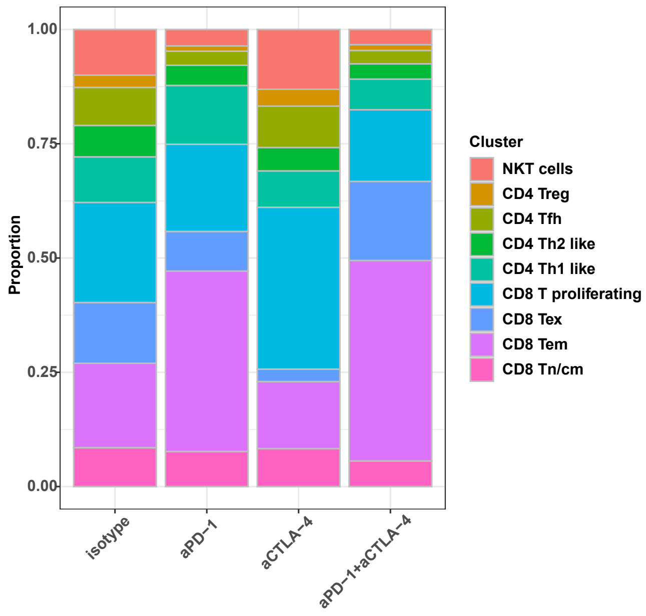
Proportion
1.00
0.75
0.50
0.25
0.00
isotype
aPD−1
aCTLA−4
aPD−1+aCTLA−4
Cluster
NKT cells
CD4 Treg
CD4 Tfh
CD4 Th2 like
CD4 Th1 like
CD8 T proliferating
CD8 Tex
CD8 Tem
CD8 Tn/cm

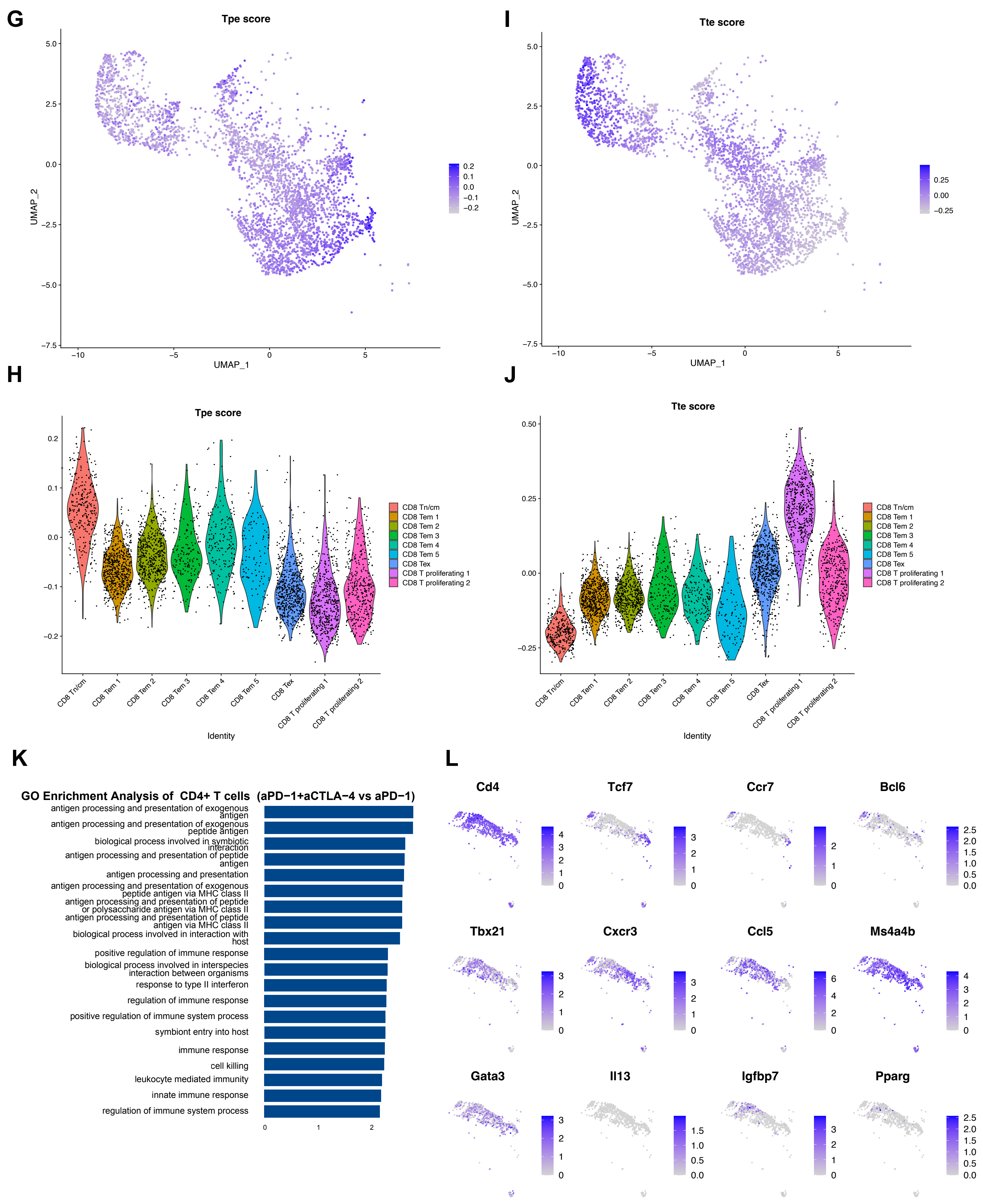
G
Tpe score
UMAP_2
UMAP_1
I
Tte score
H
Tpe score
Identity
J
Tte score
CD8 Tn/cm
CD8 Tem 1
CD8 Tem 2
CD8 Tem 3
CD8 Tem 4
CD8 Tem 5
CD8 Tex
CD8 T proliferating 1
CD8 T proliferating 2
K
GO Enrichment Analysis of CD4+ T cells (aPD−1+aCTLA−4 vs aPD−1)
antigen processing and presentation of exogenous antigen
antigen processing and presentation of exogenous peptide antigen
biological process involved in symbiotic interaction
antigen processing and presentation of peptide antigen
antigen processing and presentation
antigen processing and presentation of exogenous peptide antigen via MHC class II
antigen processing and presentation of peptide or polysaccharide antigen via MHC class II
antigen processing and presentation of peptide antigen via MHC class II
biological process involved in interaction with host
positive regulation of immune response
biological process involved in interspecies interaction between organisms
response to type II interferon
regulation of immune response
positive regulation of immune system process
symbiont entry into host
immune response
cell killing
leukocyte mediated immunity
innate immune response
regulation of immune system process
L
Cd4
Tcf7
Ccr7
Bcl6
Tbx21
Cxcr3
Ccl5
Ms4a4b
Gata3
Il13
Igfbp7
Pparg

M

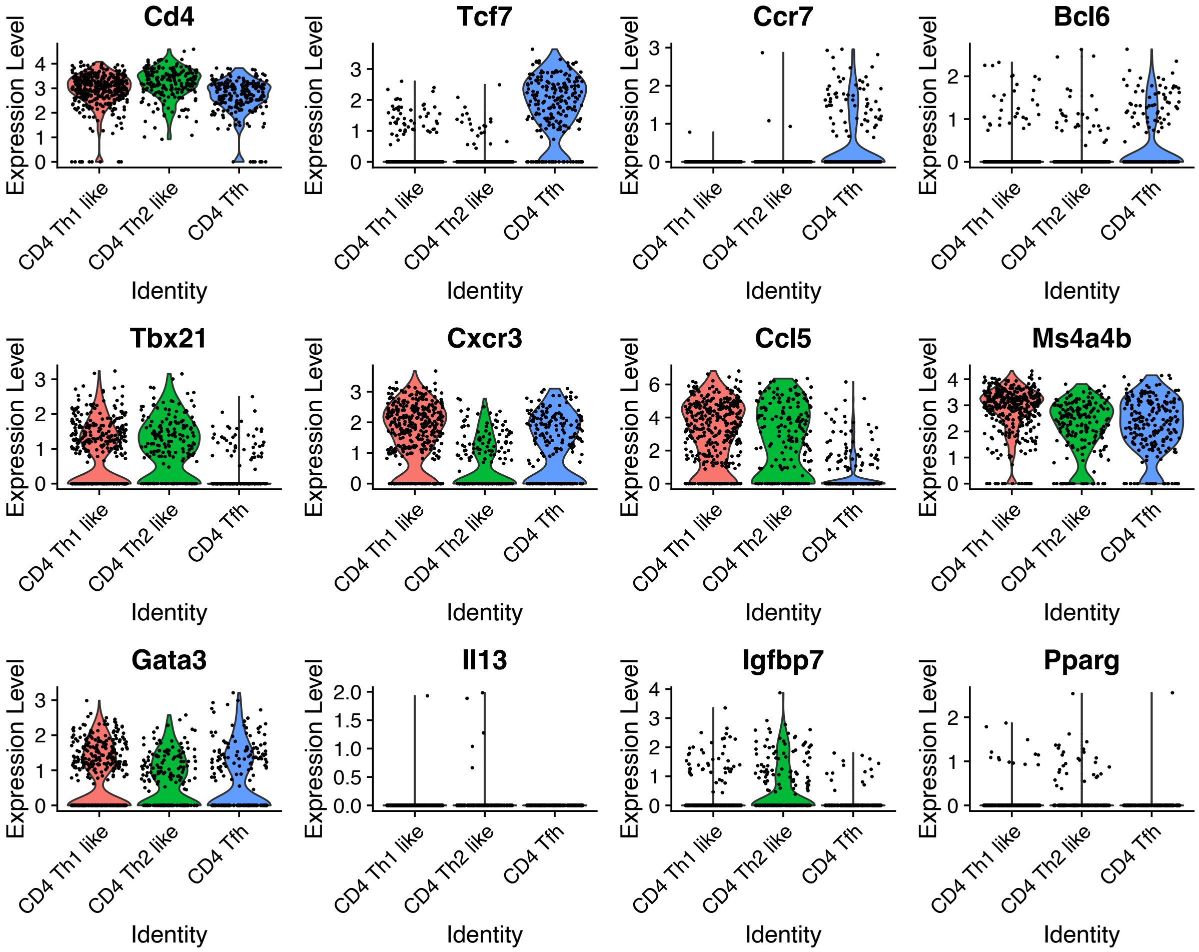
Cd4
Tcf7
Ccr7
Bcl6
Tbx21
Cxcr3
Ccl5
Ms4a4b
Gata3
Il13
Igfbp7
Pparg
Expression Level
CD4 Th1 like
CD4 Th2 like
CD4 Tfh
Identity

N

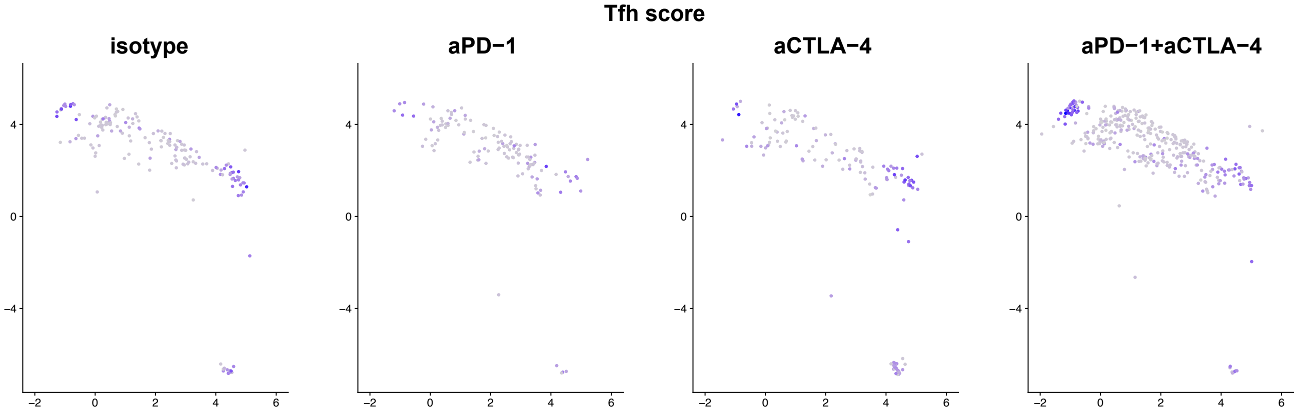
Tfh score
isotype
aPD−1
aCTLA−4
aPD−1+aCTLA−4

O

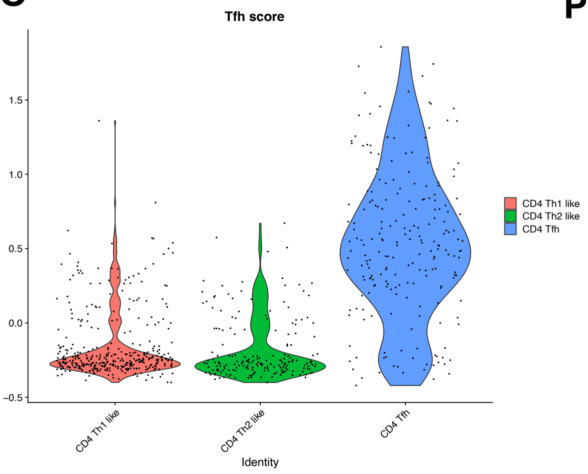
Tfh score
CD4 Th1 like
CD4 Th2 like
CD4 Tfh
Identity

P

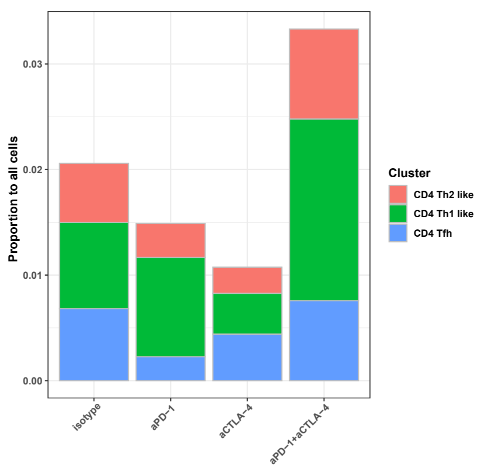
Proportion to all cells
Cluster
CD4 Th2 like
CD4 Th1 like
CD4 Tfh
isotype
aPD−1
aCTLA−4
aPD−1+aCTLA−4

Q

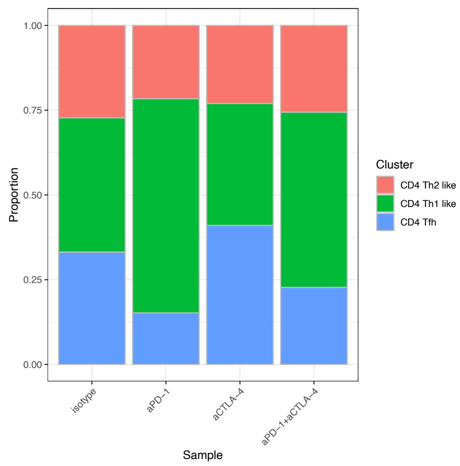
Proportion
Cluster
CD4 Th2 like
CD4 Th1 like
CD4 Tfh
isotype
aPD−1
aCTLA−4
aPD−1+aCTLA−4
Sample

R

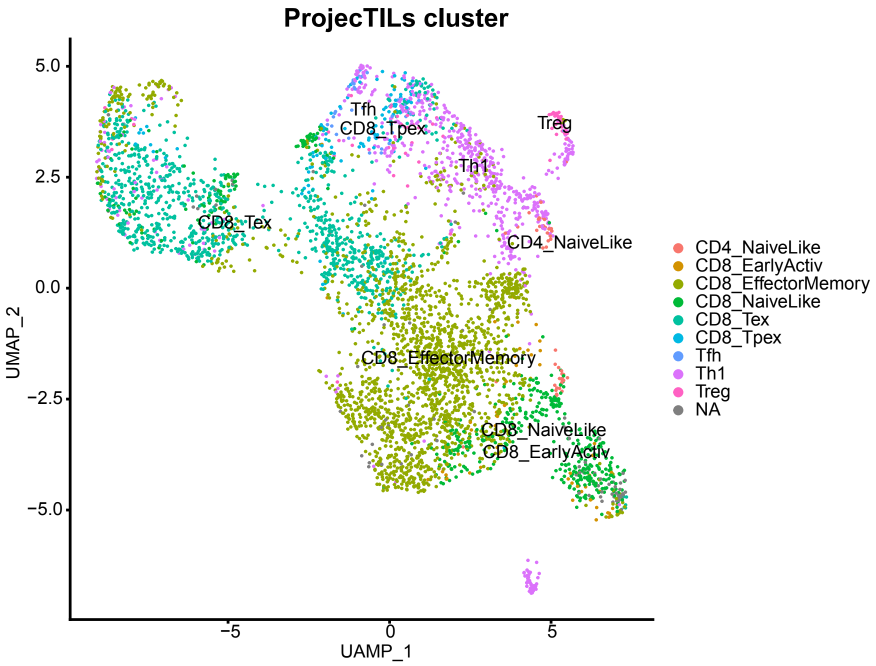
ProjecTILs cluster
UMAP_2
UAMP_1
5.0
2.5
0.0
−2.5
−5.0
−5
0
5
Tfh
CD8_Tpex
Treg
Th1
CD8_Tex
CD4_NaiveLike
CD8_EffectorMemory
CD8_NaiveLike
CD8_EarlyActiv
CD4_NaiveLike
CD8_EarlyActiv
CD8_EffectorMemory
CD8_NaiveLike
CD8_Tex
CD8_Tpex
Tfh
Th1
Treg
NA

S

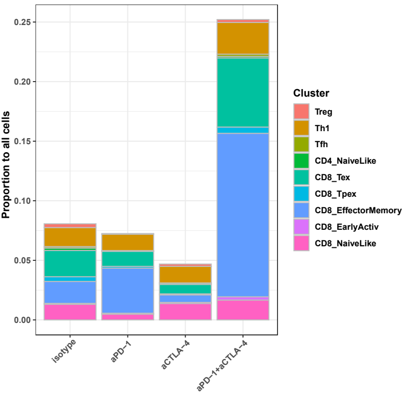
Proportion to all cells
Cluster
Treg
Th1
Tfh
CD4_NaiveLike
CD8_Tex
CD8_Tpex
CD8_EffectorMemory
CD8_EarlyActiv
CD8_NaiveLike
isotype
aPD−1
aCTLA−4
aPD−1+aCTLA−4

T

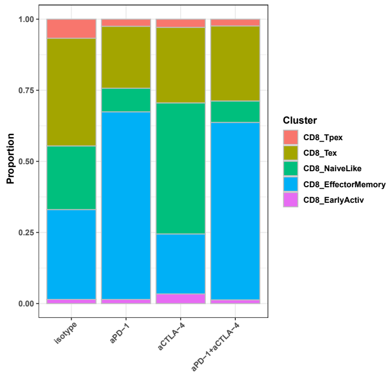
Proportion
Cluster
CD8_Tpex
CD8_Tex
CD8_NaiveLike
CD8_EffectorMemory
CD8_EarlyActiv
isotype
aPD−1
aCTLA−4
aPD−1+aCTLA−4

U

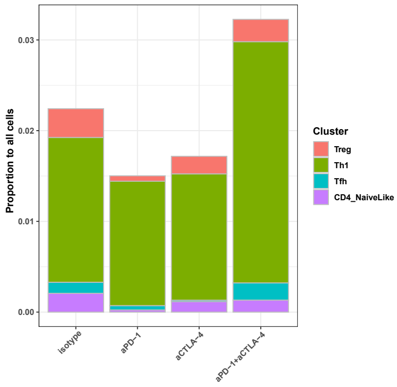
Proportion to all cells
Cluster
Treg
Th1
Tfh
CD4_NaiveLike
isotype
aPD−1
aCTLA−4
aPD−1+aCTLA−4

V

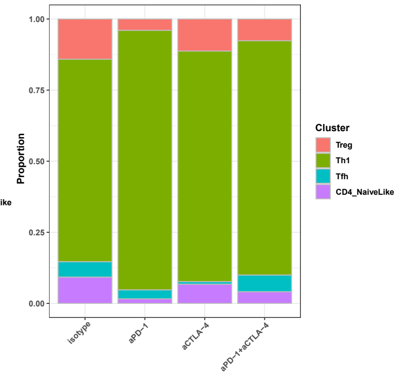
Proportion
Cluster
Treg
Th1
Tfh
CD4_NaiveLike
isotype
aPD−1
aCTLA−4
aPD−1+aCTLA−4

Supplementary Figure 6

A. Heatmap of marker gene expression used to annotate the major cell clusters identified in brain metastasis (BrM).

B. Stacked bar plot showing the number of cells per cluster across treatment groups, demonstrating an increase in T cell populations, particularly CD8$^+$ T cells, in the combination therapy group.

C. Gene Ontology (GO) enrichment analysis of differentially expressed genes (DEGs) in CD8$^+$ T cells comparing combination therapy to anti-PD-1 monotherapy, showing upregulation of pathways related to adaptive immunity.

D. UMAP visualizations showing the expression of canonical marker genes used to identify and validate T cell subsets.

E. Violin plots confirming the expression patterns of key marker genes across the annotated T cell subclusters.

F. Stacked bar plot showing the proportion of T cell subclusters across treatment groups, highlighting a relative expansion of effector memory T cells following combination therapy.

G, H. UMAP visualization (G) and violin plots (H) of the Tpe (progenitor exhausted) signature score, indicating an increase in this population with combination therapy.

I, J. UMAP visualization (I) and violin plots (J) of the Tte (terminally exhausted) signature score.

K. Gene set enrichment analysis (GSEA) plot showing that CD4$^+$ T cells from mice treated with combination therapy are enriched for pathways related to antigen processing and presentation compared with anti-PD-1 monotherapy.

L. UMAP visualizations of marker genes used to define CD4$^+$ T cell subsets.

M. Violin plots showing expression of key marker genes across CD4$^+$ effector T cell subsets.

N. UMAP visualization of the Tfh (T follicular helper) signature score across treatment groups.

O. Violin plot showing the Tfh signature score across different CD4$^+$ T cell subsets.

P, Q. Stacked bar plots showing the number (P) and proportion (Q) of CD4$^+$ Tfh-like cells across treatment groups, confirming their expansion with combination therapy.

R. UMAP visualization of T cells re-classified onto a reference atlas using the ProjecTILs algorithm.

S, T. Bar plots showing the number (S) and proportion (T) of CD8$^+$ T cells in each ProjecTILs-defined state, confirming a shift towards effector memory phenotypes with combination therapy.

U, V. Bar plots showing the number (U) and proportion (V) of CD4$^+$ T cells in each ProjecTILs-defined state, highlighting an increase in Tfh-like cells.

BrM, brain metastasis; GO, Gene Ontology; DEG, differentially expressed gene; GSEA, gene

set enrichment analysis; Tfh, T follicular helper; Tpe, progenitor exhausted T cell; Tte, terminally exhausted T cell; UMAP, Uniform Manifold Approximation and Projection.

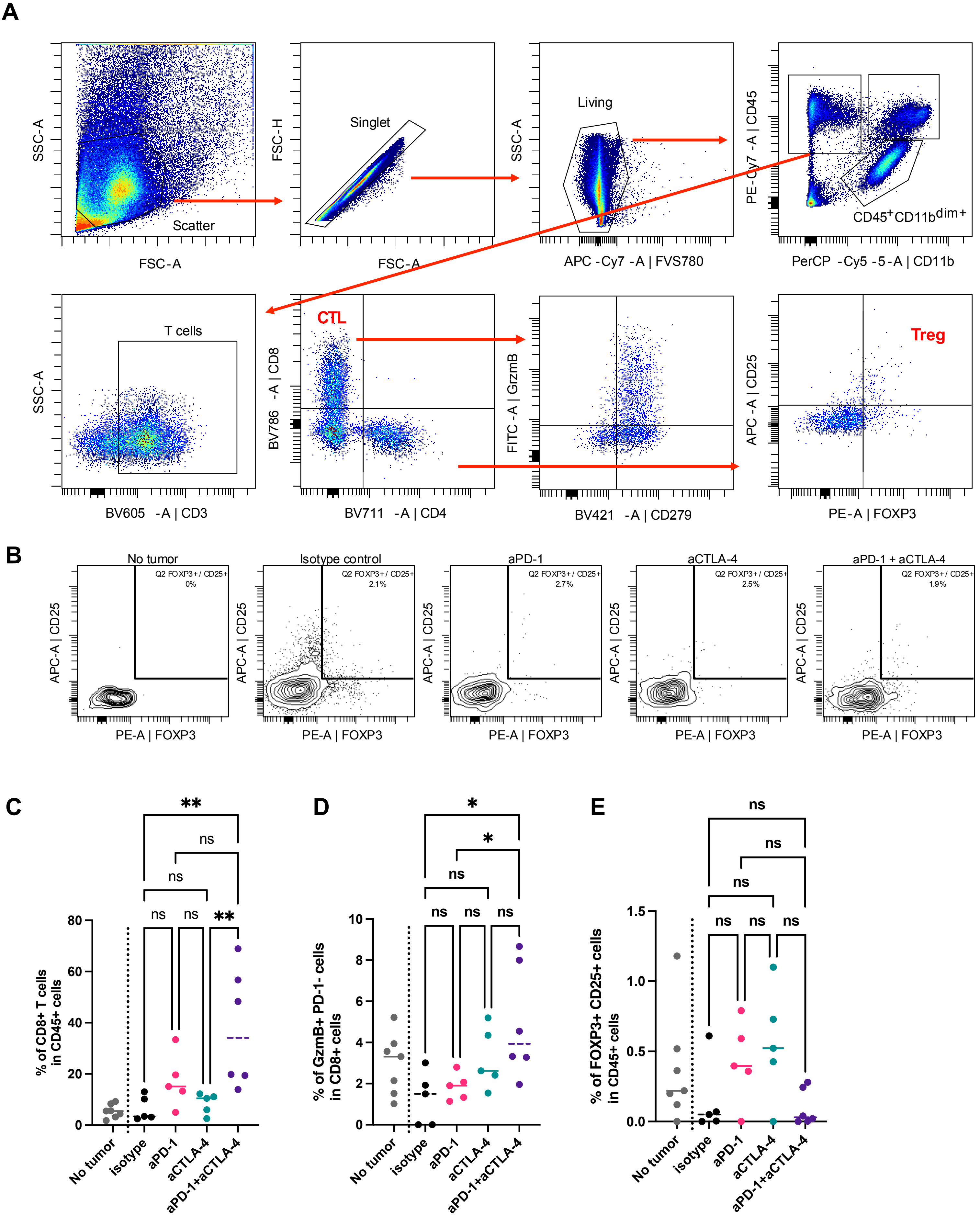
A
SSC-A
FSC-A
Scatter
FSC-H
Singlet
Living
APC -Cy7 -A | FVS780
PE-Cy7 -A | CD45
PerCP -Cy5 -5 -A | CD11b
CD45+CD11bdim+
T cells
BV605 -A | CD3
CTL
BV786 -A | CD8
BV711 -A | CD4
FITC -A | GrzmB
BV421 -A | CD279
Treg
APC -A | CD25
PE-A | FOXP3
B
No tumor
Isotype control
aPD-1
aCTLA-4
aPD-1 + aCTLA-4
Q2 FOXP3+ / CD25+
0%
2.1%
2.7%
2.5%
1.9%
APC-A | CD25
PE-A | FOXP3
C
% of CD8+ T cells in CD45+ cells
D
% of GzmB+ PD-1- cells in CD8+ cells
E
% of FOXP3+ CD25+ cells in CD45+ cells
No tumor
isotype
aPD-1
aCTLA-4
aPD-1+aCTLA-4
ns
*
**

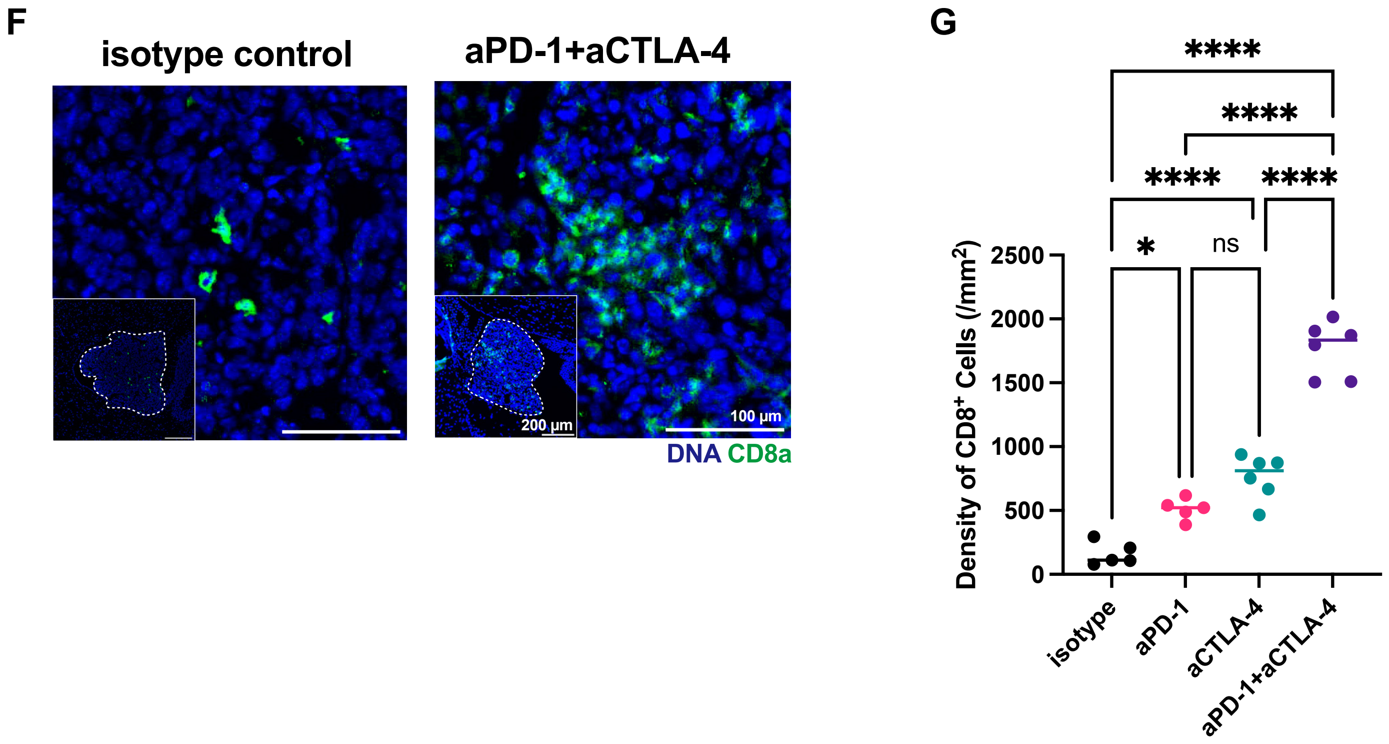
F
isotype control
aPD-1+aCTLA-4
200 µm
100 µm
DNA CD8a
G
****
****
****
****
*
ns
Density of CD8+ Cells (/mm2)
2500
2000
1500
1000
500
0
isotype
aPD-1
aCTLA-4
aPD-1+aCTLA-4

Supplementary Figure 7

A. Sequential gating strategy used for flow cytometric analysis of T cell subsets in BrM-bearing brain samples. After exclusion of debris and doublets, live $CD45^+$ immune cells were gated, followed by identification of $CD3^+$ T cells and $CD4^+$ or $CD8^+$ T cell subsets. This gating strategy corresponds to the quantification of $CD8^+$ T cells among $CD45^+$ cells shown in Figure 6B and Supplementary Figure 7C. The $CD8^+$ T cell gate was further used to identify functional $GzmB^+PD\text{-}1^-$ $CD8^+$ T cells, corresponding to Figure 6C and Supplementary Figure 7D. $CD4^+$ T cells were further gated to identify $FOXP3^+CD25^+$ Tregs, corresponding to Figure 6D and Supplementary Figure 7E.

B. Representative scatter plot showing the gating of regulatory T cells (Tregs; $FOXP3^+$, $CD25^+$) from the parent $CD4^+$ T cell population.

C. Flow cytometry analysis of the LLC-derived BrM model, showing that combination therapy significantly increased the percentage of $CD8^+$ T cells compared with the isotype control (mean ± SD; no tumor, n = 7; isotype, n = 5; aPD-1, n = 5; aCTLA-4, n = 5; aPD-1 + aCTLA-4, n = 6).

D. Flow cytometry analysis showing that combination therapy significantly increased the percentage of functional $GzmB^+PD\text{-}1^-$ cells within the $CD8^+$ T cell population in the LLC-derived BrM model (mean ± SD; no tumor, n = 7; isotype, n = 5; aPD-1, n = 5; aCTLA-4, n = 5; aPD-1 + aCTLA-4, n = 6).

E. Flow cytometry analysis of Treg cell percentage in $CD4^+$ T cells from LLC-derived BrM, showing no significant difference between treatment groups (mean ± SD; no tumor, n = 7; isotype, n = 5; aPD-1, n = 5; aCTLA-4, n = 5; aPD-1 + aCTLA-4, n = 6).

F. Representative immunofluorescence images of LLC-derived BrM, showing increased $CD8a^+$ T cell (green) infiltration in the combination therapy group compared with isotype control.

G. Quantification confirming a significantly higher density of $CD8a^+$ T cells in the combination therapy group. (mean ± SD; isotype, n = 5; aPD-1, n = 5; aCTLA-4, n = 5; aPD-1 + aCTLA-4, n = 6). Each dot represents one mouse (mean per mouse). Quantification was performed using 3 sections per mouse and tumor fields per section. Data are combined from 2 independent experiments.

ns, not significant; * $P < 0.05$, *** $P < 0.001$. BrM, brain metastasis; CTL; cytotoxic T lymphocyte; GzmB, Granzyme B; Treg, regulatory T cell.

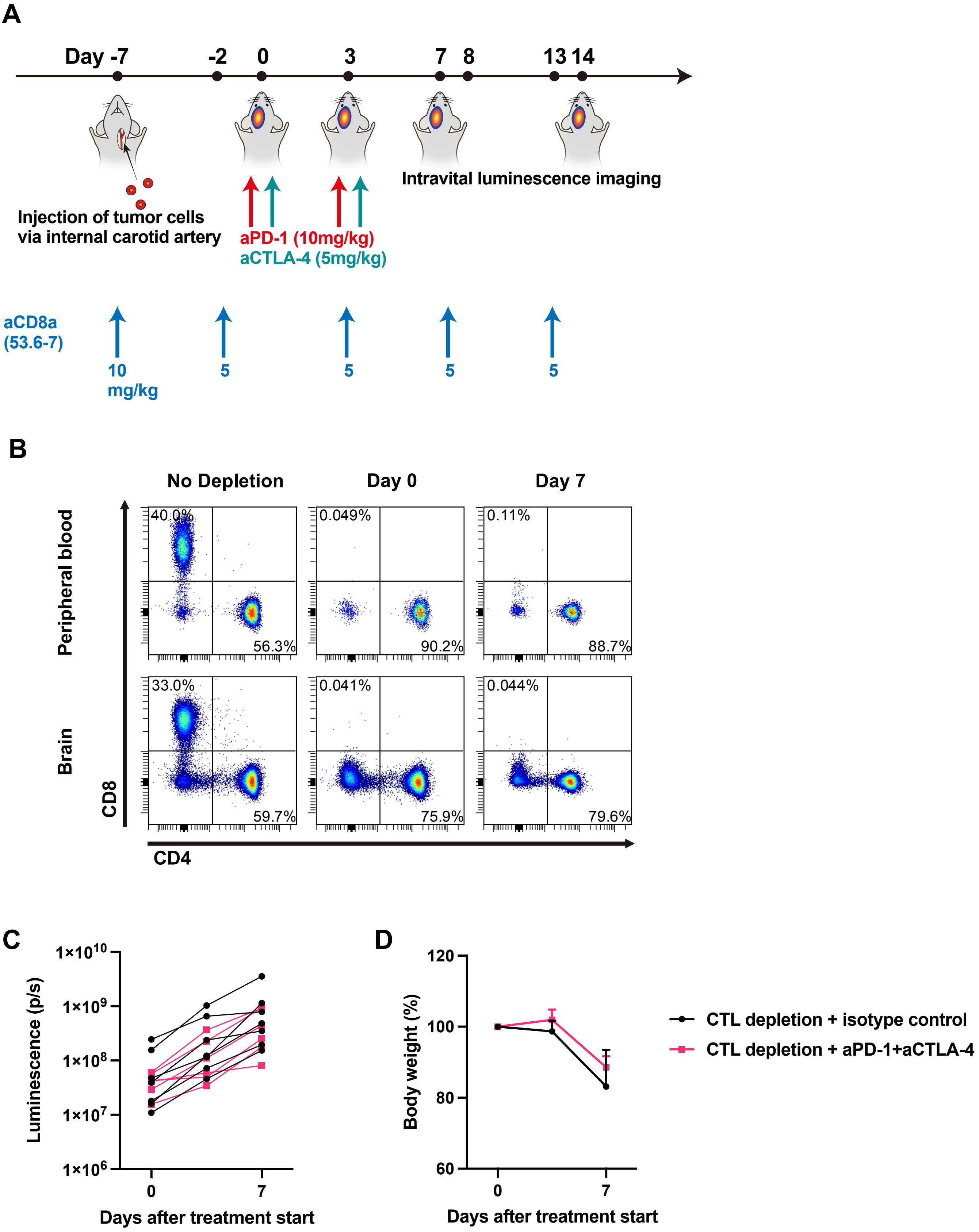
A
Day -7
-2
0
3
7
8
13
14
Injection of tumor cells via internal carotid artery
aPD-1 (10mg/kg)
aCTLA-4 (5mg/kg)
Intravital luminescence imaging
aCD8a (53.6-7)
10 mg/kg
5
5
5
5
B
No Depletion
Day 0
Day 7
Peripheral blood
Brain
CD8
CD4
40.0%
56.3%
0.049%
90.2%
0.11%
88.7%
33.0%
59.7%
0.041%
75.9%
0.044%
79.6%
C
Luminescence (p/s)
1×10^10
1×10^9
1×10^8
1×10^7
1×10^6
0
7
Days after treatment start
D
Body weight (%)
120
100
80
60
0
7
Days after treatment start
CTL depletion + isotype control
CTL depletion + aPD-1+aCTLA-4

E

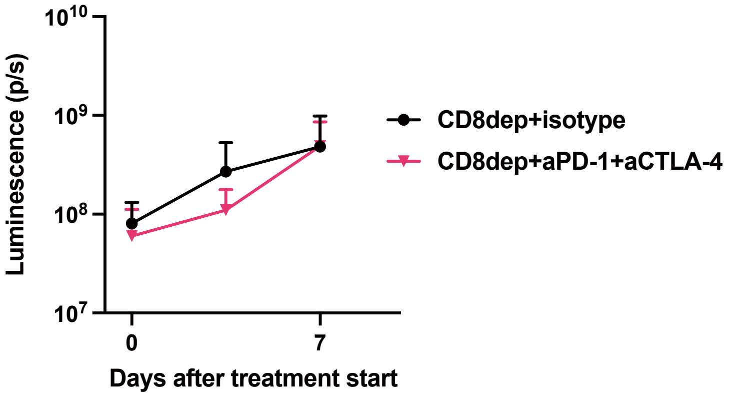

Luminescence (p/s)
10^{10}
10^{9}
10^{8}
10^{7}
0
7
Days after treatment start
CD8dep+isotype
CD8dep+aPD-1+aCTLA-4

F

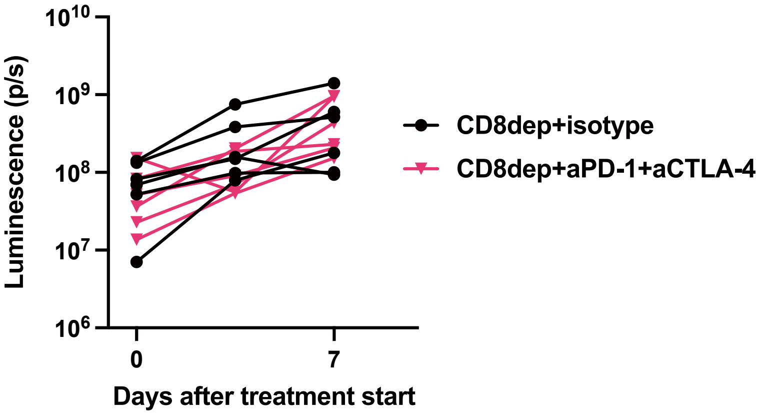

Luminescence (p/s)
10^{10}
10^{9}
10^{8}
10^{7}
10^{6}
0
7
Days after treatment start
CD8dep+isotype
CD8dep+aPD-1+aCTLA-4

G

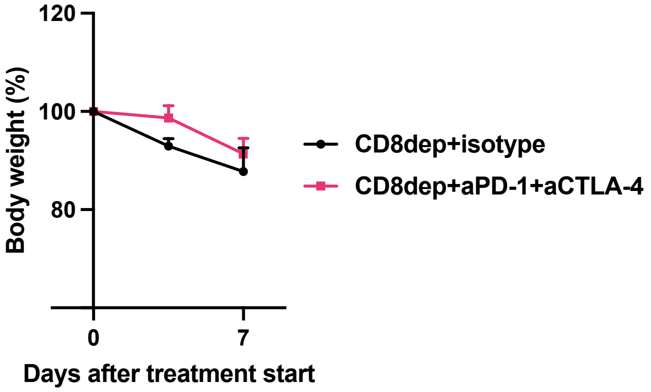

Body weight (%)
120
100
80
0
7
Days after treatment start
CD8dep+isotype
CD8dep+aPD-1+aCTLA-4

H

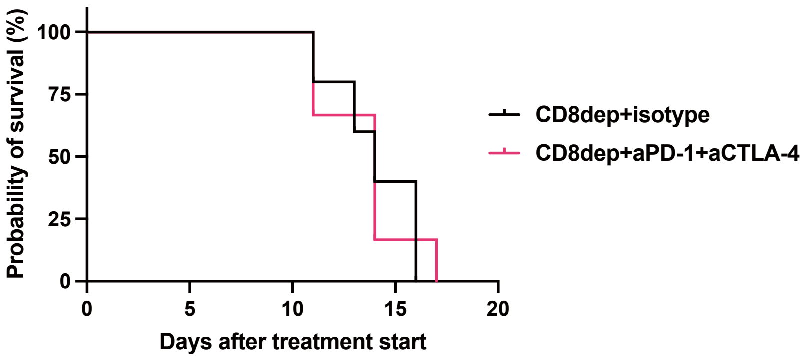

Probability of survival (%)
100
75
50
25
0
0
5
10
15
20
Days after treatment start
CD8dep+isotype
CD8dep+aPD-1+aCTLA-4

Supplementary Figure 8

A. Schematic representation of the cytotoxic T lymphocyte (CTL) depletion experiment schedule.

B. Flow cytometry analysis confirming successful depletion of $CD8^+$ T cells in peripheral blood and brain samples.

C. Longitudinal changes in luminescence for individual mice in the CMT167-derived BrM model during CTL depletion (CTL depletion + isotype, n = 7; CTL depletion + aPD-1 + aCTLA-4, n = 6).

D. Longitudinal body weight changes in the CMT167 model, normalized to baseline (mean ± SD; CTL depletion + isotype, n = 7; CTL depletion + aPD-1 + aCTLA-4, n = 6).

E, F. Longitudinal analysis of tumor burden in the LLC-derived BrM model following CTL depletion, showing the group mean (E, mean ± SD) and individual mouse trajectories (F) (n = 5 mice per group). The therapeutic benefit of combination therapy was abrogated.

G. Longitudinal body weight changes in the LLC model, showing no significant differences between treatment groups (mean ± SD; n = 5 mice per group).

H. Kaplan–Meier survival curves for the LLC model demonstrating that the survival benefit of combination therapy was lost with CTL depletion (n = 5 mice per group).

BrM, brain metastasis; CTL, cytotoxic T lymphocyte; SD, standard deviation.

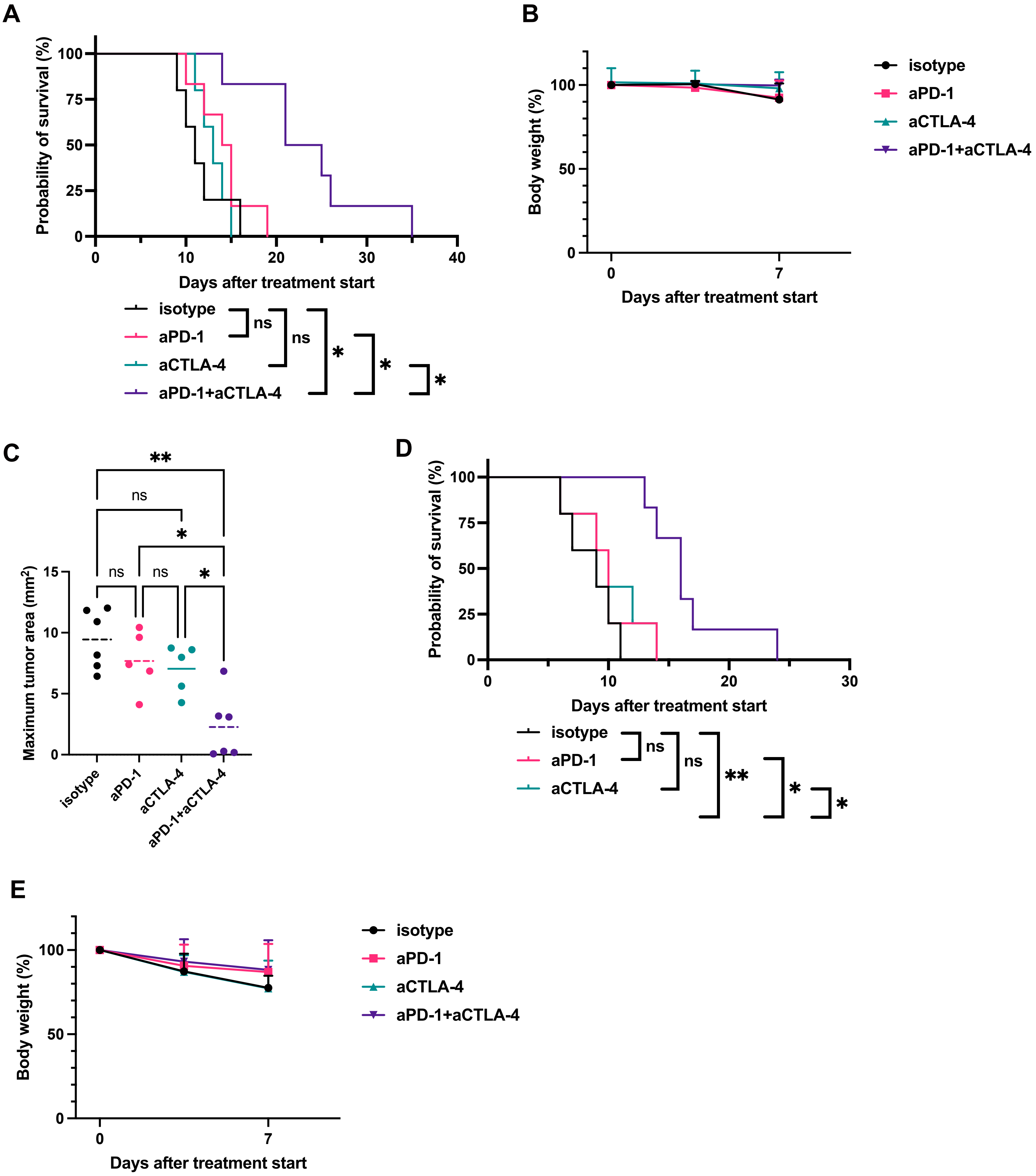
A
Probability of survival (%)
100
75
50
25
0
0
10
20
30
40
Days after treatment start
isotype
aPD-1
aCTLA-4
aPD-1+aCTLA-4
ns
ns
*
*
*
B
Body weight (%)
100
50
0
0
7
Days after treatment start
isotype
aPD-1
aCTLA-4
aPD-1+aCTLA-4
C
**
ns
*
ns
ns
*
Maximum tumor area (mm²)
15
10
5
0
isotype
aPD-1
aCTLA-4
aPD-1+aCTLA-4
D
Probability of survival (%)
100
75
50
25
0
0
10
20
30
Days after treatment start
isotype
aPD-1
aCTLA-4
ns
ns
**
*
*
E
Body weight (%)
100
50
0
0
7
Days after treatment start
isotype
aPD-1
aCTLA-4
aPD-1+aCTLA-4

Supplementary Figure 9

A, Kaplan–Meier survival curves demonstrating that combination therapy significantly prolonged survival in the intracranial implantation model using CMT167 cells (isotype, n = 6; aPD-1, n = 5; aCTLA-4, n = 5; aPD-1+aCTLA-4, n = 6).

B. Longitudinal body weight changes in the CMT167 intracranial implantation model, showing no significant treatment-related weight loss across groups (isotype, n = 6; aPD-1, n = 5; aCTLA-4, n = 5; aPD-1+aCTLA-4, n = 6. mean ± SD).

C. Quantification of intracranial tumor burden in the LLC intracranial implantation model, showing reduced tumor burden with combination therapy (nisotype, n = 6; aPD-1, n = 5; aCTLA-4, n = 5; aPD-1+aCTLA-4, n = 6. each dot represents an individual mouse).

D. Kaplan–Meier survival curves demonstrating that combination therapy significantly prolonged survival in the intracranial implantation model using LLC cells (isotype, n = 6; aPD-1, n = 5; aCTLA-4, n = 5; aPD-1+aCTLA-4, n = 6).

E. Longitudinal body weight changes in the LLC intracranial implantation model (isotype, n = 6; aPD-1, n = 5; aCTLA-4, n = 5; aPD-1+aCTLA-4, n = 6. mean ± SD).

SD, standard deviation.
