# Supplementary material for: Anti-PD-1 plus anti-CTLA-4 blockade overcomes immune exclusion in NSCLC brain metastases by enhancing CD8+ T cell responses and promoting tertiary lymphoid structure formation
Source: Nat Commun. 2026 Jul 7;17:5681. doi: 10.1038/s41467-026-74782-7 (PMC13342341; doi:10.1038/s41467-026-74782-7)
Supplement: Supplementary file 2 — Description of Additional Supplementary Files [file 41467_2026_74782_MOESM2_ESM.pdf]

Description of Additional Supplementary Files

Supplementary Data 1. Antibodies and reagents used in the study

Supplementary Data 2. Gene sets used in the study

Supplementary Data 3. Patient Characteristics (Nivolumab)

Supplementary Data 4. Patient Characteristics (Nivolumab+Ipilimumab)

Supplementary Data 5. Efficacy of nivolumab monotherapy or nivolumab + ipilimumab combination therapy

Supplementary Data 6. Intracranial efficacy of nivolumab monotherapy or nivolumab + ipilimumab combination therapy

Supplementary Data 7. Adverse events of nivolumab monotherapy or nivolumab + ipilimumab combination therapy

Supplementary Data 8. Patient characteristics of those who have paired specimens of primary lesion and brain metastasis of NSCLC.

Supplementary Data 9. Characteristics of TMA cohort patients stratified by cytotoxic T lymphocytes density

Supplementary Data 10. Characteristics of TMA cohort patients stratified by Treg density

Supplementary Data 11. Univariable and multivariable Cox regression analyses for post-resection overall survival in the BrM TMA cohort (CTL infiltration dichotomized at the median).

Supplementary Data 12. Univariable and multivariable Cox regression analyses for post-resection overall survival in the BrM TMA cohort (CTL infiltration as log2-transformed continuous variable)
