# Supplementary material for: Anti-PD-1 plus anti-CTLA-4 blockade overcomes immune exclusion in NSCLC brain metastases by enhancing CD8+ T cell responses and promoting tertiary lymphoid structure formation
Source: Nat Commun. 2026 Jul 7;17:5681. doi: 10.1038/s41467-026-74782-7 (PMC13342341; doi:10.1038/s41467-026-74782-7)
Supplement: Supplementary file 4 — Reporting Summary [file 41467_2026_74782_MOESM4_ESM.pdf]

nature portfolio

Corresponding author(s): Hiroaki Ozasa

Last updated by author(s): Mar 7, 2026

Reporting Summary

Nature Portfolio wishes to improve the reproducibility of the work that we publish. This form provides structure for consistency and transparency in reporting. For further information on Nature Portfolio policies, see our Editorial Policies and the Editorial Policy Checklist.

Statistics

For all statistical analyses, confirm that the following items are present in the figure legend, table legend, main text, or Methods section.

n/a	Confirmed	
☐	☒	The exact sample size (*n*) for each experimental group/condition, given as a discrete number and unit of measurement
☐	☒	A statement on whether measurements were taken from distinct samples or whether the same sample was measured repeatedly
☐	☒	The statistical test(s) used AND whether they are one- or two-sided<br>*Only common tests should be described solely by name; describe more complex techniques in the Methods section.*
☐	☒	A description of all covariates tested
☐	☒	A description of any assumptions or corrections, such as tests of normality and adjustment for multiple comparisons
☐	☒	A full description of the statistical parameters including central tendency (e.g. means) or other basic estimates (e.g. regression coefficient) AND variation (e.g. standard deviation) or associated estimates of uncertainty (e.g. confidence intervals)
☐	☒	For null hypothesis testing, the test statistic (e.g. *F*, *t*, *r*) with confidence intervals, effect sizes, degrees of freedom and *P* value noted<br>*Give P values as exact values whenever suitable.*
☒	☐	For Bayesian analysis, information on the choice of priors and Markov chain Monte Carlo settings
☒	☐	For hierarchical and complex designs, identification of the appropriate level for tests and full reporting of outcomes
☒	☐	Estimates of effect sizes (e.g. Cohen's *d*, Pearson's *r*), indicating how they were calculated

Our web collection on statistics for biologists contains articles on many of the points above.

Software and code

Policy information about availability of computer code

Data collection	No custom software was used for data collection.
Data analysis	No custom algorithms central to the research were developed. Analyses were performed using standard, publicly available software packages (e.g., R [version 4.4.2], Python [version 3.13.2] and packages, Seurat [5.1.0] for scRNA-seq, GraphPad Prism [10.4.2] for plotting/statistics, and ImageJ/Fiji [version 2.16.0] for image quantification), as specified in the Methods.

For manuscripts utilizing custom algorithms or software that are central to the research but not yet described in published literature, software must be made available to editors and reviewers. We strongly encourage code deposition in a community repository (e.g. GitHub). See the Nature Portfolio guidelines for submitting code & software for further information.

Data

Policy information about availability of data

All manuscripts must include a data availability statement. This statement should provide the following information, where applicable:

- Accession codes, unique identifiers, or web links for publicly available datasets
- A description of any restrictions on data availability
- For clinical datasets or third party data, please ensure that the statement adheres to our policy

Source Data are provided with this paper. Raw single-cell RNA sequencing data have been deposited in the DDBJ database [http://trace.ddbj.nig.ac.jp/DRASearch/experiment?acc=E-GEAD-1219], Accession No: E-GEAD-1219. All other data generated in this study are provided in the Supplementary Information, Supplementary Data and Source Data files.

Research involving human participants, their data, or biological material

Policy information about studies with human participants or human data. See also policy information about sex, gender (identity/presentation), and sexual orientation and race, ethnicity and racism.

Reporting on sex and gender	Sex (a biological attribute) was recorded from medical records as documented in the clinical chart. Sex was considered in the study design and is reported in the baseline characteristics tables (Supplementary Tables 3–4 and related tables). Gender identity/presentation was not collected in this retrospective cohort; therefore, gender-based analyses were not performed. Sex-stratified outcome analyses were not performed.
Reporting on race, ethnicity, or other socially relevant groupings	Race/ethnicity and other socially relevant groupings were not collected as part of routine clinical care for this retrospective cohort; therefore, these variables were not analyzed. The study population was derived from a single center in Japan, which should be considered when interpreting generalizability.
Population characteristics	This study included patients with non-small cell lung cancer (NSCLC) treated with immune checkpoint inhibitors and/or patients who underwent surgical resection of brain metastases, as described in the Methods. Covariate-relevant characteristics (e.g., age, sex, histology, treatment line/regimen, baseline brain metastasis status, prior brain-directed radiotherapy, and driver alteration status when available) are provided in Supplementary Tables 3–4 and related tables.
Recruitment	This was a single-center retrospective study. Eligible patients were identified from institutional medical records and clinical databases over the prespecified study periods described in the Methods. Because participants were not prospectively recruited, randomization was not performed; potential sources of bias include referral and selection bias inherent to retrospective single-center cohorts.
Ethics oversight	The study protocol was approved by the Kyoto University Graduate School and Faculty of Medicine Ethics Committee (Kyoto, Japan; certification numbers: R2163 and R2860). Given the retrospective nature of the study, the requirement for written informed consent was waived, in accordance with institutional and national guidelines. An opt-out procedure was implemented.

Note that full information on the approval of the study protocol must also be provided in the manuscript.

Field-specific reporting

Please select the one below that is the best fit for your research. If you are not sure, read the appropriate sections before making your selection.

- [x] Life sciences
- [ ] Behavioural & social sciences
- [ ] Ecological, evolutionary & environmental sciences

For a reference copy of the document with all sections, see nature.com/documents/nr-reporting-summary-flat.pdf

Life sciences study design

All studies must disclose on these points even when the disclosure is negative.

Sample size	Sample sizes were determined based on data availability for the retrospective clinical cohorts. For clinical and public transcriptomic analyses, all eligible samples meeting the predefined criteria were included. No formal statistical power calculation was performed a priori. No formal statistical method was used to predetermine sample size of in vivo experiments. Sample sizes were chosen based on previous studies using similar models, preliminary experiments and feasibility considerations, with sufficient biological replicates to assess reproducibility.
Data exclusions	For the retrospective clinical cohorts, patients with a history of multiple cancers or without radiographically measurable lesions were excluded, as described in the Methods. For response analyses, patients who received brain-directed radiotherapy within 30 days prior to ICI initiation were excluded to minimize potential confounding of intracranial response assessment; these patients were retained for time-to-event analyses (PFS/OS).<br>For mouse experiments, animals were excluded only if pre-established criteria were met (e.g., unsuccessful tumor establishment/technical failure of injection, or failure to meet predefined quality-control criteria for sample processing).
Replication	Key in vivo experiments were repeated in independent cohorts, and findings were reproduced using two independent syngeneic lung cancer cell lines (CMT167 and LLC), as described in the Results and Supplementary Information. Flow cytometry and immunofluorescence experiments were performed with independent biological replicates, and the number of independent experiments and biological replicates is stated in each figure legend. For scRNA-seq, samples were generated from pooled animals due to limited cell yield, as described in the Methods;
Randomization	For animal experiments, mice were allocated to treatment groups using random assignment.<br>Retrospective clinical analyses were observational; therefore, randomization was not applicable.
Blinding	Blinding was not performed for in vivo treatment allocation or outcome assessment due to the nature of the experiments; however, objective endpoints (e.g., bioluminescence imaging–based tumor burden, survival, and predefined flow cytometry gating strategies) were used to minimize bias. Pathologist-based scoring of TLS presence/density was performed by two pathologists in consensus.

Reporting for specific materials, systems and methods

We require information from authors about some types of materials, experimental systems and methods used in many studies. Here, indicate whether each material, system or method listed is relevant to your study. If you are not sure if a list item applies to your research, read the appropriate section before selecting a response.

Materials & experimental systems

n/a	Involved in the study
	x Antibodies
	x Eukaryotic cell lines
x	Palaeontology and archaeology
	x Animals and other organisms
	x Clinical data
x	Dual use research of concern
x	Plants

Methods

n/a	Involved in the study
x	ChIP-seq
	x Flow cytometry
x	MRI-based neuroimaging

Antibodies

Antibodies used	All antibodies used for immunohistochemistry, immunofluorescence, and flow cytometry are listed in Supplementary Table 1, including supplier, catalog number, clone name (where applicable), and working dilution/concentration. Therapeutic antibodies used for in vivo mouse experiments (anti–PD-1, anti–CTLA-4) are described in the Methods, including supplier, clone, dose, and dosing schedule.
Validation	Primary antibodies were used according to the manufacturers' datasheets for the indicated species and applications (IHC/IF/flow cytometry). Where available, antibodies were selected based on prior validation and widespread use in the literature and/or vendor-provided validation data. For key staining panels, expected staining patterns and appropriate negative controls (e.g., isotype controls for flow cytometry and omission of primary antibodies for immunostaining) were used to confirm specificity.

Eukaryotic cell lines

Policy information about cell lines and Sex and Gender in Research

Cell line source(s)	CMT167 (ECACC, 10032302) and LLC1 (ATCC, CRL-1642) mouse lung carcinoma cell lines were used. Stable reporter lines (mCherry and Akaluc luciferase) were generated from these parental lines. The sex of the donor animals for these cell lines was not specified by the suppliers.
Authentication	The cell lines were not authenticated (e.g., by STR profiling) in this study.
Mycoplasma contamination	All cell lines were routinely tested for mycoplasma contamination at least every 6 months using MycoCheck®, and tests were negative.
Commonly misidentified lines (See ICLAC register)	No commonly misidentified cell lines listed in the ICLAC register were used.

Animals and other research organisms

Policy information about studies involving animals; ARRIVE guidelines recommended for reporting animal research, and Sex and Gender in Research

Laboratory animals	Six-week-old male B6J (C57BL/6J ; Jackson Laboratory Japan, Inc., Yokohama, Japan; JAX stock no. 000664) and B6 Albino (B6N-Tyrc-Brd/BrdCrCrl ; Jackson Laboratory Japan, Inc., formerly Charles River Laboratories Japan, Yokohama, Japan) were purchased and used in this study. The B6 Albino strain was identified by the supplier strain name B6N-Tyrc-Brd/BrdCrCrl.
Wild animals	This study did not involve wild animals.
Reporting on sex	Only male mice were used in this study.
Field-collected samples	This study did not involve field-collected samples.
Ethics oversight	Mice were euthanized if they lost 20% of their baseline body weight or developed neurological symptoms. All animal experiments were approved by the Animal Research Committee at Kyoto University (IDs: MedKyo19594, MedKyo20258, MedKyo21274, MedKyo22254, MedKyo23211, and MedKyo24244) and were conducted according to ARRIVE guidelines.

Note that full information on the approval of the study protocol must also be provided in the manuscript.

Clinical data

Policy information about [clinical studies]

All manuscripts should comply with the ICMJE [guidelines for publication of clinical research] and a completed [CONSORT checklist] must be included with all submissions.

Clinical trial registration	Not applicable. This study was a retrospective single-center observational analysis and did not involve any clinical trial or interventional clinical study.
Study protocol	The study protocol was approved by the Kyoto University Graduate School and Faculty of Medicine Ethics Committee (certification numbers: R2163 and R2860). The study was conducted in accordance with the Declaration of Helsinki and Japanese ethical guidelines. Clinical information was retrospectively obtained from electronic medical records, and archived clinical specimens collected with comprehensive consent were used. Patients were given the opportunity to opt out.
Data collection	Clinical data were retrospectively collected from electronic medical records at Kyoto University Hospital. Archived formalin-fixed paraffin-embedded clinical specimens were used for tissue analyses. Patients who received nivolumab or nivolumab plus ipilimumab were identified according to the eligibility criteria described in the Methods.
Outcomes	Outcomes included progression-free survival, overall survival, systemic and intracranial tumor response according to RECIST v1.1, cumulative incidence of brain metastases, treatment-related adverse events graded according to CTCAE v5.0, and associations between immune cell infiltration in resected brain metastases and post-resection survival.

Plants

Seed stocks	Plants are not applicable to this study.
Novel plant genotypes	Plants are not applicable to this study.
Authentication	Plants are not applicable to this study.

Flow Cytometry

Plots

Confirm that:

- [x] The axis labels state the marker and fluorochrome used (e.g. CD4-FITC).
- [x] The axis scales are clearly visible. Include numbers along axes only for bottom left plot of group (a 'group' is an analysis of identical markers).
- [x] All plots are contour plots with outliers or pseudocolor plots.
- [x] A numerical value for number of cells or percentage (with statistics) is provided.

Methodology

Sample preparation	Single-cell suspensions were prepared from mouse BrM brain tissues by mechanical dissociation and enzymatic digestion with collagenase IV and DNase. Samples were filtered, debris was reduced using Percoll gradients, and red blood cells were lysed using a RBC lysis buffer. Cells were then stained for flow cytometry.
Instrument	Flow cytometry data were acquired on a BD LSRFortessa.
Software	Data were acquired using BD FACSDiva and analyzed using OMIQ.
Cell population abundance	Cell population abundances are reported as percentages of the relevant parent population, and individual biological replicates are shown as dot plots where applicable. Exact n and the number of independent experiments are provided in the figure legends.
Gating strategy	Gating was performed by sequential exclusion of debris (FSC/SSC), doublets, and dead cells, followed by gating on CD45+ immune cells and relevant lineage markers. Compensation was performed using single-stained controls. A figure exemplifying the gating strategy is provided in the Supplementary Information.

- [x] Tick this box to confirm that a figure exemplifying the gating strategy is provided in the Supplementary Information.
