# Supplementary material for: Anti-PD-1 plus anti-CTLA-4 blockade overcomes immune exclusion in NSCLC brain metastases by enhancing CD8+ T cell responses and promoting tertiary lymphoid structure formation
Source: Nat Commun. 2026 Jul 7;17:5681. doi: 10.1038/s41467-026-74782-7 (PMC13342341; doi:10.1038/s41467-026-74782-7)
Supplement: Supplementary file 5 — Transparent Peer Review File [file 41467_2026_74782_MOESM5_ESM.pdf]

nature portfolio

Peer Review File

Anti-PD-1 Plus Anti-CTLA-4 Blockade Overcomes Immune Exclusion in NSCLC Brain Metastases by Enhancing CD8+ T Cell Responses and Promoting Tertiary Lymphoid Structure Formation

Corresponding Author: Dr Hiroaki Ozasa

This file contains all reviewer reports in order by version, followed by all author rebuttals in order by version.

Version 0:

Reviewer comments:

Reviewer #1

(Remarks to the Author)
NSCLC has a high tendency for formation of CNS mets. Clinical use of anti pd1 monotherapy for un-radiated brain mets has modest efficacy. Combination anti pd1 and ctla4 shows better outcomes than pd1 alone or chemo in both melanoma and lung cancer brain metastasis patients. There has not been much actual brain tissue for evaluation in NSCLC patients but prior work suggests an immune excluded TME in brain mets compared to extracranial mets.
The authors performed a retrospective analysis of NSCLC patient outcomes who had received nivo or nivo + ipi with or without chemo. Patients were stratified by presence or absence of brain mets at baseline and survival was assessed. There was data on cumulative incidence of brain met development from the group that did not have brain mets at treatment initiation. There was a group of NSCLC patients with resected brain mets that were analyzed for IHC and tissue microarray. Intra-carotid injection of tumor was used to establish brain mets in mouse models and some mice had simultaneously established SC tumor deposits. Tail vein injections used for primary lung cancer establishment.
Patient outcome data—retrospective analyses:
Single agent PD1 treated patients did poorly. Intracranial ORR was similar between monotherapy and combination (14% vs 17%) but intracranial disease control rate was more than doubled in combination treated patients.
Paired analyses of transcriptomic profiles of primary NSCLC and brain mets from public data sets suggested brain mets had reduced CD8 T cells, cytotoxicity and Treg scares with downregulation of chemokines like CCL19 and CCL21. TLS signature scores were also lower in brain mets than in primary tumors. Tumor samples from patients were also analyzed with similar findings in brain mets compared to primary tumors. Mice with carcinomatous meningitis (tumors established through intra-carotid injection) survived longer with combination therapy and brain tissue analysis suggested good CTL infiltration.
Single cell RNA seq from brain tissue of mice treated with either single agent or combination therapy were analyzed and showed marked increase in T cell compartment in combo treated mice. Expansion of T follicular helper cells following combination therapy lead to B cell activation and TLS development and this is more robustly seen in specimens treated with anti CTLA4.
Overall this is excellent work and it is clear the authors did their best to perform a detailed analysis within the limitations of having few patient samples to analyze. All of the clinical data has the disadvantage of being retrospective in nature but is in keeping with known literature-especially in melanoma. This work adds to the field of knowledge of NSCLC and CNS metastases and the power of immunotherapy in this setting.
Specific feedback:
Results line 347: first word should be “To”
What are the next steps/future directions?

Reviewer #2

(Remarks to the Author)
This is an interesting study from a brain tumor group in Japan. To put this study in context, there is tremendous interest in the

cellular immune mechanisms by which immune checkpoint blockade (ICB) acts in selected microenvironments, including the brain. The current data suggests that extracranial response rates and intracranial responses rates are similar. However, dual ICB with CTLA-4 and PD-1 antibody blockade appear to induce more effective anti-tumor immune responses compared with PD-1 mAb blockade alone. But why dual ICB is more effective is not clear. Tertiary lymphoid structures (TLS) are associated with potent anti-tumor immune responses and clinical benefit. In this study, the group utilized retrospective data from non-small cell lung cancer patients in Japan treated with nivolumab monotherapy compared with nivolumab and ipilimumab. Analysis of paired tumor tissues (primary lung, brain metastasis) suggests that brain mets have markedly reduced CD8+ TIL accumulation and fewer TLS compared with the primary lung tumor. To understand the mechanisms of innate immune resistance to ICB, they developed a pre-clinical lung cancer brain mets model that recapitulates the immune-excluded phenotype. This brain mets model was not responsive to PD-1 mAb blockade, but dual PD-1+CTLA-4 mAb blockade prolonged survival, recapitulating the retrospective patient data. Pre-clinical brain mets treated with dual ICB had increased CD8+ T cell infiltration and single cell RNA-seq demonstrated that the tumor infiltrating subpopulation was enriched for effector memory and progenitor-exhausted features. The pre-clinical benefit was dependent on CD8 cells in this model. Multiplex IF indicates that CD8+ T cell infiltration was linked with TLS-like features in the brain metastasis immune microenvironment. The strengths of this study include the paired clinical tumor samples and the nice pre-clinical model that recapitulates the patient characteristics. The weaknesses are associated with the retrospective nature of the patient data and the inconsistent and heterogenous development of tumors with internal carotid artery delivery. Detailed comments are below:
1. Figure 1. In the clinical data, when was ICB started? It is not clear from the text. Was single agent nivolumab or nivolumab+ipilimumab begun at the same time in both retrospective studies? Was the ICB started upon discovery of the brain mets or during the extracranial involvement?
2. Figure 1 B, C. Did the authors directly compare the PFS of patients with BrM between nivolumab monotherapy and Nivo + Ipi combination therapy? This is a straightforward and clinically relevant comparison that could demonstrate whether the combination therapy (Nivo + Ipi) provides superior tumor-control efficacy compared to Nivo alone in lung BrM.
3. Line 360-362, Supplementary Table 6. According to Supplementary Table 6, the nivolumab group includes 22 evaluable cases with 7 CR/PR responses, while the nivolumab + ipilimumab group includes only 6 cases with 4 CR/PR responses. Although the authors claim a “notably” higher disease control rate with combination therapy, they should report the statistical significance of this difference (e.g., using Fisher’s exact test). Given the small sample size, the difference is unlikely to be statistically significant, so the authors should avoid using terms such as “notably” or “significantly” when describing these results.
4. Figure 3. For the link between T cell density, it is not clear how the Kaplan-Meier graph was constructed. Were the group dichotomized by the median CD8 TIL density? Was a multi-variate analysis done to evaluate whether this variable is independent?
5. Figure 3. CD3 and CD20 are not really the defining features of TLS. Were any other transcriptional features used to define TLS? In addition, it is not clear how the presence of TLS was linked to survival in the patient population, but it is stated as such.
6. Figure 4. The spatial heterogeneity of tumor cell development following internal carotid artery seems to suggest that many of these lung tumor develop in the CSF space. As such, this work actually seems like a pre-clinical model of leptomeningeal disease that is being treated with dual ICB. Can the authors comment on this feature of tumor cell delivery and why they did not use typical intracranial implant delivery of these tumor cells?
7. Figure 4. In the pre-clinical model, there does not look to be a significant survival difference between dual ICB and anti-PD-1.
8. Figure 5 and 6. The authors show that, compared to isotype control, aCTLA-4 + aPD-1 treatment increases $CD8^+$ TPE cells, which is interesting. However, did the authors compare aCTLA-4 + aPD-1 to aCTLA-4 or aPD-1 monotherapy? This analysis is important because the authors have demonstrated that dual blockade induces better tumor control compared to monotherapies; therefore, identifying which immune cell populations contribute to this difference would strengthen the mechanistic insight.
9. While the increase in $CD8^+$ T cells with aPD-1 + aCTLA-4 compared to isotype control is statistically significant, the comparison with aPD-1 monotherapy is not, despite an overall trend. The authors should acknowledge this high variability in the flow cytometry data rather than vaguely stating: “511 performed flow cytometry and immunofluorescence analyses of BrM brain tissues (Figure 6A). Flow cytometry confirmed a significant increase in the percentage of $CD8^+$ T cells among $CD45^+$ immune cells in the BrM microenvironment following combination therapy compared with isotype control or monotherapies.” Overall, a more precise description of the statistical results and variability would improve the accuracy of the interpretation.Figure 7. This is the only place in the paper where the authors actually did direct ic implantation of tumor cells. Was this accompanied by subcutaneous tumor cell injection simultaneously? They see evidence for B cell infiltration in these studies, but was there any clinical benefit with dual ICB in this model?

Reviewer #3

(Remarks to the Author)
I co-reviewed this manuscript with one of the reviewers who provided the listed reports. This is part of the Nature Communications initiative to facilitate training in peer review and to provide appropriate recognition for Early Career Researchers who co-review manuscripts.

Version 1:

Reviewer comments:

Reviewer #1

(Remarks to the Author)
Thank you for your revisions

Reviewer #2

(Remarks to the Author)
I do not have any additional comments and/or considerations on this submission.

Reviewer #3

(Remarks to the Author)
I co-reviewed this manuscript with one of the reviewers who provided the listed reports. This is part of the Nature Communications initiative to facilitate training in peer review and to provide appropriate recognition for Early Career Researchers who co-review manuscripts.

Open Access This Peer Review File is licensed under a Creative Commons Attribution 4.0 International License, which permits use, sharing, adaptation, distribution and reproduction in any medium or format, as long as you give appropriate credit to the original author(s) and the source, provide a link to the Creative Commons license, and indicate if changes were made.
In cases where reviewers are anonymous, credit should be given to 'Anonymous Referee' and the source.
The images or other third party material in this Peer Review File are included in the article’s Creative Commons license, unless indicated otherwise in a credit line to the material. If material is not included in the article’s Creative Commons license and your intended use is not permitted by statutory regulation or exceeds the permitted use, you will need to obtain permission directly from the copyright holder.
To view a copy of this license, visit https://creativecommons.org/licenses/by/4.0/

Response to Reviewers

We thank the reviewers for their constructive evaluation of our manuscript entitled "Anti-PD-1 Plus Anti-CTLA-4 Blockade Overcomes Immune Exclusion in NSCLC Brain Metastases by Enhancing $CD8^+$ T Cell Responses and Promoting Tertiary Lymphoid Structure Formation" (Manuscript ID: NCOMMS-25-74404-A).

The major revisions include: (i) direct comparison of PFS and OS between nivolumab monotherapy and nivolumab plus ipilimumab in patients with baseline brain metastases (R2-2); (ii) expanded TLS characterization with additional immunohistochemical markers (CD21, PNAd, BCL6, CCL21), and clarified the survival analysis by providing a more detailed description of the results (R2-5); (iii) clarification of the CD8 T-cell density survival analysis, including cutoff definition and Cox regression analyses (R2-4); (iv) detailed description of the internal carotid artery injection methodology and its rationale/limitations (R2-6); (v) strengthened controls and increased biological replicates for flow cytometry and immunofluorescence experiments, with per-mouse quantification (R2-8); (vi) revised statistical reporting, including Fisher's exact tests for intracranial response/disease control, explicit pairwise comparisons, and acknowledgment of variability (R2-3, R2-9); and (vii) addition of survival and tumor burden analyses in the intracranial implantation model (R2-10).A point-by-point response follows below.
All changes are indicated by tracked changes in the revised manuscript.

Data Availability for Review
Raw single-cell RNA sequencing data have been deposited in the DDBJ Genomic Expression Archive (GEA) under accession E-GEAD-1219. Reviewer access is available as follows:
Go to https://ddbj.nig.ac.jp/gea/reviewer
Enter accession number E-GEAD-1219 and the access token provided below.
Access token: E3ovm1adv6ENpvQPXWMw
Detailed instructions are available at: https://www.ddbj.nig.ac.jp/gea/reviewer-access-e.html

Response to Reviewer #1

Reviewer #1 (Remarks to the Author):

NSCLC has a high tendency for formation of CNS mets. Clinical use of anti pd1 monotherapy for un-radiated brain mets has modest efficacy. Combination anti pd1 and ctla4 shows better outcomes than pd1 alone or chemo in both melanoma and lung cancer brain metastasis patients. There has not been much actual brain tissue for evaluation in NSCLC patients but prior work suggests an immune excluded TME in brain mets compared to extracranial mets. The authors performed a retrospective analysis of NSCLC patient outcomes who had received nivo or nivo + ipi with or without chemo. Patients were stratified by presence or absence of brain mets at baseline and survival was assessed. There was data on cumulative incidence of brain met development from the group that did not have brain mets at treatment initiation. There was a group of NSCLC patients with resected brain mets that were analyzed for IHC and tissue microarray. Intra-carotid injection of tumor was used to establish brain mets in mouse models and some mice had simultaneously established SC tumor deposits. Tail vein injections used for primary lung cancer establishment.

Patient outcome data—retrospective analyses:

Single agent PD1 treated patients did poorly. Intracranial ORR was similar between monotherapy and combination (14% vs 17%) but intracranial disease control rate was more than doubled in combination treated patients.

Paired analyses of transcriptomic profiles of primary NSCLC and brain mets from public data sets suggested brain mets had reduced CD8 T cells, cytotoxicity and Treg scares with downregulation of chemokines like CCL19 and CCL21. TLS signature scores were also lower in brain mets than in primary tumors. Tumor samples from patients were also analyzed with similar findings in brain mets compared to primary tumors. Mice with carcinomatous meningitis (tumors established through intra-carotid injection) survived longer with combination therapy and brain tissue analysis suggested good CTL infiltration.

Single cell RNA seq from brain tissue of mice treated with either single agent or combination therapy were analyzed and showed marked increase in T cell compartment in combo treated mice. Expansion of T follicular helper cells following combination therapy lead to B cell activation and TLS development and this is more robustly seen in specimens treated with anti CTLA4.

Overall this is excellent work and it is clear the authors did their best to perform a detailed analysis within the limitations of having few patient samples to analyze. All of the clinical data has the disadvantage of being retrospective in nature but is in keeping with known literature-especially in melanoma. This work adds to the field of knowledge of NSCLC and CNS metastases and the power of immunotherapy in this setting.

Specific feedback:

Results line 347: first word should be “To”

What are the next steps/future directions?

Response:

We thank the reviewer for the positive evaluation and helpful suggestions. We address the specific points below.

Comment R1-1: “Results line 347: first word should be ‘To’”

Response:

We corrected the typographical error as suggested.

Changes made: Results, page 20, line 563.

Comment R1-2: “What are the next steps/future directions?”

Response:

We thank the reviewer for this constructive suggestion. We have added a brief “Future directions” statement to the final paragraph of the Discussion. In this revision, we highlight several next steps: (i) prospective clinical evaluation of PD-1/CTLA-4 combination immunotherapy in NSCLC patients with brain metastases; (ii) mechanistic dissection of immune cell trafficking into BrM to better understand and overcome immune exclusion; and (iii) exploration of additional immunotherapy backbones and rational combinations beyond PD-1/CTLA-4 blockade, including strategies aimed at promoting TLS formation/maturation to enhance local antitumor immunity in BrM.

Changes made: Discussion, page 29, lines 844–850.

Response to Reviewer #2

Reviewer #2 (Remarks to the Author):

This is an interesting study from a brain tumor group in Japan. To put this study in context, there is tremendous interest in the cellular immune mechanisms by which immune checkpoint blockade (ICB) acts in selected microenvironments, including the brain. The current data suggests that extracranial response rates and intracranial responses rates are similar. However, dual ICB with CTLA-4 and PD-1 antibody blockade appear to induce more effective anti-tumor immune responses compared with PD-1 mAb blockade alone. But why dual ICB is more effective is not clear. Tertiary lymphoid structures (TLS) are associated with potent anti-tumor immune responses and clinical benefit. In this study, the group utilized retrospective data from non-small cell lung cancer patients in Japan treated with nivolumab monotherapy compared with nivolumab and ipilimumab. Analysis of paired tumor tissues (primary lung, brain metastasis) suggests that brain mets have markedly reduced CD8+ TIL accumulation and fewer TLS compared with the primary lung tumor. To understand the mechanisms of innate immune resistance to ICB, they developed a pre-clinical lung cancer brain mets model that recapitulates the immune-excluded phenotype. This brain mets model was not responsive to PD-1 mAb blockade, but dual PD-1+CTLA-4 mAb blockade prolonged survival, recapitulating the retrospective patient data. Pre-clinical brain mets treated with dual ICB had increased CD8+ T cell infiltration and single cell RNA-seq demonstrated that the tumor infiltrating subpopulation was enriched for effector memory and progenitor-exhausted features. The pre-clinical benefit was dependent on CD8 cells in this model. Multiplex IF indicates that CD8+ T cell infiltration was linked with TLS-like features in the brain metastasis immune microenvironment. The strengths of this study include the paired clinical tumor samples and the nice pre-clinical model that recapitulates the patient characteristics. The weaknesses are associated with the retrospective nature of the patient data and the inconsistent and heterogenous development of tumors with internal carotid artery delivery. Detailed comments are below:

1. Figure 1. In the clinical data, when was ICB started? It is not clear from the text. Was single agent nivolumab or nivolumab+ipilimumab begun at the same time in both retrospective studies? Was the ICB started upon discovery of the brain mets or during the extracranial involvement?

2. Figure 1 B, C. Did the authors directly compare the PFS of patients with BrM between nivolumab monotherapy and Nivo + Ipi combination therapy? This is a straightforward and clinically relevant comparison that could demonstrate whether the combination therapy (Nivo + Ipi) provides superior tumor-control efficacy compared to Nivo alone in lung BrM.

3. Line 360-362, Supplementary Table 6. According to Supplementary Table 6, the nivolumab

group includes 22 evaluable cases with 7 CR/PR responses, while the nivolumab + ipilimumab group includes only 6 cases with 4 CR/PR responses. Although the authors claim a “notably” higher disease control rate with combination therapy, they should report the statistical significance of this difference (e.g., using Fisher’s exact test). Given the small sample size, the difference is unlikely to be statistically significant, so the authors should avoid using terms such as “notably” or “significantly” when describing these results.

4. Figure 3. For the link between T cell density, it is not clear how the Kaplan-Meier graph was constructed. Were the group dichotomized by the median CD8 TIL density? Was a multi-variate analysis done to evaluate whether this variable is independent?

5. Figure 3. CD3 and CD20 are not really the defining features of TLS. Were any other transcriptional features used to define TLS? In addition, it is not clear how the presence of TLS was linked to survival in the patient population, but it is stated as such.

6. Figure 4. The spatial heterogeneity of tumor cell development following internal carotid artery seems to suggest that many of these lung tumor develop in the CSF space. As such, this work actually seems like a pre-clinical model of leptomeningeal disease that is being treated with dual ICB. Can the authors comment on this feature of tumor cell delivery and why they did not use typical intracranial implant delivery of these tumor cells?

7. Figure 4. In the pre-clinical model, there does not look to be a significant survival difference between dual ICB and anti-PD-1.

8. Figure 5 and 6. The authors show that, compared to isotype control, aCTLA-4 + aPD-1 treatment increases CD8$^+$ TPE cells, which is interesting. However, did the authors compare aCTLA-4 + aPD-1 to aCTLA-4 or aPD-1 monotherapy? This analysis is important because the authors have demonstrated that dual blockade induces better tumor control compared to monotherapies; therefore, identifying which immune cell populations contribute to this difference would strengthen the mechanistic insight.

9. While the increase in CD8$^+$ T cells with aPD-1 + aCTLA-4 compared to isotype control is statistically significant, the comparison with aPD-1 monotherapy is not, despite an overall trend. The authors should acknowledge this high variability in the flow cytometry data rather than vaguely stating: “511 performed flow cytometry and immunofluorescence analyses of BrM brain tissues (Figure 6A). Flow cytometry confirmed a significant increase in the percentage of CD8$^+$ T cells among CD45$^+$ immune cells in the BrM microenvironment following combination therapy compared with isotype control or monotherapies.” Overall, a more precise description of the statistical results and variability would improve the accuracy of the interpretation.

Figure 7. This is the only place in the paper where the authors actually did direct ic implantation of tumor cells. Was this accompanied by subcutaneous tumor cell injection

simultaneously? They see evidence for B cell infiltration in these studies, but was there any clinical benefit with dual ICB in this model?

Response:

We thank the reviewer for the thorough and constructive critique. We have revised the manuscript to clarify the clinical cohort definitions and timing of ICB initiation, strengthen TLS definitions using expanded histological features, add survival analyses linked to TLS, and improve the rigor of the preclinical methodology and immune-phenotyping controls, including monotherapy comparisons and explicit reporting of variability and statistical outcomes.

Comment R2-1: "Figure 1. In the clinical data, when was ICB started? It is not clear from the text. Was single agent nivolumab or nivolumab+ipilimumab begun at the same time in both retrospective studies? Was the ICB started upon discovery of the brain mets or during the extracranial involvement?"

Response:

We thank the reviewer for this important question. We agree that the timing of ICI initiation and the definition of baseline brain metastasis status were not sufficiently clear in the original submission. We have therefore revised both the Methods and Results to explicitly define the index date as the date of ICI initiation and to clarify how "baseline BrM" was determined.

Specifically, we now state that patients were stratified by the presence or absence of radiographically confirmed brain metastases at the time of ICI initiation, based on review of imaging obtained between NSCLC diagnosis and ICI initiation. We also clarified that brain-directed radiotherapy prior to ICI initiation was focal irradiation only, and we specified our analytic handling of recent radiotherapy (exclusion of patients receiving brain radiotherapy within 30 days prior to the index date for response analyses, while retaining these patients for PFS/OS analyses) to minimize confounding of intracranial response assessment.

In addition, to improve interpretability, we revised the Results text to report median PFS, hazard ratios, and p-values for the key Kaplan–Meier comparisons.

Changes made: Methods revised to define the index date, intracranial response assessment approach, and baseline BrM definition (page 6–7, lines 127–138); Results revised to clarify stratification at ICI initiation and to include median PFS, HRs, and p-values for the primary comparisons (page 16, lines 423–441).

Comment R2-2: "Figure 1 B, C. Did the authors directly compare the PFS of patients with BrM between nivolumab monotherapy and Nivo + Ipi combination therapy? This is a straightforward and clinically relevant comparison that could demonstrate

whether the combination therapy (Nivo + Ipi) provides superior tumor-control efficacy compared to Nivo alone in lung BrM."

Response:

We thank the reviewer for this important and clinically relevant suggestion. We have now performed a direct comparison of PFS between nivolumab monotherapy and nivolumab plus ipilimumab among patients with brain metastases at baseline. The results are presented in Supplementary Figure 1C–H and are described in the revised Results section.

We also provide the corresponding OS comparisons and an exploratory analysis excluding patients with driver oncogene alterations, which suggests that oncogenic driver status may influence outcomes in this retrospective cohort. Importantly, because the treatment cohorts differ substantially in baseline clinical context (including treatment line, driver alterations, and PD-L1 status), these comparisons are observational and should not be interpreted as evidence of a causal regimen effect.

Changes made: Results (page 16–17, lines 443–456); Discussion (page 26–27, lines 758–769). Supplementary Figure 1C–H added (legend: page 37–38, lines 1088–1105).

Comment R2-3: "Line 360-362, Supplementary Table 6. According to Supplementary Table 6, the nivolumab group includes 22 evaluable cases with 7 CR/PR responses, while the nivolumab + ipilimumab group includes only 6 cases with 4 CR/PR responses. Although the authors claim a "notably" higher disease control rate with combination therapy, they should report the statistical significance of this difference (e.g., using Fisher's exact test). Given the small sample size, the difference is unlikely to be statistically significant, so the authors should avoid using terms such as "notably" or "significantly" when describing these results."

Response:

We agree that our prior wording overstated the difference in intracranial disease control and that statistical testing should be provided. We have therefore performed Fisher's exact tests for intracranial response and disease control comparisons and updated the Results text to reflect statistical significance appropriately, avoiding terms such as "notably" or "significantly" when differences were not statistically significant. The corresponding p-values have been added to Supplementary Table 5-6 and stated in the manuscript.

In addition, prompted by this comment, we expanded statistical reporting for treatment-related adverse events to ensure consistent interpretation across efficacy and safety outcomes. Because grade ≥3 treatment-related adverse events occurred significantly more frequently in the combination group, we also revised the concluding statement in the Results to explicitly acknowledge the increased risk of high-grade adverse events when interpreting

potential intracranial efficacy.
Finally, for the cumulative incidence analysis of new BrM development in patients without baseline BrM, we have included the statistical result that was shown in the figure within the main text (Results), so that the presence or absence of statistical significance is transparent to the reader.

Changes made: Results revised (page 17, lines 457–473): Fisher's exact test p-values added for intracranial response and disease control comparisons; "notably" removed; cumulative incidence p-value stated in main text; grade ≥ 3 adverse event comparison with p-value added; concluding statement revised to acknowledge increased risk of high-grade adverse events. Supplementary Tables 5–6 updated with p-values; Supplementary Table 7 updated.

Comment R2-4: "For the link between T cell density, it is not clear how the Kaplan-Meier graph was constructed. Were the group dichotomized by the median CD8 TIL density? Was a multi-variate analysis done to evaluate whether this variable is independent?"

Response:

We thank the reviewer for this important and constructive comment. The Kaplan–Meier analysis in Figure 3E was constructed by dichotomizing CTL (CD8a+) density in BrM specimens into high- and low-infiltration groups using the cohort median as the cutoff. We have clarified this procedure in the Methods.
In addition, to address whether CTL density is independently associated with post-resection survival, we performed Cox proportional hazards regression analyses. CTL density remained significantly associated with post-resection overall survival in univariable and multivariable Cox models, and this finding was consistent when CTL density was modeled as a log2-transformed continuous variable (log2[CD8 density + 1]) in sensitivity analyses. The multivariable model included clinically relevant covariates (driver alteration status, preoperative steroid use, and completeness of resection) to limit model complexity given the sample size. We have added these details to the Methods and present the results in Supplementary Table 11 (dichotomized model) and Supplementary Table 12 (continuous model).

Changes made: Methods revised to specify median dichotomization and Cox modeling strategy (page 7, lines 156–165); Results revised to report univariable/multivariable and continuous-variable sensitivity analyses (page 19, lines 528–531); Supplementary Tables 11 and 12 added.

Comment R2-5: “Figure 3. CD3 and CD20 are not really the defining features of TLS. Were any other transcriptional features used to define TLS? In addition, it is not clear how the presence of TLS was linked to survival in the patient population, but it is stated as such.”

Response:

We thank the reviewer for this important comment. In response, we revised methods to clearly define the TLS quantification strategy and expanded our TLS characterization beyond CD3 and CD20 by adding immunohistochemical staining for additional established TLS-associated markers, including CD21 (follicular dendritic cell marker), PNAd (high endothelial venule marker), BCL6 (germinal center/activated B cell marker), and CCL21 (TLS-associated chemokine identified in our transcriptomic analyses). These data are presented in Figure 3F-G and Supplementary Figure 3E, and the corresponding antibody information has been added to Supplementary Table 1.

Second, regarding the link between TLS and survival, we clarified in the Results and Methods how TLS status was assessed and how survival was analyzed. Specifically, TLS presence in BrM specimens was evaluated on histological sections and patients were compared by Kaplan–Meier analysis (Supplementary Figure 3G). In our cohort, TLS presence in BrMs was not significantly associated with post-resection overall survival and did not show a clear trend toward improved outcome. We also added a more explicit discussion of limitations: section-level assessment may underestimate TLS positivity, and evaluation of the tumor periphery may be limited by the constraints of brain metastasis surgery, which prioritizes minimizing injury to adjacent normal brain tissue. These factors resulted in a small number of TLS-positive BrM cases and may have limited statistical power to detect a survival association.

Changes made: Figure 3F–G and Supplementary Figure 3E updated with additional TLS markers (CD21, PNAd, BCL6, CCL21); Supplementary Table 1 updated with antibody details; Methods revised to describe TLS quantification (page 9, lines 229–239); Results revised to describe expanded TLS characterization and survival analysis (page 19–20, lines 539–554); Supplementary Figure 3G legend updated (page 39, lines 1143–1144).

Comment R2-6: “Figure 4. The spatial heterogeneity of tumor cell development following internal carotid artery seems to suggest that many of these lung tumor develop in the CSF space. As such, this work actually seems like a pre-clinical model of leptomeningeal disease that is being treated with dual ICB. Can the authors comment on this feature of tumor cell delivery and why they did not use typical intracranial implant delivery of these tumor cells?”

Response:

We thank the reviewer for this insightful comment. We agree that ICA delivery can result in heterogeneous intracranial tumor distribution, including meningeal/CSF-space involvement. We have now clarified this point in the revised manuscript and explicitly acknowledge it as a limitation of ICA-based delivery models.

We primarily used the ICA injection model because it (i) mimics hematogenous dissemination to the brain, (ii) avoids confounding tissue injury caused by direct intracranial injection into the parenchyma/skull, and (iii) allows longitudinal quantification of intracranial tumor burden in an immunocompetent setting. In contrast, direct intracranial implantation can facilitate controlled parenchymal tumor growth but inevitably introduces local mechanical injury that may affect immune-cell recruitment and treatment response. Other approaches (e.g., intracardiac injection with brain-tropic selection) can generate widespread extracranial metastases, which may obscure intracranial-specific outcomes and survival endpoints driven by CNS disease.

Importantly, although ICA delivery consistently produced meningeal involvement in our model, we quantitatively assessed treatment effects on tumor burden using immunofluorescence-based tumor area measurements, and we observed that dual anti–PD-1 plus anti–CTLA-4 therapy suppressed both parenchymal lesions and meningeal tumor burden. Moreover, we complemented the ICA model with a direct intracranial implantation model in Fig. 7, which supported the therapeutic activity of combined PD-1/CTLA-4 blockade, thereby strengthening the generalizability of our conclusions across model systems. We have revised the Methods/Results/Discussion to better explain the model characteristics and to explicitly discuss this limitation.

Changes made: Methods (page 11, lines 291–293); Results (page 20–21, lines 580–585); Discussion (page 28, lines 818–824).

Comment R2-7: "Figure 4. In the pre-clinical model, there does not look to be a significant survival difference between dual ICB and anti-PD-1."

Response:

We thank the reviewer for this important comment. We agree that, in the CMT167 ICA BrM model (Fig. 4H), the survival difference between dual anti–PD-1/anti–CTLA-4 therapy and anti–PD-1 monotherapy does not reach statistical significance, and that our previous wording could be interpreted as overstating this comparison. We have therefore revised the Results text to explicitly state that the dual vs anti–PD-1 survival comparison is not significant in the CMT167 ICA model, while maintaining the conclusion that dual therapy improves survival compared with isotype control.

Importantly, we also note that in an independent ICA BrM model using LLC cells, dual therapy prolonged survival compared with anti–PD-1 monotherapy (Supplementary Fig. 4I), supporting reproducibility across model systems. In addition, we added a limitation statement in the Discussion acknowledging that the magnitude of survival benefit between active treatment arms may vary by model and that this comparison was not significant in the CMT167 ICA model.

Changes made: Results revised to clarify the statistical interpretation of the dual vs anti-PD-1 survival comparison in the CMT167 model and to reference the LLC replication (page 20, lines 575–576). Discussion updated to note this limitation (page 28, lines 825–830).

Comment R2-8: “Figure 5 and 6. The authors show that, compared to isotype control, aCTLA-4 + aPD-1 treatment increases $CD8^+$ TPE cells, which is interesting. However, did the authors compare aCTLA-4 + aPD-1 to aCTLA-4 or aPD-1 monotherapy? This analysis is important because the authors have demonstrated that dual blockade induces better tumor control compared to monotherapies; therefore, identifying which immune cell populations contribute to this difference would strengthen the mechanistic insight.”

Response:

We thank the reviewer for this important suggestion. We agree that direct comparisons to each monotherapy are critical to clarify which immune cell populations underlie the superior activity of dual checkpoint blockade. In the revision, we performed additional experiments and expanded the flow cytometry and immunofluorescence datasets in Fig. 6 and Supplementary Fig. 7 to include sufficient tumor free controls and adequately powered monotherapy arms (anti-PD-1 alone and anti-CTLA-4 alone), enabling more informative comparisons across treatment conditions.

Specifically, in the scRNA-seq analyses (Fig. 5 and Supplementary Fig. 6), we now describe treatment-associated shifts across the four conditions and highlight complementary tendencies observed with each agent: PD-1 blockade was associated with enrichment of $CD8^+$ effector-memory-like programs, consistent with qualitative changes in $CD8^+$ T-cell functionality, whereas CTLA-4 blockade was associated with increased Tfh-like representation within the $CD4^+$ compartment, suggesting preferential modulation of $CD4^+$ T-cell states. We also added a clarification that scRNA-seq libraries were generated from pooled samples due to limited immune cell yield from BrM-bearing brains, and thus these analyses are interpreted as descriptive trends rather than formal statistical inference across biological replicates.

In the flow cytometry analyses, we revised the flow cytometry and immunofluorescence section (Fig. 6) to report pairwise comparisons more precisely. We confirm robust differences

versus isotype control (e.g., increased CD8$^+$ frequency and cytotoxic features), while noting that comparisons between combination therapy and anti-PD-1 or anti-CTLA-4 monotherapy were not consistently statistically significant, despite an overall trend.

Collectively, these revisions address the reviewer's request by clarifying how each monotherapy relates to the combined phenotype and by framing the mechanistic interpretation in accordance with the strength of the data.

Changes made: Figure 6B–D, G revised to include expanded monotherapy and control cohorts (legend: page 35–36, lines 1013–1051); Figure 6E–F added as representative gating plots (page 36, lines 1031–1034). Supplementary Figure 7C–G updated to include monotherapy controls (page 43–44, lines 1276–1307). Results revised: scRNA-seq section describes treatment-associated shifts with complementary tendencies of each agent (page 22, lines 616–634 and page 23, lines 653–658); flow cytometry section reports pairwise comparisons explicitly (page 23–24, lines 660–692).

Comment R2-9: "While the increase in CD8$^+$ T cells with aPD-1 + aCTLA-4 compared to isotype control is statistically significant, the comparison with aPD-1 monotherapy is not, despite an overall trend. The authors should acknowledge this high variability in the flow cytometry data rather than vaguely stating: "511 performed flow cytometry and immunofluorescence analyses of BrM brain tissues (Figure 6A). Flow cytometry confirmed a significant increase in the percentage of CD8$^+$ T cells among CD45$^+$ immune cells in the BrM microenvironment following combination therapy compared with isotype control or monotherapies." Overall, a more precise description of the statistical results and variability would improve the accuracy of the interpretation."

Response:

We thank the reviewer for pointing this out. We agree that our original wording did not sufficiently distinguish statistically significant differences from directional trends and did not adequately acknowledge variability in the flow cytometry datasets. In the revised manuscript (Fig. 6 and Supplementary Fig. 7), we now report pairwise comparisons explicitly: the increase in CD8$^+$ T cells with combination therapy versus isotype control is statistically significant, whereas the comparison versus anti-PD-1 monotherapy shows a trend that does not consistently reach statistical significance, reflecting inter-animal variability. We have revised the Results text accordingly to ensure an accurate, statistically grounded interpretation.

Changes made: Results section "Combination therapy increases CTL infiltration and functionality in BrMs" revised (page 23–24, lines 660–692): pairwise comparisons reported explicitly; combination vs isotype control described as statistically significant; combination vs

anti-PD-1 monotherapy described as a trend not consistently reaching statistical significance, with inter-animal variability acknowledged.

Comment R2-10: “Figure 7. This is the only place in the paper where the authors actually did direct ic implantation of tumor cells. Was this accompanied by subcutaneous tumor cell injection simultaneously? They see evidence for B cell infiltration in these studies, but was there any clinical benefit with dual ICB in this model?”

Response:

We thank the reviewer for this important comment. Yes, the direct intracranial (i.c.) implantation experiments in Fig. 7 were performed with simultaneous subcutaneous tumor implantation. We recognize that this was not clearly stated in the Results in the original submission, and we have now explicitly described this experimental design in the revised Results/Methods and figure legend.

To address the requested “clinical benefit” in this model, we added quantitative assessment of intracranial tumor burden and survival analyses. In the i.c. implantation model, anti–PD-1 plus anti–CTLA-4 combination therapy reduced intracranial tumor burden and prolonged survival compared with isotype control, without significant body weight loss. These findings (tumor burden, survival, and body weight) were reproduced in an independent i.c. implantation model using LLC cells, supporting the robustness of the observation.

Changes made: Results revised to state simultaneous subcutaneous implantation and to report tumor burden, survival, and body weight outcomes (page 25, lines 722–730); Methods revised to clarify experimental design (page 11, lines 295–306); Figure 7 legend updated (page 36–37, lines 1053–1081); Supplementary Figure 9A–E added (page 44–45, lines 1328–1348).
